# Overcoming limitations to customize DeepVariant for domesticated animals with TrioTrain

**DOI:** 10.1101/gr.279542.124

**Published:** 2025-08

**Authors:** Jenna Kalleberg, Jacob Rissman, Robert D. Schnabel

**Affiliations:** 1 Division of Animal Sciences, University of Missouri, Columbia, Missouri 65201, USA;; 2 Genetics Area Program, University of Missouri, Columbia, Missouri 65201, USA

## Abstract

Generating high-quality variant callsets across diverse species remains challenging as most bioinformatic tools default to assumptions based on human genomes. DeepVariant (DV) excels without joint genotyping while offering fewer implementation barriers. However, the growing appeal of a “universal” algorithm has magnified the unknown impacts when used with non-human species. Here, we use bovine genomes to assess the limits of using human genome–trained variant callers, including the allele frequency channel (DV-AF) and joint-caller DeepTrio (DT). Our novel approach, TrioTrain, automates extending DV for diploid species lacking Genome-in-a-Bottle (GIAB) resources, using a region shuffling approach to mitigate barriers for SLURM-based clusters. Imperfect animal truth labels are curated to remove Mendelian discordant sites before training DV to genotype the offspring correctly. With TrioTrain, we use cattle, yak, and bison trios to create the first multispecies-trained DV-AF checkpoint. Although incomplete bovine truth sets constrain recall within challenging repetitive regions, we observe a mean SNV F1 score >0.990 across new checkpoints during GIAB benchmarking. With HG002, a bovine-trained checkpoint (28) decreased the Mendelian inheritance error (MIE) rate by a factor of two compared with the default (DV). Checkpoint 28 has a mean MIE rate of 0.03% in three bovine interspecies cross genomes. These results illustrate that a multispecies, trio-based training strategy reduces inheritance errors during single-sample variant calling. Although exclusively training with human genomes deters transferring deep-learning-based variant calling to new species, we use the diverse ancestry within bovids to illustrate the need for advanced tools designed for comparative genomics.

Machine learning applications in genomics are not new (Benzer 1959; Camin and Sokal 1965; Beyer et al. 1974). Nevertheless, the type and scale of available data continue to change what is possible through genomic-specific, deep-learning (DL) tools. Foundational, large-scale sequencing consortiums for human genomes often prompt similar work in other species. Several global efforts within the bovine genomic community run parallel to those for humans, including the 1000 Bull Genomes (1kBulls), Bovine Pangenome, and Ruminant Telomere-to-Telomere (RT2T) consortia (The 1000 Genomes Project Consortium 2010; Hayes and Daetwyler 2019; Jarvis et al. 2022; Kaminow et al. 2022; Nurk et al. 2022; Zhou et al. 2022; Liao et al. 2023; Smith et al. 2023; Kalbfleisch et al. 2024). Unfortunately, the development of species-agnostic tools has not kept pace with the exponential growth of sequenced biodiversity. Despite widespread variation in ploidy, chromosome number, or kinship, many bioinformatic tools expect the human genome by default. However, optimism surrounds DL technologies and their potential translation into non-human species (Webb 2018). Context matters when evaluating the performance of any statistical inference tool. When trained exclusively with human genomes, new data from other species can affect precision (Sundaram et al. 2018). The burden of customizing these leading-edge tools often deters application with animal genomes. Researchers of comparative genomics instead rely on human-based default parameters that may be inappropriate for other species (Genereux et al. 2020).

Human-centric tools include the most popular variant-calling software, GATK Best Practices (McKenna et al. 2010). In non-human genomics, alterations to Best Practices are common, but evaluation approaches between studies are rarely standardized. Similar pervasive inconsistencies in human genomics motivated the Genome-in-a-Bottle (GIAB) Consortium to create the first “highly accurate set of genotypes across a genome” using a multiplatform, ensemble curation approach (Zook et al. 2014). After the benefits of trio-based variant curation during assembly polishing were demonstrated in humans and cattle (Koren et al. 2018), the human GIAB resources were expanded to include complete trios (Zook et al. 2019). These resources remain the authority for assessing variant discovery methods (Chin et al. 2020; Wagner et al. 2022; Olson et al. 2023). Identifying genetic variation depends upon three components: the reference genome, the sequencing technologies, and the algorithms applied (Bickhart et al. 2020; Atkinson et al. 2023). A standardized ground truth from GIAB enables objective decisions when comparing approaches for human genomic research (Olson et al. 2022). For example, recent work found that variant discovery software can significantly impact genotype quality (GQ) compared with the alignment method (Barbitoff et al. 2022). Benchmarking competitions revealed the limits of heuristic-based variant discovery methods like GATK; in contrast, DL-based methods can identify the unknown (Sapoval et al. 2022).

For human genomic research, GIAB provides standards for communicating optimal method selection (Olson et al. 2022). However, their authoritative catalog of genomic variation has become the default source of training data for DL in genomics, such as DeepVariant (DV). As the first DL-based variant caller to achieve a small, yet significant improvement over GATK, DV's innovation stems from approaching variant calling as an image classification problem (Poplin et al. 2018a). After converting pileup alignments into multilayer tensors or channels, the convolution neural network (CNN) uses the context surrounding a putative variant to distinguish noise from truth; relevant context is encoded within a checkpoint for genotyping. DV's specialty is providing checkpoints that control the known and unknown systematic bias for a wide range of sequencing technologies, with several notable extensions. Recent work used the 1000 Genomes Project cohort to supplement DV with an allele frequency channel to create a population-aware variant caller (DV-AF) (The 1000 Genomes Project Consortium 2010; Chen et al. 2023). DV and DV-AF avoid GATK's scalability limitations by achieving comparable accuracy without requiring joint genotyping (Poplin et al. 2018b; Chen et al. 2023). As a joint caller, DeepTrio (DT) uses separate parental and offspring checkpoints to identify variants in related duo or trio samples (Kolesnikov et al. 2021). Furthermore, the haplotype-aware PEPPER–Margin–DV pipeline is a common approach for small variant detection with PacBio's long-read sequencing (Shafin et al. 2021). Despite relying heavily on the human GIAB reference materials for training, subsequent research has repeatedly demonstrated DV's superiority (Supernat et al. 2018; Zhao et al. 2020; Barbitoff et al. 2022; Lin et al. 2022; Olson et al. 2022). DV's success inspired the rapid development of new methods for variant discovery (Sahraeian et al. 2019; Ip et al. 2020; Luo et al. 2020; Ramachandran et al. 2021; Khazeeva et al. 2022; Su et al. 2022; Ahsan et al. 2023; Popic et al. 2023). Although DL technologies saturate the leading edge of genomics, most overlook the challenges facing non-human genomics, limiting their relevance across biological diversity.

The revolutionizing impact of DL-based approaches is attributed to publicly accessible genomic data and ground-truth variant callsets (Olson et al. 2023). Unfortunately, these resources only exist for human genomes, stagnating tool development for other species. Early research applied DV in mice and rice to demonstrate the variant caller's translational capabilities (Poplin et al. 2018a; Day and Poplin 2019). However, differences in variant density and distributions of nucleotide content impact DV's ability to perform equally across species (Yun et al. 2018; Sapoval et al. 2022). Custom DV checkpoints are necessary to adjust for distributional changes relative to prior experience with the human genome. Until now, their development has been driven by substantial divergence between the training and the target species, such as mosquitoes and Kākāpō parrots (Yun et al. 2018; Guhlin et al. 2023). Despite starting with the human-based DV, these resulting checkpoints are considered “species specific” because customization across taxonomy may inhibit generalization with humans. As the “default” genome, models exclusively trained with human genomes are implicitly trusted in other species; the customization for Kākāpō parrots relied on truth labels created with DV (v0.9) (Guhlin et al. 2023). Recent work in cattle also relied on defaults intended for human genomes for both DV and GATK (Lloret-Villas et al. 2021, 2023). They argue DV is superior to GATK for bovine short-read sequencing (SRS); however, their conclusions were based on a prevalent limitation within animal genomics: expecting the GATK Best Practices to be optimal across species. Because of sample size (N ∼ 500), their approach with GATK required manually selecting site-level thresholds (hard-filtering). Despite these nuances, DV has seen a rapid expansion in comparative genomics, including domesticated and endangered animals, plants, and pathogenic organisms (Gunderson et al. 2020; Osipowski et al. 2020; Stenløkk et al. 2022; Flack et al. 2023; Guhlin et al. 2023; Jesudoss Chelladurai et al. 2023; Leonard et al. 2023; Ruperao et al. 2023; Secomandi et al. 2023). Researchers studying a wide range of species are drawn to DV because of substantially fewer implementation barriers than GATK.

Here, we address the nontrivial technical barriers of customizing DV for species lacking GIAB-quality truth callsets using TrioTrain, which enables automatic training extension with new data on a SLURM-based computing cluster (Supplemental Fig. S33). Existing trio-based joint-calling companion software (dv-trio; DeepTrio) requires samples with a known pedigree during training and genotyping (Ip et al. 2020; Kolesnikov et al. 2021). In contrast, the curriculum within TrioTrain indirectly encodes inheritance expectations by training simultaneously with two genomes that happen to be parent–offspring duos to produce a single-sample variant caller. We release the current version of our customized, trio-based, multispecies-trained DV-AF checkpoint, which reduces inheritance errors in human and bovine genomes. Our research illustrates the limitations of using the default, human genome–trained DV in other species. Although we demonstrate how to repurpose existing non-human genomic data for DL, we emphasize that maximizing the quality of models built with TrioTrain will require expanding the number of organisms with comprehensive and accurate variant benchmarks.

## Results

### Initial variant quality indicated a training gap

As mosquito and Kākāpō genomes are appreciably more diverged from humans than cattle, we initially expected retraining to be unnecessary. For our preliminary evaluation, we used one bovine genome (NCBI BioSample [https://www.ncbi.nlm.nih.gov/biosample] accession number SAMN10940470) to compare single-sample calling with DV (v1.0) against an existing bovine callset from multisample calling with GATK (v3.8). These data were created during the 1kBulls project, a global consortium that standardized methods for population-based analyses with cattle (Hayes and Daetwyler 2019). Three runs were produced after the release of the current reference genome: ARS-UCD1.2 (Rosen et al. 2020), with Run7 in 2019 (N = 3818), Run8 in 2020 (N = 4931), and Run9 in 2021 (N = 6191). Over time, detection power improved with the increasing sample size, resulting in “the largest sequence repository of bovine genomic variation” (Dorji et al. 2024). Unlike the typical hard filtering in animal genomics, the sample size of the 1kBulls cohort enabled variant quality score recalibration (VQSR) to reduce systematic error. Although some of these data are publicly available, they are not analogous to the GIAB variant benchmarks.

In animal genomics, method evaluation typically relies on indirect quality metrics owing to slight variations in context and software across studies. For example, we initially assessed the two variant-calling methods using existing data produced during 1kBulls Run8 (2020). For GATK (v3.8), a single-sample VCF was extracted from the larger Run8 cohort. For DV (v1.0), a single-sample VCF was generated by providing the same BAM file as GATK. Then, the two approaches were compared using BCFtools isec (v1.15) (Danecek et al. 2021), constrained to Chromosome 29. We inferred variant quality using the transition-to-transversion (Ti:Tv) ratio, in which values below the expected genome-wide ratio of 2.0–2.3 are interpreted as lower quality (Crysnanto et al. 2019). We found variants unique to the population-scale, VQSR-filtered GATK callset achieved a marginally higher Ti:Tv ratio (+0.14 points) (Supplemental Fig. S1), suggesting variants from DV were lower quality relative to the 1kBulls Run8 cohort. Based on these findings, we began developing TrioTrain to explore if DV could benefit from further training with bovine genomes.

Recently, a small cohort of dairy cattle (N = 50) was used to compare callsets resulting from GATK (v4.2) and DV (v1.2) (Lloret-Villas et al. 2023). Although they also observed more unique variants with DV than with GATK, they reported a slight increase in Ti:Tv with DV (+0.04 points) than with GATK after manually selecting site-level filtering thresholds (hard filtering) (Supplemental Table S1). Given the contradictory results across studies, we repeated our evaluation between GATK (v3.8) and DV (v1.4), using Ti:Tv to measure variant quality indirectly. For this analysis, we identified a subset of taurine cattle from the 1kBulls cohort with publicly available SRS data (N = 13) (Supplemental Table S1). We performed single-sample calling with DV to compare against joint-calling with GATK with VQSR filtering (UMAGv1 callset; see Methods). Relative to our initial evaluation, mean Ti:Tv increased for concordant variants (+0.26 points). Although unique variant quality with DV increased (+0.20 points), the mean genome-wide Ti:Tv ratio was slightly higher (+0.06 points) with the UMAGv1 GATK-based callset. These contradictory results demonstrate the limitations of relying on indirect quality metrics for species lacking variant benchmarks.

### Label inconsistency limits model quality

The challenge for training species-specific models is maximizing the quality of the labels used as truth. Here, we infer truth using SRS data and GATK, which introduces a small amount of noise that is indistinguishable from genuine variation. The initial build of TrioTrain relied on truth labels from the 1kBulls Run8 callset (Supplemental Note S1). After adapting training to our SLURM-based HPC cluster, we assessed these existing data as potential truth labels by filtering to increase specificity. During these preliminary analyses, we reviewed plots for model loss, an indicator of prediction error that training seeks to minimize (Supplemental Fig. S2). Despite applying more strict filters (GQ > 30), the loss hovered consistently around 0.2 instead of approaching zero. Subsequent testing discovered an order-of-operations flaw during filtering, creating inconsistencies between the truth VCF and the PopVCF. These results indicated overfitting and potentially noisy labels. Based on these findings, we excluded the 1kBulls Run8 callset as potential training labels; however, subsequent analysis improved upon these existing standards for population-scale variant calling in cattle using GATK.

We began by examining genotyping consistency between the three 1kBulls genotyping runs (2019–2021) by selecting samples included in all three joint-calling runs (N = 3659; mean coverage, 13.7×). From each cohort VCF, a sample's PASS genotypes were extracted into a single-sample VCF, enabling a pairwise comparison using established methods (Krusche et al. 2019). To our knowledge, this is the most extensive comparison with these data, requiring >1.5 million CPU hours (Supplemental Note S2). In theory, the genotypes for a sample should be identical (F1 score >0.990); instead, we observed lower recall and precision across the 1kBulls runs than expected (mean F1 scores between 0.95–0.97) (Supplemental Table S2). These findings reveal variability between genotypes derived from the same underlying sequence data, affecting all downstream analyses that assume accurate and consistent genotypes. Given these limitations, we improved upon the 1kBulls variant-calling protocol by iteratively altering parameters during VQSR, resulting in a new variant callset (UMAGv1). Animal genomics typically settle for performing VQSR once or for hard-filtering, using parameters intended for human genomes (Lloret-Villas et al. 2023). Instead, we tested a range of values for each tunable parameter, resulting in about 300–1000 VQSR runs. From these analyses, we selected VQSR parameter values that produced fewer violations of Mendelian inheritance within bovine trios while increasing the total number of PASS variants. We then compared our optimal parameters against the defaults and the 1kBulls Run9 (2021). The new UMAGv1 callset contains more PASS variants and 2× fewer Mendelian inheritance errors (MIEs) (Supplemental Methods; Supplemental Fig. S30). Although our research demonstrates current boundaries for producing higher-quality variant callsets in animal genomics, we provide further evidence that the default values intended for human genomes introduce error when used in other organisms. Relative to the 1kBulls Run9, the quality improvements within the UMAGv1 callset enabled us to reconsider extending DV with bovine genomes.

### Building TrioTrain to design a bovine-specific curriculum

A lack of high-quality benchmarking variants has thwarted most pursuits for non-human DV models. Previous extensions of DV focused on nonmammalian diploid organisms while altering the training curriculum (for a comparative summary, see Supplemental Table S3; Yun et al. 2018; Guhlin et al. 2023). For example, in the mosquito-specific approach, the parental genomes were never given to DV but instead used to curate inherited variants within the offspring (Yun et al. 2018). Our approach modifies the mosquito-specific strategy based on pedigree structure, genome length, and sample size. For cattle, violations of Mendelian inheritance patterns were attributed to systematic error during SRS because de novo variants are comparably rare (Harland et al. 2017; Lee et al. 2023). Based on these expectations, our trio-based variant curation excluded sites violating Mendelian inheritance expectations within a complete bovine trio. Relative to mosquitoes, we hypothesized that the larger sample size of reported pedigrees across bovids could potentially offset imperfect truth labels. However, agricultural genomics historically prioritized larger sample sizes with lower coverage. Because DV-AF had improved accuracy in lower coverage human SRS samples, we include population allele frequencies from the UMAGv1 callset when creating new models with TrioTrain (Chen et al. 2023).

Using the reported pedigree from public SRS data, we identified known families within the UMAGv1 cohort, resulting in 15 complete trios from multiple bovine species (Table 1). With these trios, we built 30 bovine-trained DV-AF checkpoints sequentially over five phases, exploring potential strategies for customizing DV. Our trio-based experimental design structures the training phases into biological questions to provide insight into DV's behavior. For example, as with prior nonmammalian approaches, our training strategy during the first two phases focuses on creating a “cattle-specific” model, using data typical for domesticated animal genomics. Training begins with six cattle trios representing multiple unique cattle breeds, whereas the second phase adds breed-specific replicates. Next, we investigate if DV's species-specific experience could be extended for comparative genomics because accuracy in humans previously increased with diversity (Chen et al. 2023). The third phase uses two bison trios with the same father to determine whether a multispecies, “bovine-specific” model was possible. The final two phases maximize truth-label confidence, demonstrating the value of a potential cattle-specific, GIAB-quality resource. The boundaries of quality are rapidly developing as the Q100 project works toward a perfect, diploid, telomere-to-telomere (T2T) genome assembly from a GIAB sample (HG002) (https://genomeinformatics.github.io/HG002v1/; Rautiainen et al. 2023). The potential for minimal per-base error for both haplotypes is shifting GIAB's previous read-reference variant benchmarks toward assembly-reference benchmarks. For cattle, we relied on the ongoing Bovine Pangenome project, which uses trio-binning to produce haplotype-resolved alternative reference assemblies. Multiple platforms were used to sequence three F_1_ crosses with extant bovine lineages, including the *taurus* and *indicus* subspecies, Angus–Brahman (Low et al. 2020), and two interspecific crosses, Highlander–yak and Simmental–bison (Rice et al. 2020; Heaton et al. 2021; Oppenheimer et al. 2021). During our analysis, these reconstructed haploid parental assemblies represented the highest-quality assembly-based truth set for cattle (Supplemental Methods; Supplemental Table S11). However, the increased heterozygosity in these hybrid offspring challenges DV's capabilities with genotype class imbalance (Supplemental Table S4). Therefore, phase 4 strategically uses these data after exhausting standard cattle and bison trios. Given the prohibitive curation effort required to build the original GIAB resources, we considered the potential of using synthetic truth labels in animal genomics. For phase 5, we used synthetic SRS reads created for the Angus × Brahman hybrid offspring (Methods). As publicly reported pedigree records will restrict other animal species, we contrast each phase of our multitrio approach against three single-trio replicates. The first represents a pragmatic scenario of repurposing a trio sequenced only with SRS (checkpoint 2). Lastly, we perform two parallel single-trio experiments, resulting in four additional bovine-trained DV-AF checkpoints. These represent an alternative strategy of training with the highest-quality bovine truth labels immediately, effectively skipping the first 20 iterations to only extend DV with the original SRS data from a hybrid trio, either the Angus–Brahman or the yak–Highlander (checkpoints 2B and 2C, respectively).

**Table 1.** Bovine genomes used during successive TrioTrain iterations

		Training: Parents				Evaluation: Offspring			
	Trio name	SRA accession	Sex	Breed code	Mean cov.	SRA accession	Sex	Breed code	Mean cov.
Training phase 1									
1	Trio1	SAMN10940502	M	AA	35.96	SAMN10940538	F	AA	23.64
2		SAMN10940537	F	AA	23.10				
3	Trio2	SAMN10598569	M	HE	17.47	SAMN10598563	F	HE	14.45
4		SAMN10598566	F	HE	12.92				
5	Trio3	SAMEA4644756	M	BS	15.84	SAMEA4644754	F	BS	16.78
6		SAMEA4644755	F	BS	15.14				
7	Trio4	SAMN15780082	M	HO	35.23	SAMN15779741	M	HO	23.66
8		SAMN15779919	F	HO	46.68				
9	Trio5	SAMN10598570	M	HE	13.51	SAMN10598562	F	HE	15.89
10		SAMN10598567	F	HE	15.08				
11	Trio6	SAMEA5159887	M	TG	21.63	SAMEA5159888	F	TG	20.41
12		SAMEA5159889	F	TG	19.77				
Training phase 2									
13	Trio7	SAMN15779644	M	HJ	39.35	SAMN15779593	F	HJ	50.83
14		SAMN15779569	F	HJ	44.75				
15	Trio8	SAMN15779606	M	HJ	30.81	SAMN15779717	M	HJ	27.65
16		SAMN15779596	F	HJ	39.31				
17	Trio9	SAMN10598569	M	HE	17.47	SAMN10598564	M	HE	14.79
18		SAMN10598565	F	HE	14.62				
Training phase 3									
19	Trio10	SAMN05788493	M	BI	22.69	SAMN13655887	F	BI	17.95
20		SAMN13655886	F	BI	23.72				
21	Trio11	SAMN05788493	M	BI	22.69	SAMN10940703	M	BI	24.06
22		SAMN10940702	F	BI	23.78				
Training phase 4									
23	Trio12	SAMN08473804	M	AA	36.99	SAMN08473802	M	F_1_X	50.01
24		SAMN08473803	F	BR	43.32				
25	Trio13	SAMN12153485	M	HI	34.69	SAMN12153487	F	F_1_X	17.92
26		SAMN12153486	F	YK	10.66				
27	Trio14	SAMN16823422	M	BI	27.44	SAMN16780309	M	F_1_X	14.46
28		SAMN16825967	F	SI	35.42				
Training phase 5									
29	Trio15	SAMN08473804	M	BI	36.99	SYNTHETIC	M	F_1_X	26.20
30		SAMN08473803	F	SI	43.32				

Individual genomes have a distinct “truth” VCF. Extending DeepVariant with bovine genomes was achieved over five phases with 30 TrioTrain iterations, representing 14 family trios and one synthetic hybrid offspring. Additional details are provided in Supplemental Table S4. Breed code abbreviations are as follows: (AA) Angus, (HE) Hereford, (BS) Brown Swiss, (HO) Holstein, (TG) Tyrolean Grey, (HJ) Holstein–Jersey, (BI) bison, (BR) Brahman, (HI) Scottish Highlander, (YK) yak, (SI) Simmental, and (F_1_X) F_1_ hybrid cross.

**Figure 1.**
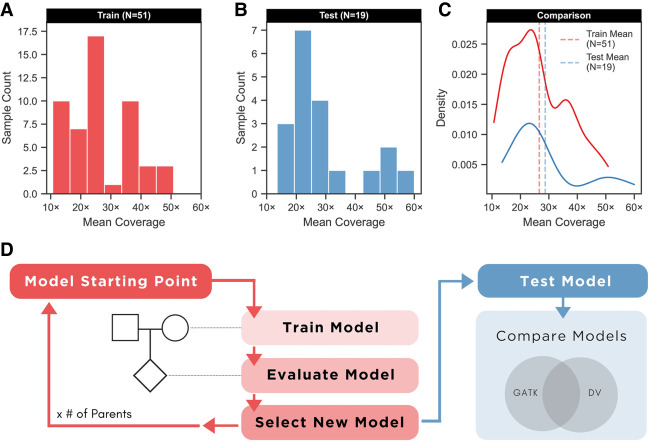
Before extending DeepVariant (DV), the available data were assigned to different purposes: training (*A*) or testing (*B*). The *x*-axis provides thresholds for genome-wide mean coverage, and the *y*-axis provides sample counts. (*A*) We selected samples from the UMAGv1 cohort with complete pedigree information (family trios). With TrioTrain, DV simultaneously uses labeled examples from two genomes: A parental genome provides new information, whereas learning is evaluated based on genotype predictions in the offspring. (*B*) Most samples used for model testing (13/19) lacked a complete pedigree but served as an independent test set because their GATK-based truth labels were withheld from DV during training. (*C*) The kernel density estimate (KDE) plot allows for comparing the two distributions after normalizing for sample size. (*D*) Workflow diagram of TrioTrain's iterative retraining process. All retraining begins with weights from an existing checkpoint (warm-starting); our experiments began with DV (v1.4) without the allele frequency channel. After initialization, all bovine-trained checkpoints include the allele frequency channel (DV-AF), encoding the PopVCF from the UMAGv1 cohort. Two iterations are performed for each trio, one for each parent. An iteration covers all labeled examples within a bovine parent–child duo constrained to sample-specific callable regions from Chr 1–29 and X. Each iteration produces a candidate checkpoint that is then assessed using a set of test genomes (N = 19). The resulting variant calls are compared against sample-specific, GATK-derived, truth labels to monitor training behavior over time.

With TrioTrain, DV is given an entire trio after two sequential iterations, one for each parent (Methods). Within a trio, both iterations use the same labeled examples from the child to select an optimal checkpoint that distinguishes inherited variants from noise. TrioTrain automatically splits each trio into parent–offspring duos; the parental genome provides new context during training, which is continuously evaluated using the offspring, also known as tuning (Fig. 1A). When provided a set of genomes previously withheld from training or evaluation, the pipeline will automatically estimate generalization, known as testing. These bovine genomes (N = 19) (Fig. 1B) include real SRS from the offspring of the three F_1_ crosses, three corresponding offspring replicates with synthetic SRS, along with 13 nonpedigreed taurine cattle that remain independent because truth labels are withheld from DV (Supplemental Table S5). Bovine data were selected to be representative of publicly available SRS, with similar mean coverage (Fig. 1C). TrioTrain is designed to enable training with any diploid vertebrate species and begins by initializing weights or warm-starting from the existing human-trained DV checkpoint (Fig. 1D). Bovine truth sets used for either training, evaluation, and testing were constrained to the autosomes and X Chromosome and then restricted with a sample-specific callable-region BED file generated by GATK (Methods). As such, the UMAGv1 bovine truth sets are more conservative than similar files provided by GIAB. The number of steps during a training iteration varies based on the example variants produced for a parent; within each trio, all training examples are used once to prevent overfitting. Tuning with the offspring continuously calculates stratified performance metrics. Pedigree information is not explicitly provided to DV; instead, the checkpoint that performs best with the offspring is selected as the warm-starting point for the next iteration. Then, the entire retraining process is iteratively repeated until all trios have been exhausted.

### Factors that influence training behavior

**Figure 2.**
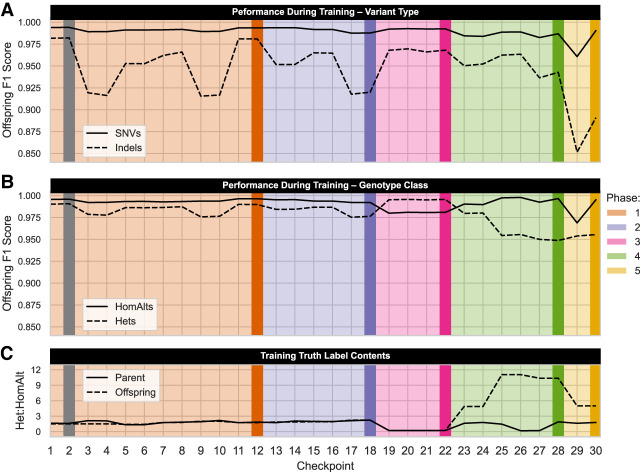
Training performance across successive iterations with bovine trios reveals insights into DV's behavior. The darker vertical lines highlight the checkpoints (2, 12, 18, 22, 28, 30) used in subsequent analyses to compare across phases. TrioTrain enables structuring the bovine inputs to match inheritance expectations; variation is transmitted from parents to offspring. Pedigree data are not explicitly given to DV; instead, parental genotypes are used to inform predictions in the offspring. The number of checkpoints (*x*-axis) represents the number of parental genomes given to DV (odd, paternal; even, maternal). Two iterations are required to cover both parents. For example, iterations 1 and 2 represent a complete bovine trio in which truth labels from a single offspring were used to evaluate the optimal training stopping point (Table 1). In the first two panels, the *y*-axis represents the maximum F1 score achieved in the offspring; comparing across iterations reveals if DV found the parental truth labels informative. Each line represents stratified performance by different variant classifications. (*A*) Variant type (SNVs, indels). As expected, training struggles with indels owing to imbalanced classification, as the bovine truth labels contain more SNV relative to those from humans. (*B*) Genotype class (HomAlts, Hets). Between iterations 18 and 19, we observe a shift in the best-performing genotype class for the offspring owing to distributional changes to heterozygosity. (*C*) Truth label contents contribute to training performance. Phase 3 uses truth labels from bison genomes aligned to the cattle reference; a sudden increase in HomAlts (Supplemental Fig. S3) is reflected by the decreased Het:HomAlt ratio. Counterintuitively, increasing the number of HomAlt examples given to DV contributes to a decrease in F1 score for that genotype class during training.

When used appropriately, DL-based methods are superior because they can account for the numerous subtle factors that alter distributions within the data. However, the nature of DL typically obscures which features are relevant for accurate prediction; previous work suggests the importance of demographic history, variant distribution and density, and genome complexity (Zou et al. 2019; Musolf et al. 2022; Whalen et al. 2022). With TrioTrain, the metrics from training and evaluation indicate if the parental truth labels helped DV accurately predict the offspring's genotypes. However, our ability to estimate training performance depends on the definition of truth. False positives (FPs) include any variant discovered by DV that GATK misses. Variants DV ignores within the UMAGv1 truth labels are considered false negatives (FNs). True positives (TP) are an exact match between DV and GATK. These definitions are essential caveats as FP errors may be novel variants missing from the GATK-based truth labels. We measure performance using the harmonic mean of precision and recall, known as the F1 score (TP/TP + ½ [FP + FN]), where a value of one indicates perfect prediction. Because model selection relies on F1 score, we compared the successively created bovine DV-AF checkpoints stratified by variant type (Fig. 2A) and genotype class (Fig. 2B). Performance assessment of TrioTrain starts within a trio (*x*-axis: odd–even pairs). For example, in Figure 2A, comparing two checkpoints (27–28) evaluated using the same offspring reveals that maternal examples (28) slightly improved prediction within the offspring relative to only paternal examples (27). Alternatively, comparing performance between trios by reviewing patterns across several checkpoint pairs (i.e., 3–4 vs. 11–12) helps reveal bovine genome characteristics that alter DV's behavior, indicating a distribution change relative to the GIAB trios (Supplemental Table S6).

Building the mosquito-specific DV revealed that the default checkpoint struggled with higher variant density; notably, cattle have ∼40% more nucleotide diversity and about 1.5× more small variants compared with humans despite similar genome lengths (Gibbs et al. 2009). Other factors, such as the ratios for variant type and genotype class, reflect subtle nuances learned by DV. Essentially, DV anticipates a certain level of imbalanced classification from the human GIAB samples (Supplemental Table S6). With balanced data, the ratio between predicted classes approaches one. In contrast, training with GIAB truth sets gave DV more than 6× more SNV examples (mean SNV:indel, 6.58). Cattle are expected to have similar distributions (SNV:indel, 7.05) (Crysnanto et al. 2019), which is reflected during the first 18 iterations of TrioTrain (mean SNV:indel, 7.54) (Supplemental Tables S6, S7). However, the diversity of the bovine trios in subsequent iterations provides substantially more SNVs (mean SNV:indel, 9.02) relative to DV's expectations. Training performance over multiple bovine genomes reflects how these data deviate from the established human-genome priors.

Multiple factors contribute to the priors learned by DV. Heterozygous variant confidence decreases with coverage (less than 30×) because fewer reads at a locus can introduce allele sampling bias. With SRS, indels are notoriously challenging because mapping accuracy declines for reads that cannot span the variant. By relying on a single sequencing platform, some bovine indels detected by DV will be incorrectly labeled as errors (FPs) simply because they are missing from the bovine truth set. Despite these caveats, overall performance across all iterations is robust, with a median optimal checkpoint F1 score of 0.9865 (Supplemental Table S8). The training performance observed during phases 1 and 2 demonstrates that high-quality variant callsets derived from existing, population-scale SRS can be curated into “silver-standard” training data. However, this required exhaustively improving joint-calling accuracy before training; therefore, we caution that our findings represent an upper limit threshold rather than typical performance with non-human SRS data. Across the 30 bovine DV-AF checkpoints, the F1 score for SNVs approaching one (Fig. 2A) indicates that DV successfully uses the parent genome to inform offspring variant calling (median SNV F1 score, 0.991), with one iteration (29) deviating from the others. Phase 5 begins with replicating the parental genome in the previous phase (23); however, the offspring used for model tuning were inspired by pseudodiploid (SynDip) benchmarking strategies in humans (Li et al. 2018). Synthetic SRS reads were sampled from the reconstructed parental haplotype assemblies, resulting in a higher per-base error rate than the offspring's Illumina sequencing data. Because we do not intend to call variants in synthetic data, we halted training with other synthetic offspring samples.

Investigating why some bovine genomes underperform during training provides more insight into the human training curriculum. Previous work found that including the allele frequency channel (DV-AF) stabilized performance in lower-coverage human genomes (Chen et al. 2023). Because allele frequencies from the UMAGv1 cohort were included during training, our bovine-trained DV-AF should handle lower coverage genomes relatively well. As expected, regardless of parental coverage being above or below 30×, we observe robust offspring F1 score (Supplemental Fig. S4E), with most checkpoints achieving high recall (fewer FNs) during training. Performance with indels was lower than SNVs, which is expected given how the bovine truth labels were curated (Methods). Although variable coverage has minimal impact on offspring SNV precision (Supplemental Fig. S4C), six lower-coverage parents produce a cluster of checkpoints with decreased training indel precision (Supplemental Fig. S4D).

However, interpreting change during training from a discrete attribute such as coverage overlooks DV's ability to learn complex and hidden interactions. The cluster of checkpoints indicates that training underperforms specifically with parents from the same breed as the reference genome (Hereford [HE]) and lower coverage (less than 20×) (Rosen et al. 2020). Unlike the composite human reference genome (GRCh38), the cattle reference assembly represents a single, highly inbred cow named Dominette. This reference captures most variation between Hereford individuals, typically resulting in fewer homozygous alternate (HomAlt) genotypes relative to cattle from other breeds. Although the raw variant count of HomAlts for Hereford cattle is similar to the GIAB genomes (mean HomAlt/genome: humans, about 1.60 million; HE cattle, about 1.68 million), the species difference is more pronounced for Hets (mean Hets/genome: humans, about 2.3 million; HE cattle, about 3.4 million). Relative to other cattle breeds used during training, the truth labels for the HE offspring contain proportionally more Hets (Supplemental Table S7), making it imperative for DV to experience similar data during training. Gravitating toward “breed-specific” or “species-specific” explanations may be intuitive; however, DV remains unaware of those labels. Instead, these differences become evident to DV through novel characteristics within the provided training callsets.

Training DV with bovine genomes shifts several distributions of the predicted classes. For example, the Het:HomAlt ratio is influenced by depth of coverage and variant density. Deviating from the priors of the human GIAB samples (mean Het:HomAlt, 1.46) during training is one way for DV to gain new experience. For example, aligning bison genomes to the cattle reference, on average, leads to nearly 9× more HomAlt genotypes in the truth set relative to previous iterations with cattle (mean Het:HomAlt, 0.21) (Supplemental Fig. S3E; Supplemental Table S6). The lower training recall (Supplemental Fig. S5F) and the corresponding decrease in offspring F1 score (Supplemental Fig. S5B) for the minor class (HomAlts) during phase 3 are expected because the bison truth labels contain proportionally more HomAlts. A similar performance shift occurs when training with the interspecific crosses during phase 4; the hybrid offspring provide nearly 6× more Het examples than all previous offspring (Supplemental Table S6). DV must adapt to the increased heterozygosity in the hybrid offspring relative to the parents (Fig. 2C). Increasing the examples from the major class (Hets) (Supplemental Fig. S3D) leads to a corresponding recall decrease (Supplemental Fig. S5E). These shifts in training performance for specific classes reflect the bovine training data diverging from the human-based priors, a necessary step to build new experience. Our results indicate that truth variants from related species can provide additional context for accurate variant discovery.

### Assessing generalization across species

Recommending a TrioTrain checkpoint for variant calling requires confirmation of accurate genotype predictions outside the training data, known as model testing. We assessed the 30 new bovine-trained DV-AF checkpoints created during sequential iterations with TrioTrain against existing versions (DV and DV-AF). These checkpoints were first tested using a multibreed set of taurine cattle genomes (N = 13) that were not used previously during either training or tuning (Supplemental Table S5, top). Unlike training, the truth genotypes for these samples are withheld from DV but do not exclude Mendelian discordances owing to incomplete pedigree or missing parental genotypes. Applying the existing human-trained DV to cattle genomes does not severely decrease testing recall (Supplemental Fig. S6), indicating that priors from the human genome are informative for genotyping cattle. However, a sudden decline in testing recall occurs when using Angus paternal examples to predict variation in the Angus–Simmental hybrid cross offspring. During this iteration (23), DV recalls Hets (Supplemental Fig. S6C) at the cost of missing SNVs, indels, and HomAlts. With our trio-based training approach, testing recall recovers after the next iteration (24) provides maternal examples. The limitations of the UMAGv1 truth labels become apparent when comparing precision and recall within a particular variant type. Testing precision increases slightly between iterations 1 and 28 for indels (Supplemental Fig. S6B). Because fewer indel examples are provided per training iteration relative to SNVs, more iterations are required to accumulate the same volume of examples. However, the corresponding decrease in testing recall for the same class indicates that the bovine truth set is overly conservative (fewer indels).

As expected, single-sample variant calling with existing human-trained checkpoints introduces variants relative to the bovine GATK-based truth labels (UMAGv1), resulting in low testing precision (Supplemental Fig. S6) and F1 score (Supplemental Fig. S7). Further exploration of these putative FPs focused on a single genome, the same individual as the cattle reference (BioSample SAMN03145444). Variants missing from the bovine truth set correctly identified by the human-trained DV include Het SNVs within homopolymers (Supplemental Fig. S8) or BovB repetitive regions (Supplemental Fig. S9). Including the UMAGv1 allele frequencies reduced HomAlt errors in low-coverage regions (Supplemental Fig. S10) as intended: Extending DV with a single cattle genome (iteration 1) results in a 4.88-fold reduction in these HomAlt errors. Although some FPs remain with an altered genotype (16%), a manual review of HomAlt SNVs introduced by the default checkpoint that were resolved after the first TrioTrain iteration confirmed that training was necessary. Heterozygous variant density likely contributed to some actual genotyping errors made by DV (Supplemental Figs. S11, S12). Adjusting the heterozygosity priors learned from humans by training in cattle also improves genotyping error from DV (Supplemental Figs. S13, S14).

**Figure 3.**
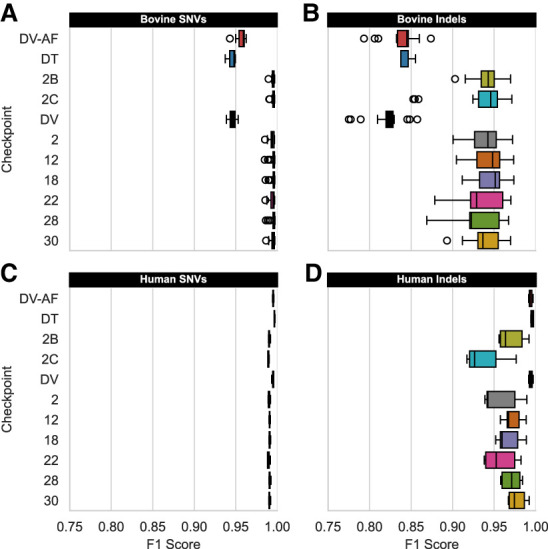
Comparing variant type generalization across species. Three human-trained versions of DeepVariant (DV, DV-AF, DT) are contrasted against eight bovine-trained DV-AF checkpoints created with TrioTrain. Each box-and-whisker represents the distribution of F1 scores observed in bovine testing genomes (*A,B*; N = 19, except for DT, where N = 3 offspring) or the human GIAB trios (*C,D*; N = 6). As expected, exclusively training with the human GIAB samples achieves a higher F1 score with these samples. Although bovine-trained checkpoints ignore genuine indels in humans, the high-quality SNV variants within the UMAGv1 callset enable SNV generalization across species. However, bovine genomes (*A,B*) results quantify genotyping changes relative to the GATK-based truth (UMAGv1). For results stratified by genotype class, see Supplemental Figure S15.

The relative similarity between bovine and human genomes enables comparison across taxonomies unreported by prior nonmammalian extensions of DV (Yun et al. 2018; Guhlin et al. 2023). Given the potential ambiguity with the nonpedigreed bovine testing genomes, they were supplemented with the three real and three synthetic bovine offspring, along with the six human genomes (Supplemental Table S5, bottom). Although the truth labels for these six bovine offspring represent the highest-confidence truth sets within the UMAGv1 cohort, we can only describe changes to generalization relative to GATK. In contrast, the GIAB trios enable definitive benchmarking as previously described (Krusche et al. 2019). Six checkpoints built with TrioTrain were selected to simplify comparison between species and across TrioTrain phases (Fig. 2, bold vertical lines). The performance with these multitrio checkpoints (Fig. 3) is contrasted against two additional single-trio checkpoints (Fig. 2B,C; Supplemental Fig. S16), along with existing versions of the joint-caller (DT) and the two single-sample, human-trained versions (DV and DV-AF). Given that the UMAGv1 truth set is expected to miss some indels, models built with these data are expected to do the same in bovine (Fig. 3A,B) and human genomes (Fig. 3C,D). All eight bovine-trained checkpoints generalize well in both species for all other classifications relative to indels (Supplemental Fig. S15). However, out of the eight bovine DV-AF checkpoints, checkpoint 28 (model.ckpt-282383) maximized SNV F1 score in the bovine (max, 0.997239; N = 19) and human (max, 0.992415; N = 6) samples.

**Figure 4.**
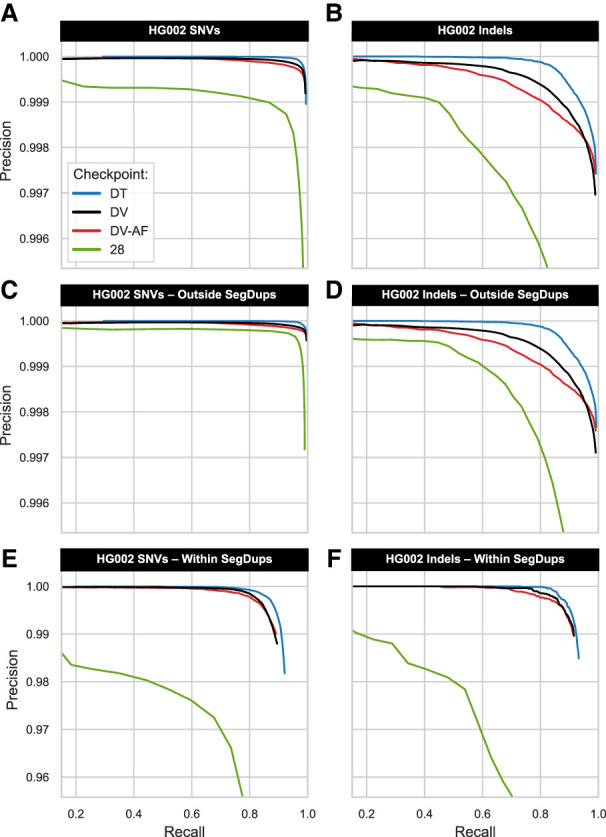
Benchmarking checkpoints with HG002. We used hap.py to calculate the precision-recall curve in a single human genome (HG002), where values above 0.5 indicate skilled prediction. We contrast three human-trained versions of DeepVariant (DV, DV-AF, DT) against a bovine-trained DV-AF checkpoint (28) that generalized well in humans and cattle. The *top* panels stratify genome-wide classification accuracy in SNV (*A*) or indels (*B*), controlling for the variable number of genotypes to allow direct model comparison. The *middle* panels use the GIAB stratifications (v3.5) to compare classification accuracy outside known segmental duplication (SegDup) regions in SNV (*C*) and indels (*D*). Note that the *y*-axis has a different scale for the *bottom* panels for stratification within SegDups in SNV (*E*) and indels (*F*). Models exclusively trained with human genomes outperform the bovine-trained model in HG002. However, the TrioTrain checkpoint's lower performance in repetitive regions is expected because bovine SegDups are not characterized to the same extent as in humans. Outside of known SegDups in HG002, the bovine-trained checkpoint created with TrioTrain is nearly perfect in SNVs (*C*). We infer that training with bovine genomes alters DeepVariant's priors for heterozygosity and copy number variation; these adjustments contribute to the marginally lower curve observed in human genome-wide precision and recall (*top*).

During benchmarking with the GIAB trios, the best-performing TrioTrain checkpoint expectedly underperformed relative to those exclusively trained with those samples (Supplemental Fig. S4A,B). Genome-wide performance with the Ashkenazi Jewish (AJ) offspring HG002 confirmed that a multitrio approach outperforms all single-trio checkpoints (Supplemental Fig. S16). The slight decrease during human benchmarks is primarily owing to training with a different definition of truth. For example, bovine truth labels were constrained to a sample-specific callable region BED file (Methods). Although the current versions of the GIAB truth sets include complex and challenging regions, the bovine truth sets are constrained to variation detected confidently with SRS, limiting the amount of novel, difficult examples. Challenging or repetitive regions are not excluded because they are not characterized to the same extent as in humans. As such, the bovine-trained checkpoints have lower recall in complex and repetitive regions, regardless of species. Outside known segmental duplication regions in HG002, the bovine-trained checkpoint (28) is nearly perfect in SNVs (Fig. 4C) and substantially improved for indels (Fig. 4D). Lower performance within segmental duplications (Supplemental Fig. S4E,F) indicates that training with bovine genomes alters DV's expectations of copy number variation in addition to the heterozygosity priors. Without a definitive ground truth specific to bovine genomes, selecting the optimal checkpoint required further analyses.

### Fewer MIEs without joint genotyping

**Figure 5.**
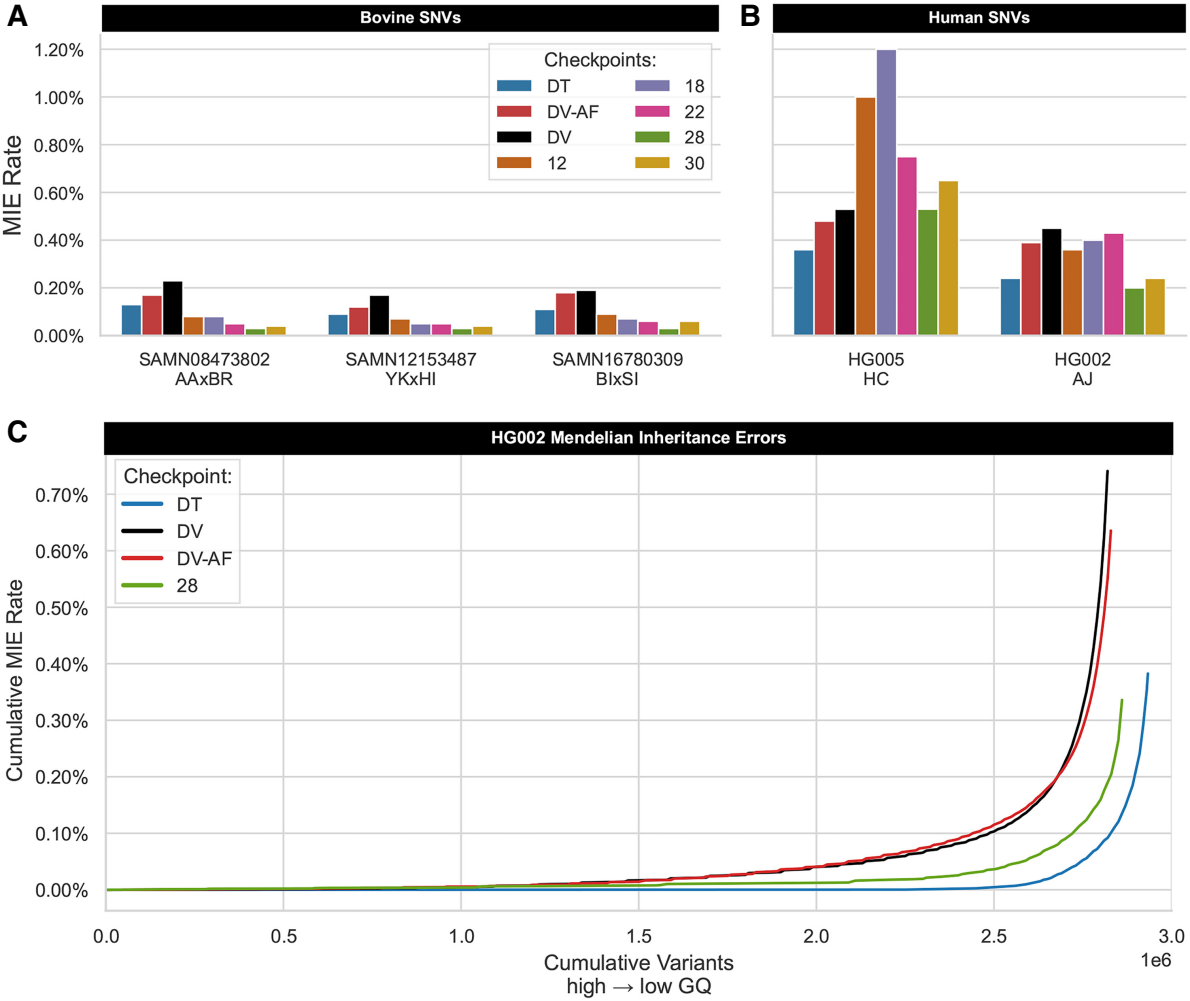
Assessing inheritance error rate in human and bovine trios. Mendelian inheritance errors (MIEs) were identified in PASS variants from the autosomes and X Chromosome for three bovine hybrid-cross trios (*A*) and two GIAB human trios (*B*). Each cluster of bars on the *x*-axis represents a distinct trio, with a bar representing the total number of discordant SNVs proportional to the total number of variants analyzed for eight distinct checkpoints. The Han Chinese (HC) trio deviates from all other trios, with the MIE rate nearly doubling for the first two TrioTrain phases. However, relative to the default checkpoint (DV), one bovine-AF checkpoint (28) achieves an identical SNV MIE rate in HG005 (0.53%) while also producing fewer discordant SNVs in the Ashkenazi (AJ) trio. Because checkpoints differ in total variants called in the sample individual, the line plot (*C*) enables a more direct comparison for HG002 only. The *y*-axis reports the cumulative number of errors found across four distinct versions of DeepVariant (DV, DV-AF, DT, C28). PASS variants in the trio VCF were sorted by genotype quality (GQ) in the offspring, where GQ score decreases as the number of variants increases from *left* to *right* on the *x*-axis. Compared with the human-trained single-sample variant callers, the error curve with checkpoint 28 approaches DT, confirming that our approach successfully recognizes inherited variation. Although DT is a joint caller requiring a minimum of two samples with known pedigree, the bovine-trained checkpoint built with TrioTrain reduces the MIE rate in individual samples, enabling speed improvements.

Given the imperfect bovine truth labels, we used an additional metric, the MIE rate, to assess performance across species, inferring that a lower rate indicates improvement. Although DV was not directly given pedigree information, our training approach is designed to reduce the number of Mendelian discordant genotypes during variant calling. For these analyses, we used the GIAB AJ and Han Chinese (HC) trios, along with three bovine F_1_-hybrids (Methods). Across the remaining eight checkpoints, bovine-trained DV-AF (28) resulted in proportionally fewer Mendelian discordant SNVs in all three F_1_-hybrid bovine trios, even compared with DT (Fig. 5A). Joint calling with DT typically outperforms other versions of DV; however, the wall time to produce results varies significantly, depending on sample coverage and variant content. DV produces results in as little as 6 h when embarrassingly parallelized, whereas DT takes at least two days to achieve the same results. TrioTrain models reduce the MIE rate for individual samples without joint calling, retaining inherited variants in less time.

As with bovine trios, checkpoint 28 achieves the lowest MIE rate in the AJ offspring (HG002). However, the new checkpoint calls about 31,000 fewer indels than the comparable human version (DV-AF), illustrating the trade-off of using our bovine-trained model in human genomes: undercalling indels. Given the curation process of SRS-based bovine truth labels, the observed limitation is expected. Notably, performance with the HC offspring (HG005) behaves inconsistently after retraining with cattle; the MIE rate nearly doubles with the first two phases of TrioTrain (Fig. 5B). However, performance recovers over the entire training period, with the bovine DV-AF checkpoint (28) achieving the same Mendelian discordancy rate as DV while producing 86,000 more variants. Although the existing human-trained models identify more variants in bovine samples, they also introduce more variants that violate Mendelian inheritance expectations. As the number of calls made in the same individual varies between checkpoints, we sorted the AJ trio VCFs by GQ in the offspring (HG002). The resulting MIE calibration curve illustrates that TrioTrain substantially reduces inheritance errors relative to existing human-trained single-sample checkpoints, more closely resembling DT (Fig. 5C). Based on these findings, we selected checkpoint 28 (model.ckpt-282383; see Data access section) as the best-performing DV-AF built with TrioTrain.

During a manual review of MIEs in HG002, we contrast discordant genotypes from the default version (DV) that were concordant with the bovine-trained checkpoint (28). Most MIEs were indels (DV, 87%; 28, 67%), which are difficult to resolve owing to representation differences between individuals. Discordant SNVs predominantly fell outside the benchmark regions for HG002 (DV, ∼94%; 28, ∼91%), which were excluded from manual review. We randomly selected a subset of discordant SNVs with known genotypes for the trio. With these, the human-trained DV typically introduced discordant HomAlts that were labeled as concordant Hets with the bovine-trained DV-AF checkpoint. Relative to HG002's GIAB truth calls, genuine errors with checkpoint 28 occurred because the context provided by SRS alone contradicted the GIAB truth genotypes (Supplemental Figs. S17–S19). The discordant variants produced owing to genotyping errors from DV were typically not made by the new checkpoint 28 (Supplemental Figs. S20–S22). The genuine discordant FPs with DV that were subsequently corrected by our checkpoint were commonly found in HG003 (father)—the only GIAB sample consistently withheld during prior training—indicating that training in bovine samples provided relevant context for genotyping humans. Further review of known de novo variants in the GIAB offspring focused on biallelic Mendelian discordant SNV (Supplemental Table S12). Of the four checkpoints compared (DT, DV, DV-AF, 28), DT produced more TP genotypes, but all expected Mendelian discordancies became concordant. However, sites that remained discordant with the other checkpoints occurred owing to inconsistent genotypes rather than correctly inferring de novo variation. During these analyses, a more recent version of DT (v1.6) introduced a feature designed to preserve de novo variant recall by increasing the importance of known variants during training. Without LRS for other species, this feature could potentially improve genotyping accuracy for the single-sample variant callers given validated de novo variation (Porubsky et al. 2025).

### Recommendations for applying TrioTrain in other species

Our results demonstrate that an optimal variant caller for human genomes may not be appropriate in all mammals. However, using the TrioTrain pipeline for species-specific customization of a DV checkpoint requires substantial upfront investment in maximizing callset quality. Avoiding missteps during truth set curation requires appreciating how every prior step affects the data (Whalen et al. 2022). As such, model builders should identify any limitations with their current genotyping approach in samples representative of the intended cohort. First, compare summary distributions (i.e., depth of coverage, variant density, SNV:indel, Het:HomAlt, Ti:Tv) relative to all prior data used to train a particular DV checkpoint (Supplemental Tables S6, S7) as unexpected deviations may indicate retraining is necessary. Even if high-quality training data or trios are unavailable, we encourage standardized reporting of these variant-calling metrics when using human-based defaults in other species. Comparative data would help justify developing more diverse benchmarking resources and quantify progress over time.

Next, consider the impacts of selecting training samples based on the trios available, focusing on sample quality rather than quantity. Because the multitrio approach shows incremental improvement over a single-trio approach (Supplemental Fig. S16), multiple trios would be ideal but not mandatory. We expect fewer than 14 trios could produce a generalized species-specific model with strategic training sample selection. In domesticated animal species, we recommend using breeds that differ from the reference genome, prioritizing diverse training samples over replicates within the same breed. Our results suggest selecting unrelated trios rather than using shared pedigree, such as half-sibling offspring. Pragmatically, a minimum of two trios would allow one for training, with the second remaining independent for testing. Although single-sample variant calling with the optimal bovine-trained checkpoint (28) requires known population allele frequencies, these data are not mandatory to pursue extending the default DV with TrioTrain.

Before pursuing training, identify potential strategies for maximizing genotyping confidence while avoiding overly strict filtering. Currently, alternative variant-calling methods remain essential to build independent species-specific truth labels despite the burden of optimization. Although the human-trained versions of DV can discover potential novel variants, using these loci as truth variants requires tedious manual inspection. Most novel variants identified by DV within cattle trios were ambiguous, in which truth was not easily identifiable when visualizing aligned short reads in the Integrative Genomics Viewer (IGV) (v2.16) (Robinson et al. 2011). Resolving these difficulties would benefit from long-read sequencing, but unfortunately, these data do not exist for many of the cattle trios from historical data (phases 1 and 2). We acknowledge that reliance on SRS data alone to build bovine truth sets impacts training accuracy with indels and highly repetitive regions, restricting generalization with human genomes. These limitations may require strategically adding the human GIAB trios between species-specific iterations until the characterization of these genomic regions in other species improves.

Fortunately, efforts to improve non-human assembly quality are continuously expanding the number of samples sequenced with multiple platforms, simultaneously creating new sources of training data. However, we recommend estimating the per-base error rate before creating synthetic training data from any assembly. Using synthetic reads during the last bovine training phase increased the SNV MIE rate in all trios compared with the prior phase (Fig. 5A,B). Given the reported quality values (QVs) (Supplemental Table S11), we do not consider synthetic data derived from these three existing bovine assemblies to be a viable source of training data. Regardless of species, prioritizing assembly quality in multiple individuals is essential for increasing the diversity of assembly-based truth labels.

## Discussion

The remaining challenges within genome biology require technologies designed for the continuously expanding diversity of sequencing data. Variant discovery is the first of many hurdles encountered during comparative genomic research, resulting in a trade-off between quality and time. The difficulty of optimizing methods for new species leads many to rely on the defaults intended for the human genome. Studying critically endangered species adds urgency to the decision, as recording population diversity is essential to monitor decline and prioritize intervention. However, the growing prevalence of DL-based methods introduces unknown effects of implicitly trusting a sophisticated model, as illustrated by a previously customized model for the endangered Kākāpō parrots (Guhlin et al. 2023). Prior to retraining, the small population size (fewer than 200) impacted GATK's accuracy, resulting in a higher rate of MIEs compared with DV (v0.9; 3.38% and 1.28%, respectively). However, using DV to create the Kākāpō truth labels leads to circular training that may unintentionally retain bias toward the human genome. The SNV MIE rate reported for Kākāpō is 2.6× higher than our benchmarks with the default DV (v1.4) with the GIAB samples (mean, 0.49%). Despite potential bias from the human genome, the Kākāpō DV callset was curated by altering Mendelian discordant sites in the offspring to match the parental genotypes. There are always limitations during experiment design, particularly in novel or endangered species with inherently small sample sizes, but our research with TrioTrain quantifies their magnitude.

Regardless of the method, relying on defaults intended for human genomes has consequences. Parallel research using the default human genome–trained DV (v1.5) to polish cattle assemblies reported double the SNV error rate (1.06%) relative to our benchmarks with humans (Leonard et al. 2024). For context, prior to removing Mendelian discordant sites, the VQSR-optimized GATK callsets for within-species trios (phases 1 through 3) have a minimum MIE rate of 1.14% (mean, 2.40%, max, 3.92%). The overlapping error rate between VQSR-optimized GATK and human-trained DV illustrates the nuances of method selection in non-human genomics. Although a lack of genomic benchmarks in cattle obstructs optimal method selection, releasing these data (VQSR-optimized UMAGv1) represents the first step toward a GIAB-quality ground truth in bovids and other agricultural species. Although creating a cattle-specific variant benchmark is beyond the scope of this paper, we are in the early stages of developing a GIAB-quality variant benchmark in a new cattle trio, starting with developing cell lines to ensure the long-term value of the resource.

As these resources continue to mature, potential limitations must be readily transparent. For example, previous work using human cohorts illustrated the pathogenic impacts of VQSR owing to the omission of rare and de novo variants (AlDubayan et al. 2020). By excluding all discordant sites from the UMAGv1 trio VCFs, we compromise unknown de novo variation to address systematic errors during variant calling, which has limitations. For example, the predicted genotype for the offspring can be correct yet classified as discordant owing to parental genotyping errors. Systematic errors could lead to a concordant site with incorrect genotypes in all three individuals. Filtering out discordant variants will also exclude some genuine indels represented differently between individuals. However, we emphasize that the UMAGv1 cohort callset exceeds typical variant quality compared with the community standards in animal genomics (1kBulls). Short of producing species-specific GIAB benchmarks, these data serve as a current upper limit of variant quality achievable for any agricultural species. Although referred to as “truth labels,” the genotypes used for training must be considered preliminary. They cannot represent all possible true variants; instead, they are regions we can reliably and accurately genotype in cattle with SRS data. As such, the truth labels exclude highly repetitive and GC-rich regions owing to shorter read lengths and platform-specific limitations. Given these factors, the bovine-trained checkpoint is more conservative than the human-trained default in the GIAB samples. Unfortunately, many non-human species lack extensive characterization of complex or repetitive genome regions; without these resources, customization of DV in non-human species will continue to struggle with these areas.

Despite these caveats, our bovine-trained model reduces the Mendelian error rate in bovine trios by an order of magnitude compared with the default DV checkpoint, confirming that TrioTrain enables DV to select model weights to identify inherited variants. We release this initial version to bridge the divide between GATK and DV for cattle genomics while minimizing the potential of making incorrect biological inferences in cattle. For example, previous work found that DV increased the proportion of HomAlt genotypes, which increased the amount of high-impact functional consequences predicted in cattle (Lloret-Villas et al. 2023). Until more comprehensive cattle-specific variant benchmarks compel retraining, the bovine-trained checkpoint (28) reduces implementation barriers compared with GATK while improving upon the current standards (1kBulls). The established tools, resources, and population-scale SRS data for cattle often guide research strategies in other agricultural species, so the initial success with bovine genomes provides encouraging evidence about replicating our work. Although we anticipate that checkpoint 28 will produce high-confidence callsets of common variation in other domesticated animals, future work must evaluate how well our checkpoint generalizes in new species relative to those exclusively trained with human genomes and evaluate the impact of warm-starting with a revised human DV-AF model (v1.4+) that combines both channels (The 1000 Genomes Project Consortium 2010; Chen et al. 2023). For now, those working in other species must remain skeptical of any downstream consequences and pursue validation. We caution against inferring that the performance of any variant caller will replicate consistently in all other species, including the model accompanying this manuscript.

Prioritizing human genomes has enabled many advances in specialized DL methods, but comparative genomic research depends on high-quality variant callsets from multiple species. Previous work focused on building species-specific models that require continuous retraining every time the human model is improved. Developing separate models for each species is cost-prohibitive across the exponentially growing species list with sequencing data. However, TrioTrain establishes an iterative, trio-based training approach for customizing single-sample variant-calling checkpoints to any diploid species with the necessary input data. The pipeline focuses on reducing the computational burdens required to customize DV. With TrioTrain, we provide the first DV model trained in multiple mammalian species for comparative genomic research.

Our results demonstrate that historical sequencing data can adjust DV to the distributional shifts that arise when aligning multiple related species against the same reference genome. Given that training with a bison trio improves training performance in homozygous alternate variants, our work suggests previously unknown benefits of training within genera. Future research must determine if the human-trained model would benefit from retraining with other hominid species. Exploring DV's behavior during training with high-quality data from more organisms may reveal previously hidden unknowns in genomics. For example, learning behavior across more species could reveal new insight based on the phylogenic boundaries that achieve optimal generalization. With similar high-quality variant benchmarks, DV could feasibly become a “golden model” for comparative genomics. Compared with the human-specific DV, a theoretical species-agnostic variant caller would be generalizable across broader phylogenic classifications but could also struggle with rare variant discovery in humans. In building TrioTrain, we provide a tool for exploring training across species to illustrate how the behavior of DL models enables investigating the persistent challenges in genomics. Future innovation requires looking beyond the human genome; our work provides an initial template for creating diverse, pedigree-based benchmarking resources for other species.

## Methods

### Raw sequencing data

The University of Missouri Animal Genomics (UMAG) group maintains a repository containing the publicly available genomes for several agricultural species, including bovines. As new samples are published, the paired-end, short-read Illumina WGS data are obtained from the NCBI's Sequence Read Archive (SRA; https://www.ncbi.nlm.nih.gov/sra/). We identify bovine samples based on SRA metadata for taxon ID for multiple species (Supplemental Note S3). Where available, we also collect reported sex, breed, and pedigree information. Samples are aggregated based on BioSample ID and assigned an internal, integer-based UMAG lab ID unique to a single animal. We refer to the bovine cohort as the UMAGv1 cohort (Supplemental Note S4). At the time of analysis, the cohort included 5612 individuals with complete metadata, spanning modern and ancient genomes from related bovine species: *Bos taurus taurus* (Taurus cattle), *Bos taurus indicus* (indicine cattle), *Bison bison* (American bison), *Bos mutus* (yak), *Bos grunniens* (yak), *Bos frontalis* (gayal), *Bos javanicus* (banteng), and *Bos gaurus* (gaur).

### Whole-genome sequence alignment

After downloading the raw FASTQ files from SRA, the data are preprocessed (Supplemental Notes S5–S8). Briefly, low-quality bases and adapters were trimmed from the reads with Trimmomatic (v0.38 or v0.39) (Bolger et al. 2014) and subsequently aligned to the reference genome, ARS-UCD1.2 with Btau5.0.1 Y Chromosome (Rosen et al. 2020) using either BWA-MEM (v0.7.17) (Li 2013) or BWA-MEM2 (2.2.1) (Md et al. 2019). The resulting sequence alignment map (SAM) output is sorted, indexed, and compressed into binary alignment map (BAM) files. Duplicate reads are marked with Picard MarkDuplicates (v2.18.19 or v2.26.10) (https://broadinstitute.github.io/picard). Our internal pipeline automatically performs indel realignment and base quality score recalibration (BQSR) and produces callable region BED files (see Data access section) (Supplemental Notes S9, S10). Note that DV does not require the realignment and recalibration of raw data. Coverage and other summary metrics were calculated using SAMtools (v1.9) (Supplemental Note S11; Danecek et al. 2021). The average coverage for the entire cohort before sample QC was 12.77× (minimum, 0.07×; maximum, 182.78×).

### Initial variant calling

The processed sequence data were used for germline variant calling for single-nucleotide variants (SNVs) and short inserts and deletions (indels; <50 bp). We refer to the resulting VCF as the UMAGv1 callset. Genotypes for the UMAGv1 cohort were prepared for all 5612 bovine samples, including outgroup species, using an internal workflow that expands upon the established 1kBulls GATK FASTQ to GVCF guidelines (Hayes and Daetwyler 2019). The 1kBulls protocol uses GATK HaplotypeCaller (v3.8) (Poplin et al. 2018b) to generate single-sample callsets, which are merged before joint genotyping (Supplemental Notes S12, S13). However, using bovine trios, our internal workflow is further optimized (Supplemental Figs. S23–S32; Supplemental Notes S14–S17). In brief, significant improvement was made during VQSR as parameters were extensively tuned from defaults intended for human genomes.

### Sample selection

A subset of individuals from the final UMAGv1 callset were selected as the high-quality bovine genotypes used for retraining DV. We used mean sequencing coverage to select candidate samples from the UMAGv1 cohort with moderate sequencing depth by excluding samples with a mean coverage of less than 10×. Related samples were identified using reported pedigree metadata. Candidates for training samples were constrained to parent–offspring trios by excluding samples missing pedigree data or samples with sequencing data for only one parent. We identified 14 unique trios, resulting in 40 unique variant data sets with a mean coverage of 25.99× (minimum, 10.66×; maximum, 50.83×). These 14 trios consist of sires (N = 12), dams (N = 14), and their offspring (N = 14) across multiple cattle breeds and crossbreeds (Supplemental Note S18; Supplemental Table S4). In addition to the trios, we identified other genomes we could use to represent the final data given to the DL model. These genomes were used to monitor performance changes over the iterative retraining process and assess the generalizability of each model created with TrioTrain. We selected 13 samples from multiple cattle breeds with a mean coverage of 29.63× (minimum, 13.54×; maximum, 60.00×). These testing genomes included representatives from 15 cattle breeds, with eight additional breeds not used during training (Supplemental Note S19; Supplemental Table S5).

### Bovine truth set curation

From the UMAGv1 cohort data set, we extracted variants for all three samples within a trio into family VCF files (Supplemental Note S20). Briefly, within each family VCF, we removed any Mendelian discordant genotypes. For example, at one locus, if one parent is A/B and the other parent is B/B, but the child has a genotype A/A, that position was dropped from all three individuals. We then split the family VCF into single-sample VCFs. Because of a lack of parental sequencing data, Mendelian discordant variants could not be removed for the nontrio samples used during testing. The single-sample VCFs used for training, tuning, and testing were then filtered to retain PASS filter sites only, for which PASS was set to tranche level 99.7. Lastly, to meet the specifications required by DV for labels, the final VCFs exclude all homozygous reference genotypes.

### Synthetic bovine offspring

Synthetic diploid (SynDip) approaches have been proposed as alternatives to GIAB-quality benchmarking data (Li et al. 2018). These efforts inspired us to consider if ongoing efforts to produce multiple high-quality bovine assemblies could be used to generate high-quality synthetic benchmark variants for cattle (Zhou et al. 2022; Smith et al. 2023). To compare both strategies of truth label curation in non-human species, we used the haploid parental assemblies to create synthetic reads for the offspring in all three trios (Supplemental Note S21). Briefly, we created synthetic reads from each parental assembly using NEAT (v3.2) (Stephens et al. 2016), introducing no sequencing error, and then merged these synthetic reads, resulting in three more offspring replicates. As such, each new trio consists of the parents’ original SRS data, but the offspring is substituted with the synthetic SRS. These F_1_-hybrid offspring have unique identifiers, separate truth labels, and a different noise signature relative to the nonsynthetic samples. The three unique F_1_-hybrid offspring and their respective synthetic replicates (N = 6) have a mean coverage of 26.7× (minimum coverage, 14.46×; maximum coverage, 50.01×).

### Shuffling labeled examples

First, TrioTrain (v0.8) calculates the number of regions based on the variants within the Truth VCF input for a genome (Supplemental Notes S23, S24). These regions’ BED files are passed to DV through the ‐‐regions flag. We designed region shuffling to batch the complete genome to create representative samples of the total examples (Supplemental Fig. S34). These region-specific steps are submitted across compute nodes as independent SLURM jobs. Crucially, a region-specific tfrecord file contains examples produced from across the genome. Next, within-region shuffling is performed, and then, all tfrecord “shards” are concatenated into a single file. Finally, the resulting labeled and shuffled examples across all regions are fed to the DV model in a randomized order. Our region shuffling process successfully enables the DV Beam shuffling pipeline on a SLURM-based HPC cluster. Rather than rely on a different resource manager, our pipeline creates embarrassingly parallel shuffling jobs. Our design avoids extensive system-administrator support to create a Spark cluster on top of the existing SLURM-controlled cluster. Instead, our approach uses Beam's Direct Runner to shuffle examples. Direct Runner is constrained to the local memory within a single compute node and is typically applied for pipeline debugging. However, SLURM effectively controls multiple independent Direct Runner jobs simultaneously.

### Initializing model weights

Because of the wide variability in coverage levels within the complete UMAGv1 cohort, we opted to add the allele frequency channel during our retraining. Before retraining begins, an existing model checkpoint from human genomes is used to initialize model weights, or “warm-starting.” At the time of analysis, the only available WGS-specific model with DV (v1.4) added a new channel for insert size compared to previous model versions (v1.1). Because the additional channel was not yet incorporated into the existing human-trained DV-AF, we used the new DV checkpoint as the training starting point. TrioTrain enabled building new checkpoints that encode for both insert size and population-level allele frequencies (Supplemental Note S25). We created the PopVCF using the UMAGv1 callset by dropping genotypes and retaining only variant allele frequencies for the joint genotypes across the entire cohort (N = 5612).

### Retraining

Running TrioTrain requires organizing metadata and file paths into a single metadata CSV file (Supplemental Note S22). The number of examples created and used varies for each parent (Supplemental Table S6). For retraining the network, we used a learning rate of 0.005 and a batch size of 32 and defined the training period as one epoch that spans all labeled candidate variants, known as examples, for a training genome. Performance metrics in the offspring (Supplemental Table S7) from the selected best checkpoint for all 34 iterations were stratified by variant type and genotype class (Supplemental Table S8).

### Summarizing performance

We obtained the 1000 Genomes Project's population VCF and the GRCh38 reference genome from Google Genomics. We then acquired the GIAB benchmarking files (v4.2.1), corresponding sample-specific callsets, and callable region files from the National Institute for Standards and Technology (NIST) FTP site (Supplemental Note S26). We used two trios: (1) the Ashkenazi trio with HG002_NA24385_son, HG003_NA24149_father, and HG004_NA24143_mother and (2) the HC trio with HG005_NA24631_son, HG006_NA24694_father, and HG007_NA24695_mother. Benchmarking parameters were identical to those used to calculate bovine performance (Supplemental Note S27). The only difference is switching to the human reference genome, truth labels, callable regions, and the corresponding model checkpoints. The resulting VCFs were then passed to hap.py (v0.3.12) using the appropriate reference genome and truth file. Performance metrics, such as recall, precision, and F1 score, were stratified by variant type (SNV, indel) and genotype class (HomRef, Het, HomAlt). Raw metric values were collected and plotted using a custom Python (v3.12) script. Summary metrics from a subset of checkpoints are available in bovine trios (Supplemental Table S9) and human GIAB samples (Supplemental Table S10).

### Validating performance using Mendelian discordancy

Without a benchmarking callset for bovine genomes, we cannot directly compare the F1 score across species. However, “most methods used to identify and genotype genetic variants from [high-throughput] sequencing data ignore the relationships between samples, resulting in significant Mendelian errors, false positives, and negatives” (Cleary et al. 2014). Therefore, we used the MIE rate to infer relative performance changes between species. We used Real Time Genomics (RTG) software, rtg-tools mendelian (v3.12.1), to calculate the MIE rate (Cleary et al. 2015). For this analysis, we again used the same models from GIAB benchmarking: five terminal checkpoints from the TrioTrain phases and the three existing DV models (v1.4: default WGS, WGS.AF, DT). However, we used human and bovine trios: specifically, the two GIAB trios and the three F_1_-hybrid trios described previously. Each sample was rerun using DV separately, except with DT, a joint caller; all models created per-sample VCFs as output (Supplemental Note S28).

### Data sets

A subset of the data for the two most recent genotyping runs produced by the 1kBulls project is available at the European Nucleotide Archive (ENA; https://www.ebi.ac.uk/ena/browser/home) under accession numbers PRJEB42783 and PRJEB56689. For bovine samples, all raw sequencing data used in this study were obtained from the NCBI Sequence Read Archive (SRA; https://www.ncbi.nlm.nih.gov/sra) under accession numbers provided in the Supplemental Material (for training and evaluation, see Supplemental Table S4; for testing, see Supplemental Table S5). The Supplemental Material for human GIAB benchmarking samples also provides details about how the files were obtained for this study (Supplemental Note S26).

### Software availability

Copies of the source code for the TrioTrain pipeline v0.8, as well as custom scripts used in this study, are provided as Supplemental Code. Further details about the source code, installation instructions, documentation, and tutorials are available at GitHub (https://github.com/jkalleberg/DV-TrioTrain/releases/tag/v0.8). Source code used for sequence data processing (Supplemental Notes S4–S11), using GATK (Supplemental Notes S12–S17), and producing input files for TrioTrain (Supplemental Note S20) is provided as Supplemental Material.

## Data access

The variant data generated in this study have been submitted to the European Nucleotide Archive (ENA; https://www.ebi.ac.uk/ena/browser/home) under accession number PRJEB86883. Additional data including pedigree and breed labels (metadata.csv), the per-sample callable region files (CallableRegions.tar.gz), UMAGv1 cohort population allele frequency file (UMAG1.POP.FREQ.vcf.gz), reference genome file (ReferenceGenome.tar.gz), and the final selected TrioTrain checkpoint (28; ModelCheckpoint.tar.gz) can be found at Zenodo (https://doi.org/10.5281/zenodo.15482484).

## Supplemental Material

Supplement 1

Supplement 2

Supplement 3
