# Supplementary figures and images for: Overcoming limitations to customize DeepVariant for domesticated animals with TrioTrain
Source: Genome Res. 2025 Aug;35(8):1859–74. doi: 10.1101/gr.279542.124 (PMC12315867; doi:10.1101/gr.279542.124)

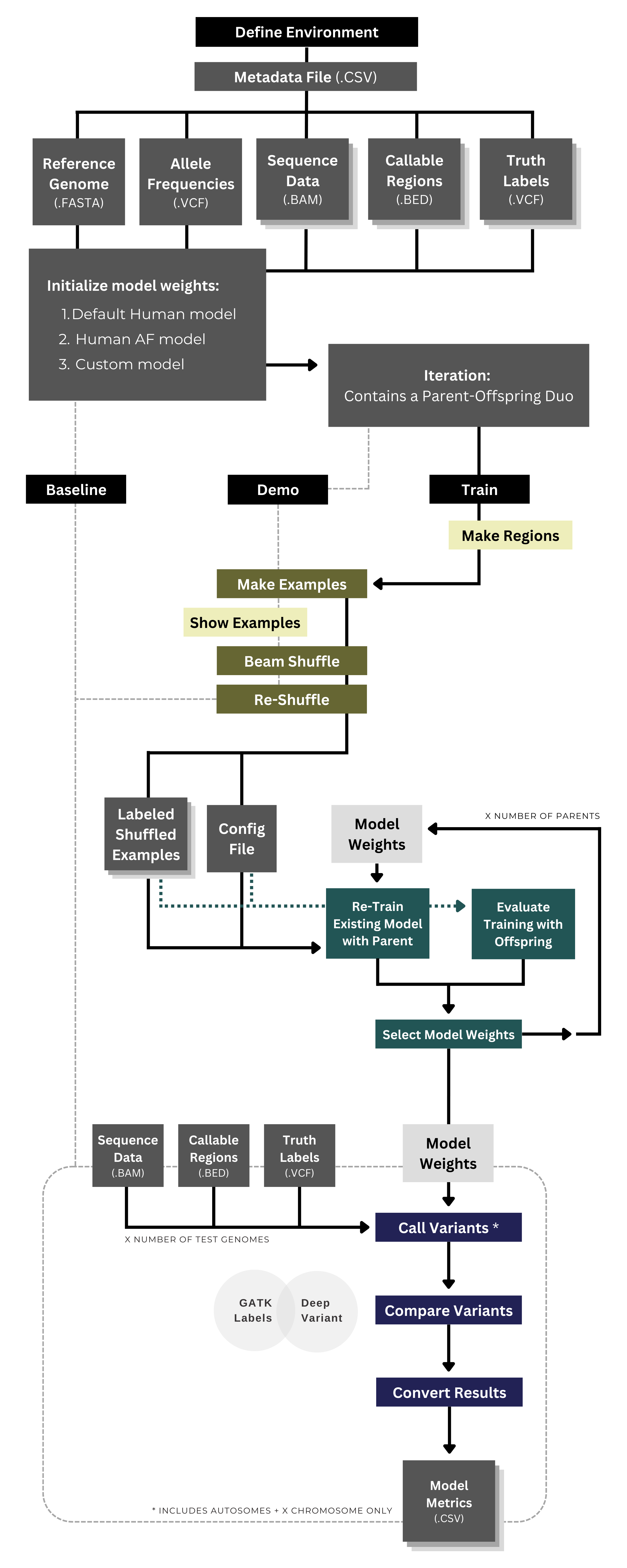

Supplement: Supplement 1 [file Supplemental_Code.tar.gz › DV-TrioTrain-0.8/docs/img/Pipeline_v0.8.png]

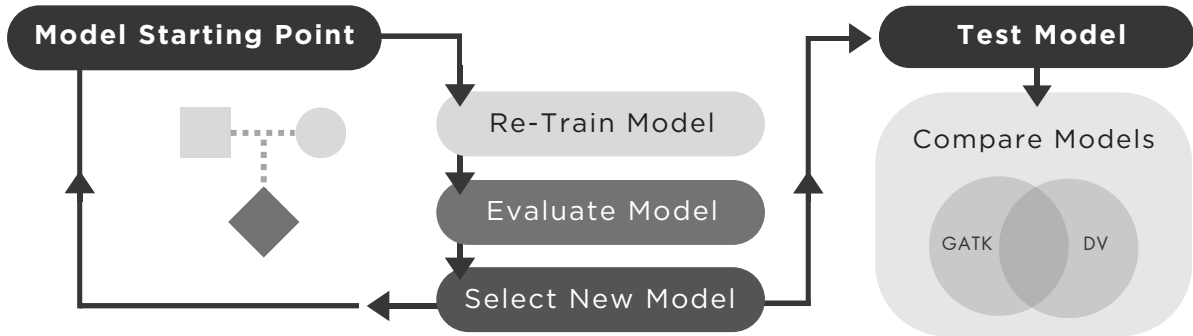

Supplement: Supplement 1 [file Supplemental_Code.tar.gz › DV-TrioTrain-0.8/docs/img/Workflow_Sm_Horizontal.pdf]

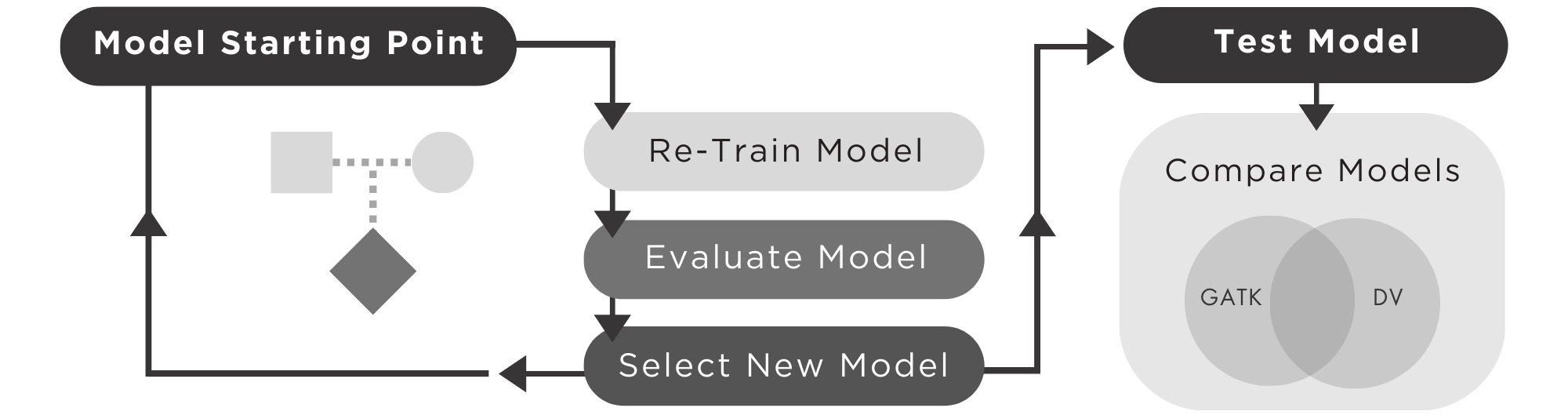

Supplement: Supplement 1 [file Supplemental_Code.tar.gz › DV-TrioTrain-0.8/docs/img/Workflow_Sm_Horizontal.png]
