# Supplementary material for: Overcoming limitations to customize DeepVariant for domesticated animals with TrioTrain
Source: Genome Res. 2025 Aug;35(8):1859–74. doi: 10.1101/gr.279542.124 (PMC12315867; doi:10.1101/gr.279542.124)
Supplement: Supplement 3 [file Supplemental_Materials.pdf]

Supplementary Materials: Overcoming limitations to customize DeepVariant for domesticated animals with TrioTrain

Jenna Kalleberg[1], Jacob Rissman[1], Robert D. Schnabel[1,2]
[1] University of Missouri, Division of Animal Sciences, Columbia, MO, 65201 USA
[2] University of Missouri, Genetics Area Program, Columbia, MO, 65201 USA
Corresponding author: schnabelr@missouri.edu

SUPPLEMENTAL RESULTS **4**
Figure S1. Preliminary comparison of GATK and DV 4
Note S1. Trouble-shooting during training. 4
Figure S2. Loss plots reveal truth label quality issues 5
Note S2. Evaluating concordance across 1000 Bulls callsets. 6
Figure S3. Bovine truth label contents influence training performance 7
Figure S4. Using Hereford cattle genomes to reveal DV's knowledge gaps. 8
Figure S5. Using bison genomes to reveal DV's knowledge gaps. 9
Figure S6. Confirming TrioTrain checkpoints will generalize with cattle genomes. 10
Figure S7. Generalization of the successively built TrioTrain checkpoints with independent bovine genomes trios. 11
Figure S8. Visualizing heterozygous SNVs identified by the human-trained DeepVariant within a homopolymer region in a cattle genome. 12
Figure S9. Visualizing a heterozygous SNV identified by the human-trained DeepVariant within a known LINE in a cattle genome. 14
Figure S10. Visualizing an ambiguous homozygous SNV identified the human-trained DeepVariant in a cattle genome. 16
Figure S11. Visualizing genuine errors from the human-trained DeepVariant corrected after extending with TrioTrain. 18
Figure S12. Visualizing genuine errors from the human-trained DeepVariant corrected after extending with TrioTrain. 19
Figure S13. Visualizing genuine errors from the human-trained DeepVariant corrected after extending with TrioTrain. 20
Figure S14. Visualizing genuine errors from the human-trained DeepVariant corrected after extending with TrioTrain. 21
Figure S15. Comparing checkpoint generalization across species. 22
Figure S16. Comparing precision-recall curves in 10 different checkpoints. 23
Figure S17. Discordant SNV found in a GIAB trio at Chr22:20689462 24
Figure S18. Discordant SNV found in a GIAB trio at Chr22:20690349 24
Figure S19. Discordant SNV found in a GIAB trio at Chr16:15276483 25
Figure S20. Discordant SNV found in a GIAB trio at Chr3:3966790 25
Figure S21. Discordant SNV found in a GIAB trio at Chr3:6768039 26
Figure S22. Discordant SNV found in a GIAB trio at Chr4:60218226 26
SUPPLEMENTAL METHODS **27**
Note S3. Public Samples from SRA 27
Note S4. Sequence Data Processing 27
Note S5. Sequence Quality Control 27

Note S6. Bovine Reference Genome 28
Note S7. Genome Alignment 28
Note S8. Sort/Merge/Mark Duplicates 28
Note S9. Indel Realignment 29
Note S10. Base Quality Score Recalibration (BQSR) 29
Note S11. Summary Metrics 31
Note S12. HaplotypeCaller & CallableRegions 31
Note S13. Joint Genotype Calling 32
Note S14. Variant Quality Score Recalibration (VQSR) 32
Figure S23. VQSR negative model 35
Figure S24. Tranche plot for the optimal SNP model parameters. 35
Figure S25. Tranche sensitivity plot for the optimal SNP model parameters. 36
Note S15. VQSR Mendelian Error Evaluation 36
Figure S26. Tranche-specific Mendelian error rate for SNP variants. 37
Figure S27. Cumulative Mendelian error rate for SNP variants. 37
Figure S28. Tranche-specific Mendelian error rate for indel variants. 38
Figure S29. Cumulative Mendelian error rate for indel variants. 38
Figure S30. Comparing VQSR approaches. 39
Note S16. ApplyVQSR 40
Note S17. Initial Quality Control (QC) 40
Figure S31. Initial QC of samples demonstrating the impact of genome coverage 41
Figure S32. Initial QC of samples with axes truncated. 41
Note S18. Training Sample Criteria and Phases. 42
Note S19. Testing genomes remain independent. 43
Note S20. Creating truth sets for DeepVariant. 43
Note S21. Creating synthetic diploid reads. 44
Figure S33. Workflow diagram for the TrioTrain pipeline. 46
Note S21. Required Inputs for TrioTrain 47
Note S22. Implementing training on a SLURM-based cluster. 47
Figure S34. Region shuffling approach. 48
Note S23. Defining regions for shuffling. 49
Note S24. Checkpoints used for warm-starting training 49
Note S25. Supplemental Data and Code 49
Note S26. Evaluating performance in bovine genomes. 51
Note S27. Calculating Mendelian Inheritance Errors 53

SUPPLEMENTAL RESULTS

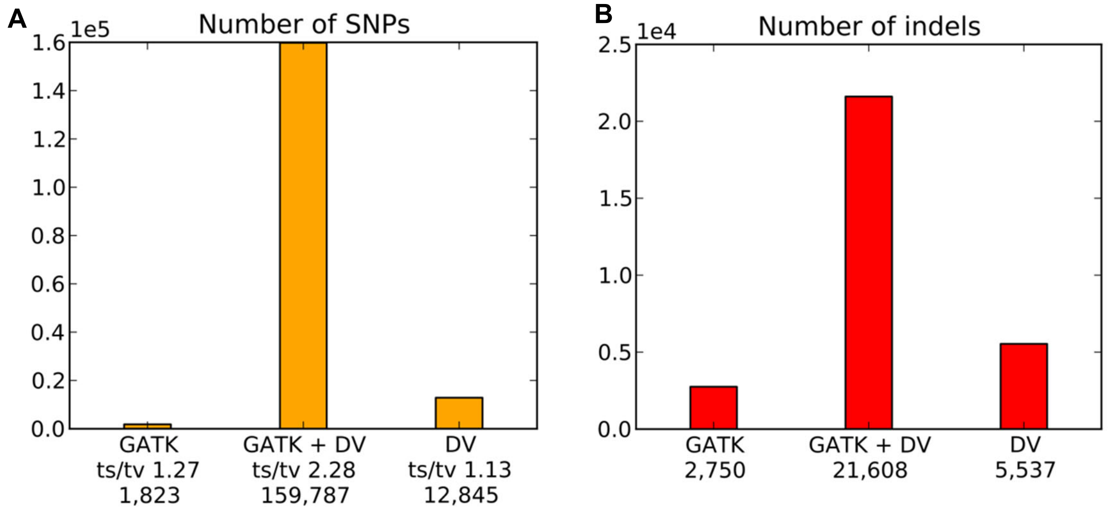

Figure S1. Preliminary comparison of GATK and DV

Before pursuing re-training, we used a single bovine genome (SRA BioSample: SAMN1094070, mean coverage 17.95x) to compare GATK (v3.8) and DeepVariant (v1.0). The y-axis represents raw counts with (**A**) more total Single Nucleotide Polymorphisms (SNPs) than (**B**) Insertion-Deletions (indels). The middle bar contains the overlapping variants between both methods, while the other two outer bars contain unique variants. The lower SNP Transition to Transversion (ts/tv) ratio with DV indicated that the 1kBulls Run8 callset workflow identified more high-quality novel variation than DeepVariant (v1.0). These findings were produced before developing the VQSR-optimized GATK-derived UMAGv1 callset, which was subsequently used for training labels. Both software versions compared here are considered depreciated; however, these results provide context for our decision to pursue re-training.

Note S1. Trouble-shooting during training.

We initially pursued re-training of DeepVariant using the 1kBulls Run8 callset. However, the resulting loss plots indicated overfitting and potentially noisy labels. We concluded that these data were not sufficient to use as training labels. For example, we observed separation between the two loss curves, the optimal checkpoints occurred early in the epoch, and training performance worsened despite filtering to increase truth label sensitivity (**Figure S2**). Additionally, training halted prematurely by using only 6 to 18 percent of labeled examples. Contrary to expectations, more data did not improve learning. These plots revealed issues during truth label filtering, where the order-of-operations logic was not applied consistently between the truth VCF and the PopVCF. We resolved these issues before switching to the UMAGv1 callset.

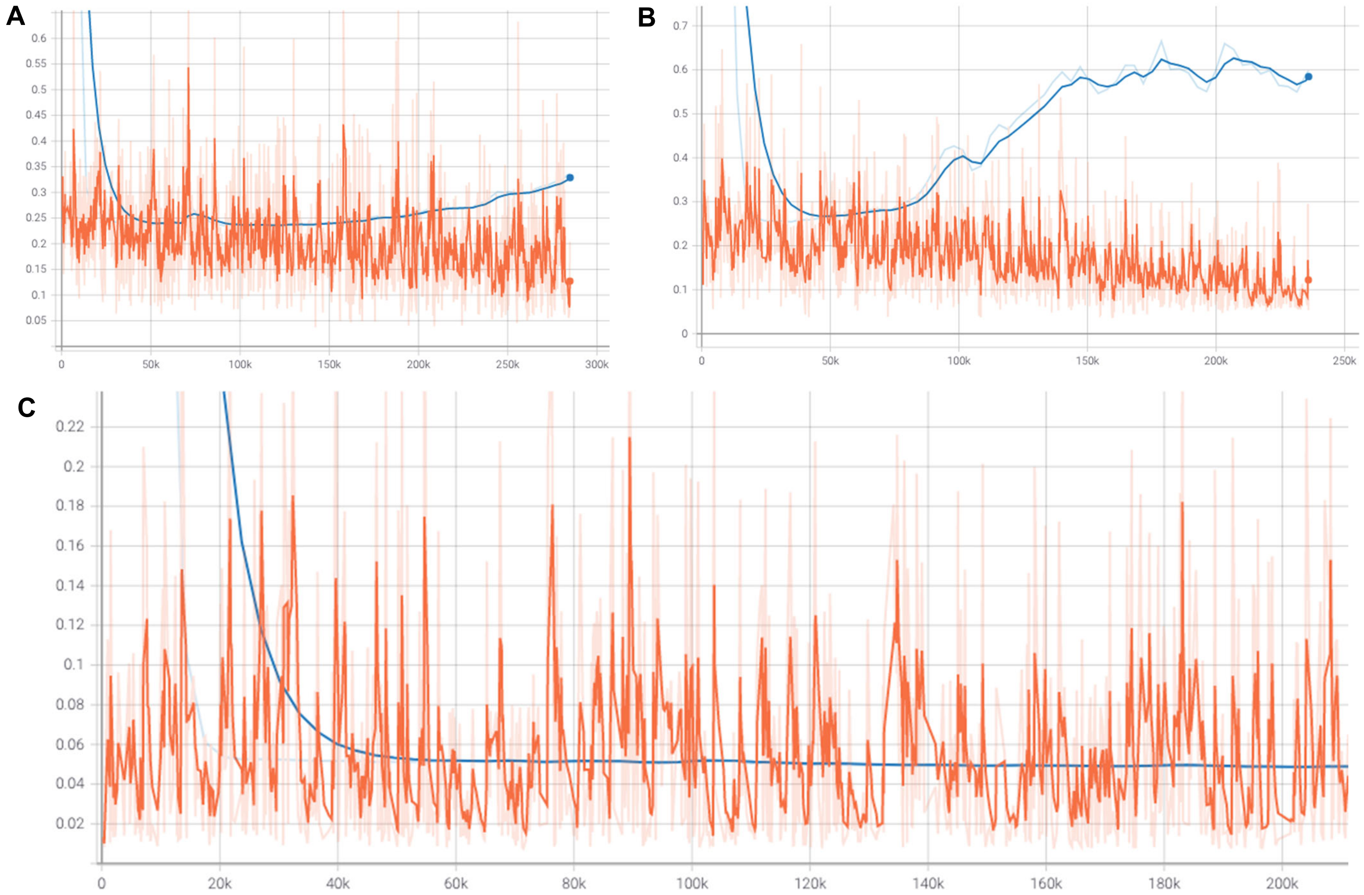

Figure S2. Loss plots reveal truth label quality issues.

The y-axis presents model loss, or prediction error over the training period, across the number of completed batches of labeled examples, known as steps (x-axis). Each line represents a unique genome (smoothed darker lines), where orange is the parent (training), and blue is the offspring (evaluation). Each figure represents a single training iteration with a bovine duo. Both (**A**) and (**B**) were produced during initial training with 1kBulls Run8, while (**C**) demonstrates corrected filtering with the UMAGv1 callset. Training covers all labeled examples for the parent, where each step uses 32 labeled examples (batch size). In general, evaluation loss approaching 0 indicates successful learning. **A.** Model loss during a 1kBulls Run8 training iteration with truth labels from PASS variants (tranche = 90). **B.** A second 1kBulls training iteration, where the only difference is filtering to exclude genotypes with GQ<30 for the truth VCF. In both (**A**) and (**B**), the separation between training (orange) and tuning (blue) indicates overfitting. Comparing A to B, training halted prematurely because of severe overfitting due to inconsistency when filtering truth labels. Without filtering, the optimal checkpoint occurred later in the training period (proportion of examples covered: A = 33.5%, B = 7.4%). We examined the quality of our truth labels since more examples should have improved learning. We discovered that filters for the PopVCF mismatched those applied to the truth VCF, causing different minimum sensitivity thresholds. **C.** A contrasting example of a training iteration using the UMAGv1 callset. The loss for the best checkpoint drops by an order of magnitude with improved truth genotypes (1kBulls Run8 = 0.23469; UMAGv1 = 0.01514). Additionally, we demonstrate that prior issues were corrected with consistent filters. Notice the lack of separation between training (orange) and tuning (blue) loss lines as more labeled examples are given to the model. The optimal checkpoint occurs much later (examples covered = 76.6%).

Note S2. Evaluating concordance across 1000 Bulls callsets.

The 1000 Bulls Project has been the flagship consortium effort in animal agriculture to aggregate genome sequence data from across the globe, standardize data processing, and distribute a final variant call set to participants (Hayes and Daetwyler, 2019). Starting with Run7 in 2019, the data processing pipeline was rewritten to transition to the Broad Institute's Genome Analysis Tool Kit (GATK**,** McKenna et al., 2010) and the new ARS-UCD1.2 reference (Rosen et al., 2020). Since then, there have been three genotyping runs: Run7 in 2019 (N=3818), Run8 in 2020 (N=4931), and Run9 in 2021 (N=6191). We identified samples included repeatedly in all three genotyping runs (N=3659, mean coverage = 13.7x). We developed a pipeline to extract variants that PASS VQSR filtering for each sample to determine the consistency of variant calls over time. Then, we performed a pairwise comparison between runs by providing hap.py with the two VCFs (Krusche et al., 2019). However, without an established 'ground truth' for cattle, we can only measure variability between runs using metrics as quantities. We expected the genotypes to be constant across 1kBulls runs for a sample (F1 score > 0.990). Instead, our results indicate significantly lower recall and precision within the 1000 Bulls data than expected (**Table S2**). The variability in genotypes is due to differences in analysis parameters between runs and the inclusion of additional individuals, among other factors.

During this analysis, we did not exclude challenging or repetitive regions since these are not characterized to the same extent as humans. The comparisons that were performed analyzed PASS variants from each run version. The premise is that, regardless of the genome region, genotype calls for a given sample would be expected to be concordant between separate processing runs. Most users assume that PASS variants for a given sample would not change over time. This analysis illustrated that this is not a safe assumption. Determining if a particular 1kBulls genotyping run is correct is irrelevant. Instead, for this analysis, the fact that the PASS genotypes for the same individual are different between each genotyping run and the magnitude of the change is more relevant.

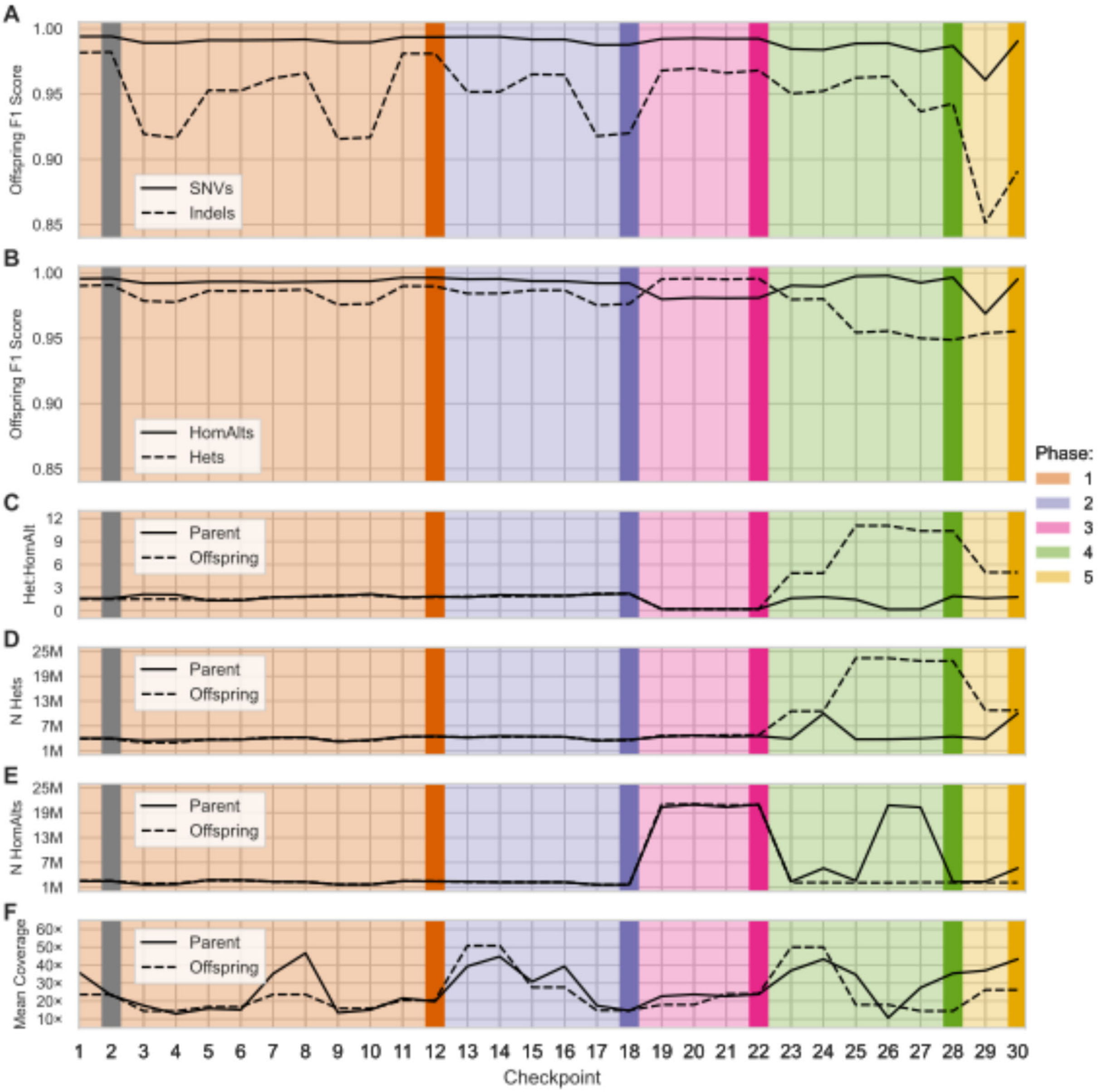

Figure S3. Bovine truth label contents influence training performance.
Performance during training (**A, B**) depends on class imbalance within the truth labels, as reflected by the Het:HomAlt ratio (**C**). Monitoring training metrics over multiple iterations reveals how DeepVariant behaves when challenged with new contexts, such as a sudden shift in offspring Het majority class (**D**) or HomAlt minority class (**E**). Based on these results, we advise comparing the expected Het:HomAlt ratio for a particular species, if known, against the human-genome-based assumptions of DV (**Table S6**). Quantifying distributional changes can provide evidence that further training may be warranted. However, these heterozygosity priors are one of several interacting summary metrics relevant to DV during variant calling. For example, heterozygous variants can become indistinguishable from sequencing errors with lower mean coverage. However, incorporating the allele frequency channel into DeepVariant with a bovine PopVCF (UMAGv1) minimizes the impact of using low-coverage samples during training (**F**).

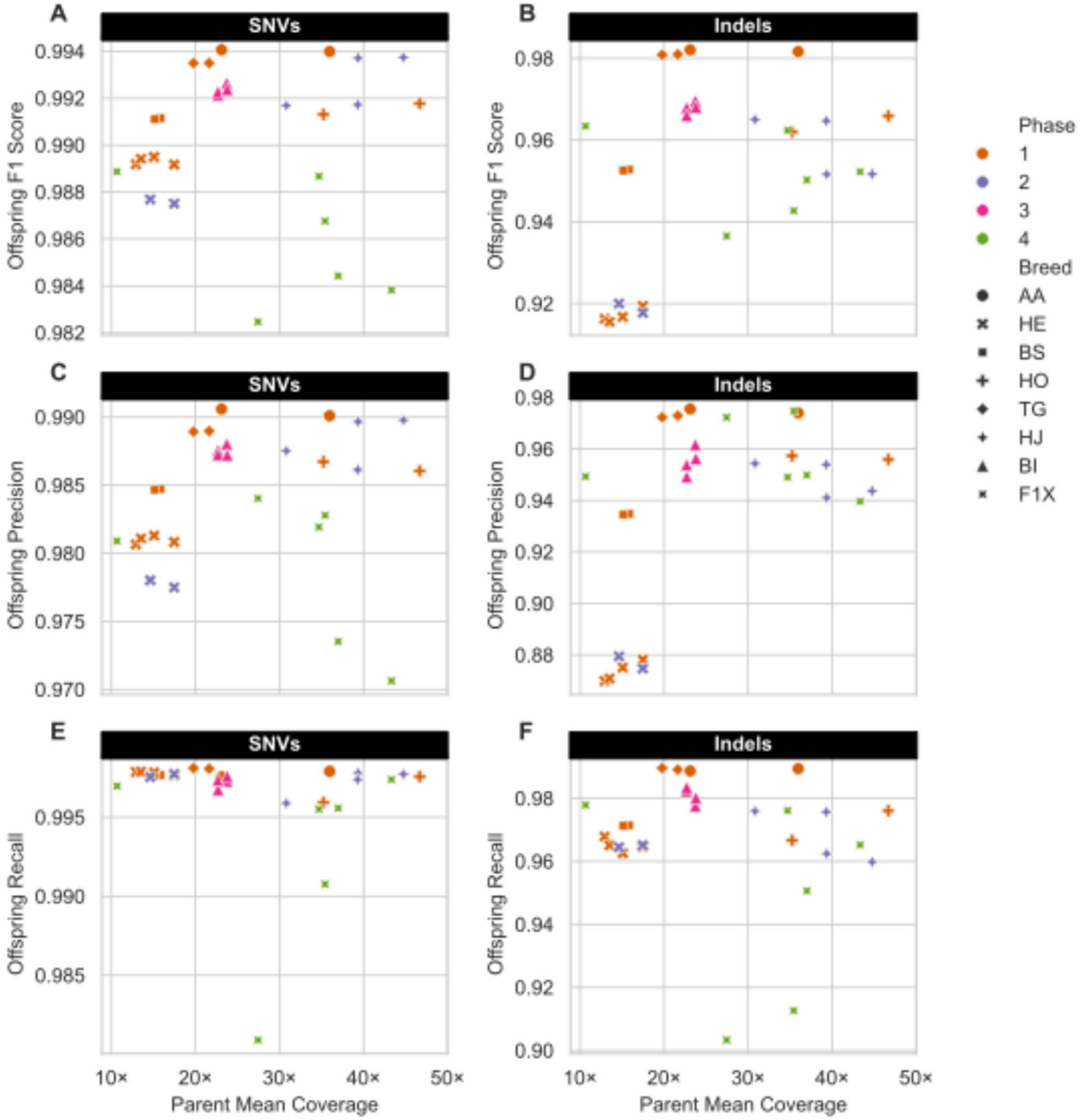

Figure S4. Using Hereford cattle genomes to reveal DV's knowledge gaps.
The x-axis illustrates the variable sequencing coverage across parent genomes used during training. Note that the y-axes are scaled differently and represent three metrics (top: F1 score, middle: precision, bottom: recall), stratified by variant type (left: SNVs, right: indels), reported by DV during model evaluation. Each point represents a new, bovine-trained DV-AF checkpoint created with TrioTrain, where the optimal stopping point maximizes the offspring's F1 score. Point color designates checkpoints produced over four training phases, while marker shape represents a parent's breed or outgroup. By providing the UMAGv1 population allele frequencies during training (channel #8), we aimed to minimize the consequences of variant calling in samples with low coverage. These results illustrate that using parents with variable coverage during training has minimal impact on prediction error in the offspring, as intended. **D.** A notable cluster of checkpoints with low precision (more False Positives) occurs with parents of a single breed matching the cattle reference (HE=Hereford). In contrast, iterations with other cattle breeds have consistent indel precision, regardless of coverage. These findings illustrate the limitations of focusing on a single attribute, such as coverage, when interpreting results from extending DeepVariant with TrioTrain.

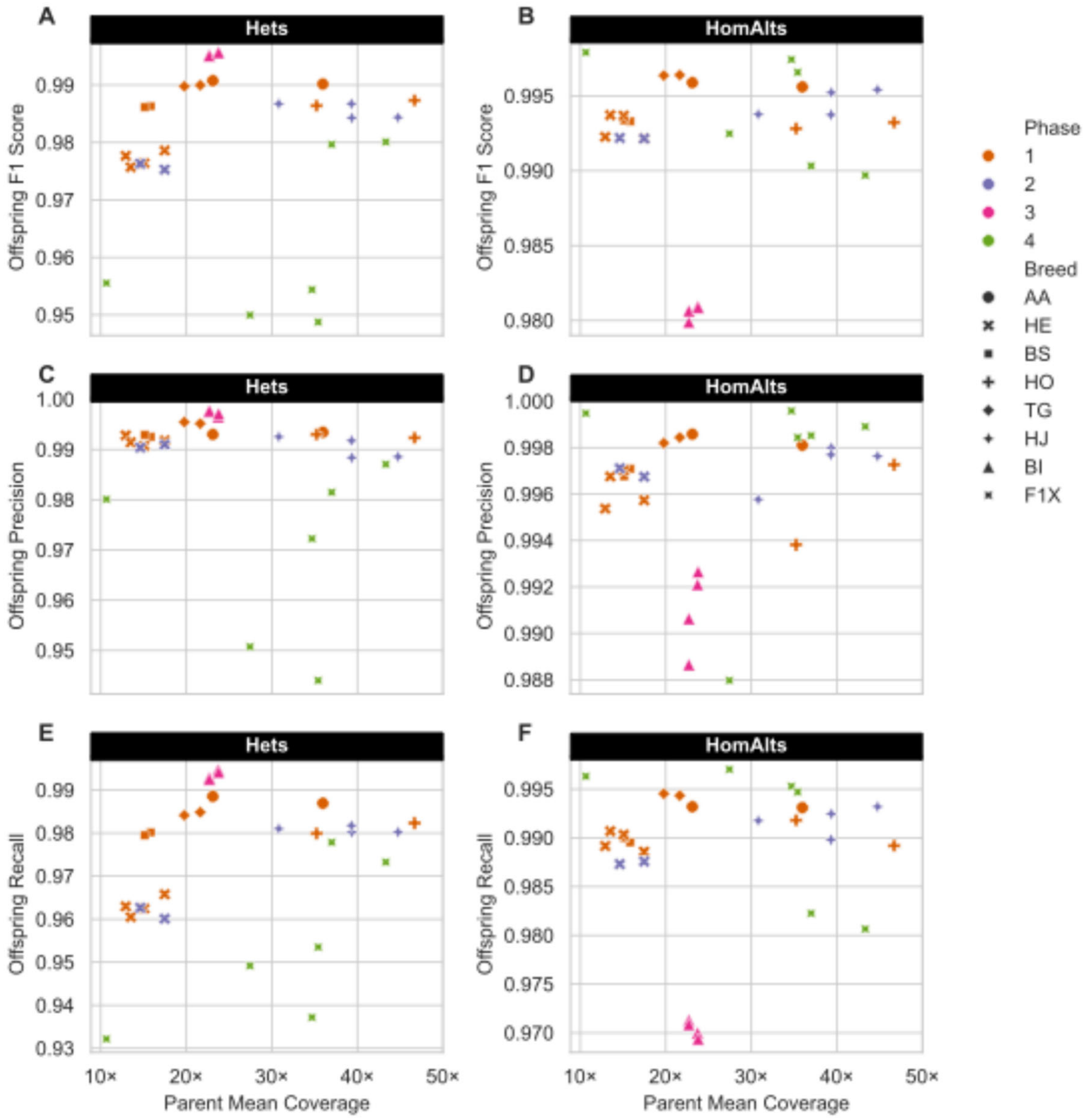

Figure S5. Using bison genomes to reveal DV's knowledge gaps.
The x-axis illustrates the variable sequencing coverage across parent genomes used during training. Note that the y-axes are scaled differently and represent three metrics produced when evaluating training (top: F1 score, middle: precision, bottom: recall), stratified by genotype class (left: Hets, right: HomAlts). Each point represents a new, bovine-trained DV-AF checkpoint created with TrioTrain. Point color designates checkpoints produced over four training phases with marker shapes based on the parent's breed or outgroup. By providing the UMAGv1 population allele frequencies during training (channel #8), we aimed to minimize the consequences of variant calling in samples with low coverage. **F.** Iterations with bison parents (~25× coverage) cluster with low HomAlt recall. Relative to previous iterations, these parents have substantially more HomAlts (~9× more; **Table S6**).

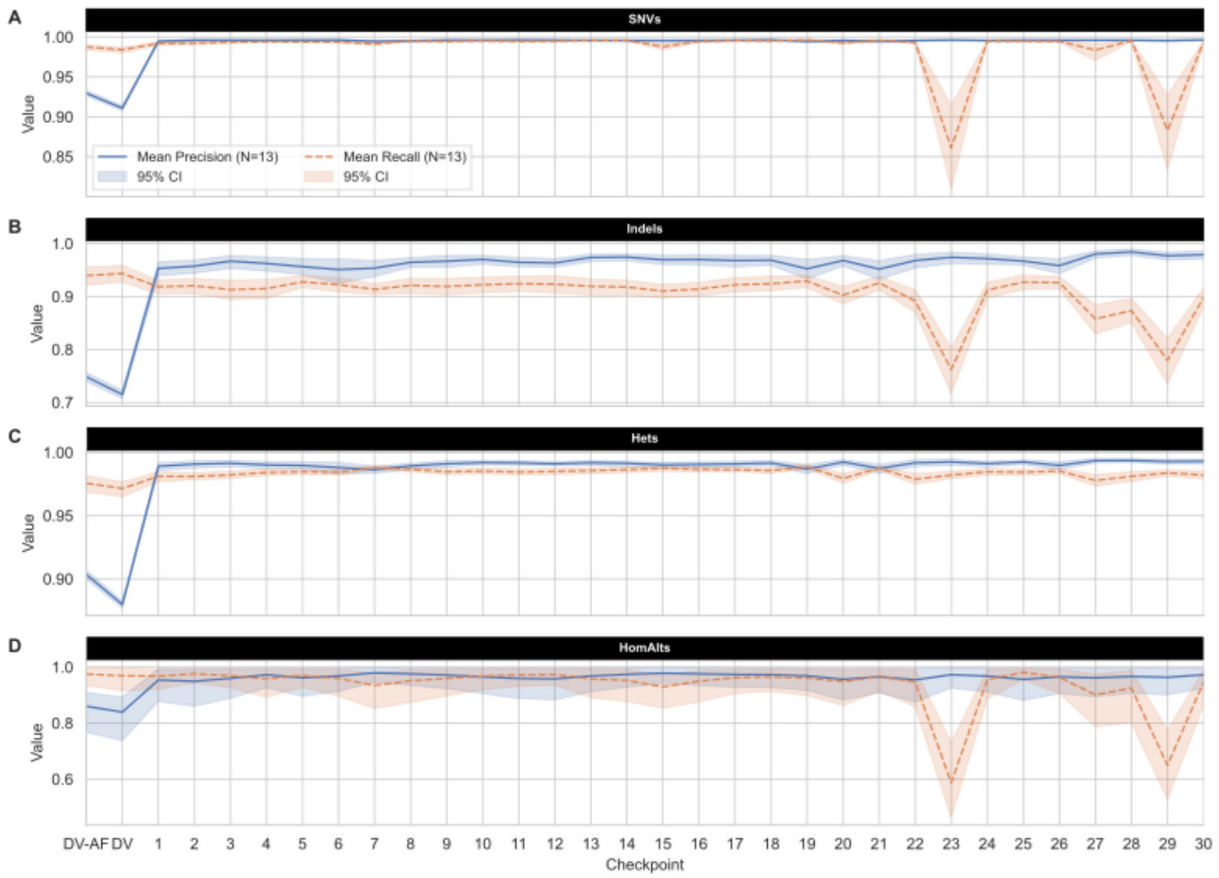

Figure S6. Confirming TrioTrain checkpoints will generalize with cattle genomes. Each panel represents distinct prediction classes made by DeepVariant. Each line represents the mean observed using typical cattle genomes (N=13; **Table S5, top**) after variant calling with each of the 30 successively created DV-AF checkpoints. Precision (solid) and recall (dashed) values are calculated by comparing the variant calls produced with each checkpoint against the UMAG v1 truth labels withheld from training. The shaded area around the lines estimates uncertainty using the 95% confidence interval. Optimal checkpoints created with TrioTrain are labeled numerically in chronological order from left to right on the x-axis. We include two alternative human-trained models, as TrioTrain uses weights from DV to warm-start training with cattle. The x-axis scale differs across panels, where one indicates perfect prediction. In these samples, the human-trained versions (DV, DF-AF) have lower precision relative to the 30 bovine-trained checkpoints. We observe a sudden decline in recall during the first iterations with a real (23) and synthetic (29) hybrid-cross offspring; however, a second iteration with the remaining parent allows the model to recover, indicating the benefits of our trio-based training approach. Although the mean appears stable over the multi-trio approach, we find a statistically significant difference for all metrics, even when excluding the visible outlier checkpoints (DV, DV-AF, 23, 29) and reject the null hypothesis that metric values remain consistent among samples (maximum p-value = 1.319625e-29, Friedman test, where J=13 and K=27, calculated with scipy stats v1.14.1) (Rainio et al. 2024).

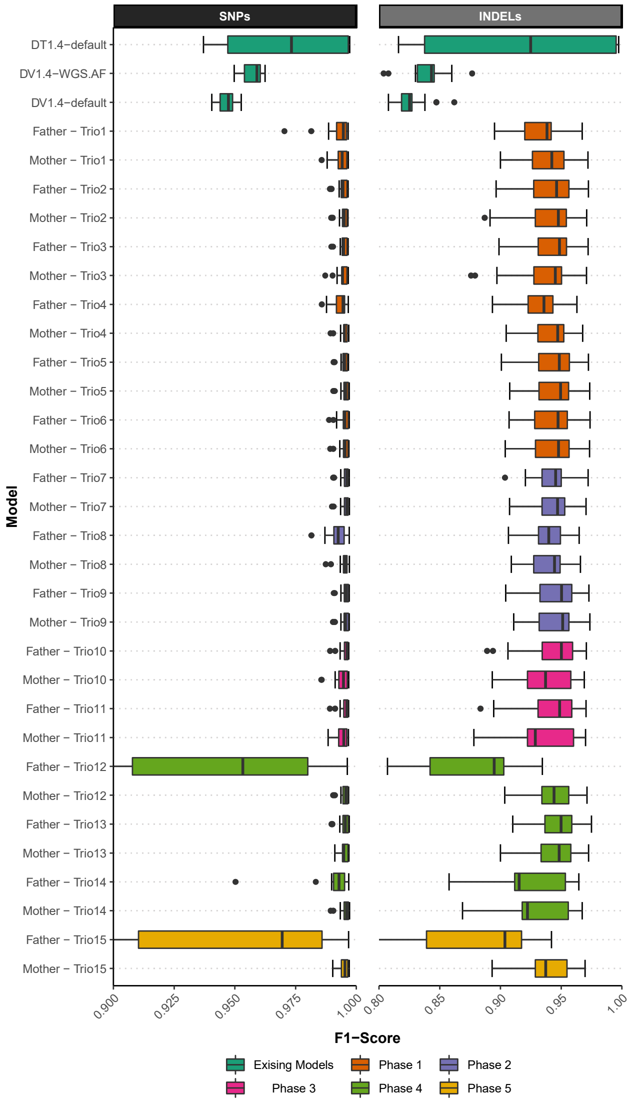

Figure S7. Generalization of the successively built TrioTrain checkpoints with independent bovine genomes trios.

Each box-and-whisker bar represents the distribution of F1 scores observed with a variable number of bovine samples previously withheld by TrioTrain. The x-axis scale begins at 0.9 and 0.8 for SNPs and indels, respectively, where an F1 score of 1 would indicate perfect prediction. Here, we compare model testing metrics with bovine genomes previously unseen during training. The samples vary depending on the model used for variant calling. For the joint-caller, DeepTrio, samples include the real and synthetic hybrid offspring (N=6). With the single-sample callers (DV, DV-AF), samples include all the bovine testing genomes (N=19). With TrioTrain checkpoints, the sample size varies based on the training phase. Through the first three phases, samples include all the bovine testing genomes (N=19). However, training begins with the F1 hybrid trios during phase 4 (Father-Trio12 = C23). Therefore, these samples simultaneously dropped from the test set. As the number of iterations increases, the sample size decreases by one for each pair of models representing a complete bovine trio (N=18;17;16;15). Two checkpoints (Father-Trio 12 = C23, Father-Trio15 = C29) use labeled examples from the father (Angus) but were evaluated using the real and synthetic hybrid offspring, respectively. These unique cases extend a whisker out of view (SNP minimum F1 scores = 0.826 and 0.863, respectively). The sizeable change in performance occurs as DeepVariant adjusts to substantial differences between training and evaluation data (**Figure S3**) or the new error profile with a synthetic sample. Training was halted after one round with synthetic offspring because of the decrease in generalization compared to the previous phase (mean F1 score: Phase 4 = 0.97814, Phase 5 =0.96241).

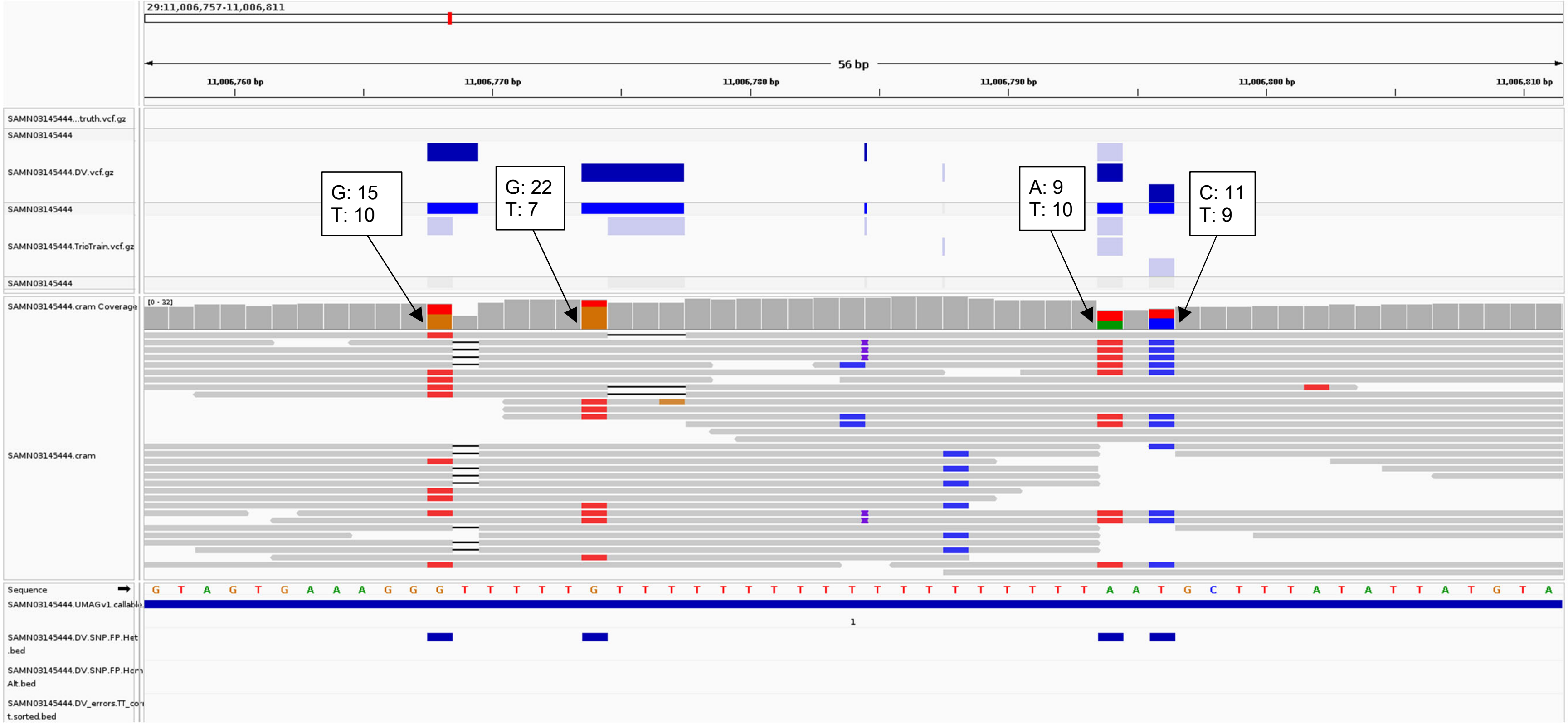

Figure S8. Visualizing heterozygous SNVs identified by the human-trained DeepVariant within a homopolymer region in a cattle genome.

	29:11006768	29:11006774	29:11006794	29:11006796
UMAGv1 Truth	./.	./.	./.	./.
DV	0/1	0/1	0/1	0/1
28	./.	./.	./.	./.

The above visualization depicts reads (Illumina WGS) from a bovine genome (Dominette: SAMN03145444), which is the same individual as the current cattle reference genome, ARS-UCD1.2 (Rosen et al., 2020). Allele counts are provided in the text boxes for each variant. These four loci are within a known homopolymer (25bp Simple Repeat in UCSC Genome Browser, Apr. 2018). The human-trained DeepVariant (DV) correctly identified these as four heterozygous SNVs. However, since all four were missing from the GATK-based UMAGv1 truth VCF, these variants remain uncalled with the TrioTrain checkpoint (28). These errors are expected since the bovine truth labels were based on a single sequencing platform that can systematically introduce base-pair extension errors in homopolymers. Additional information from multiple sequencing platforms would likely improve truth quality in repetitive regions, such as the ongoing efforts to produce diploid, telomere-to-telomere (T2T) high-quality bovine assemblies.

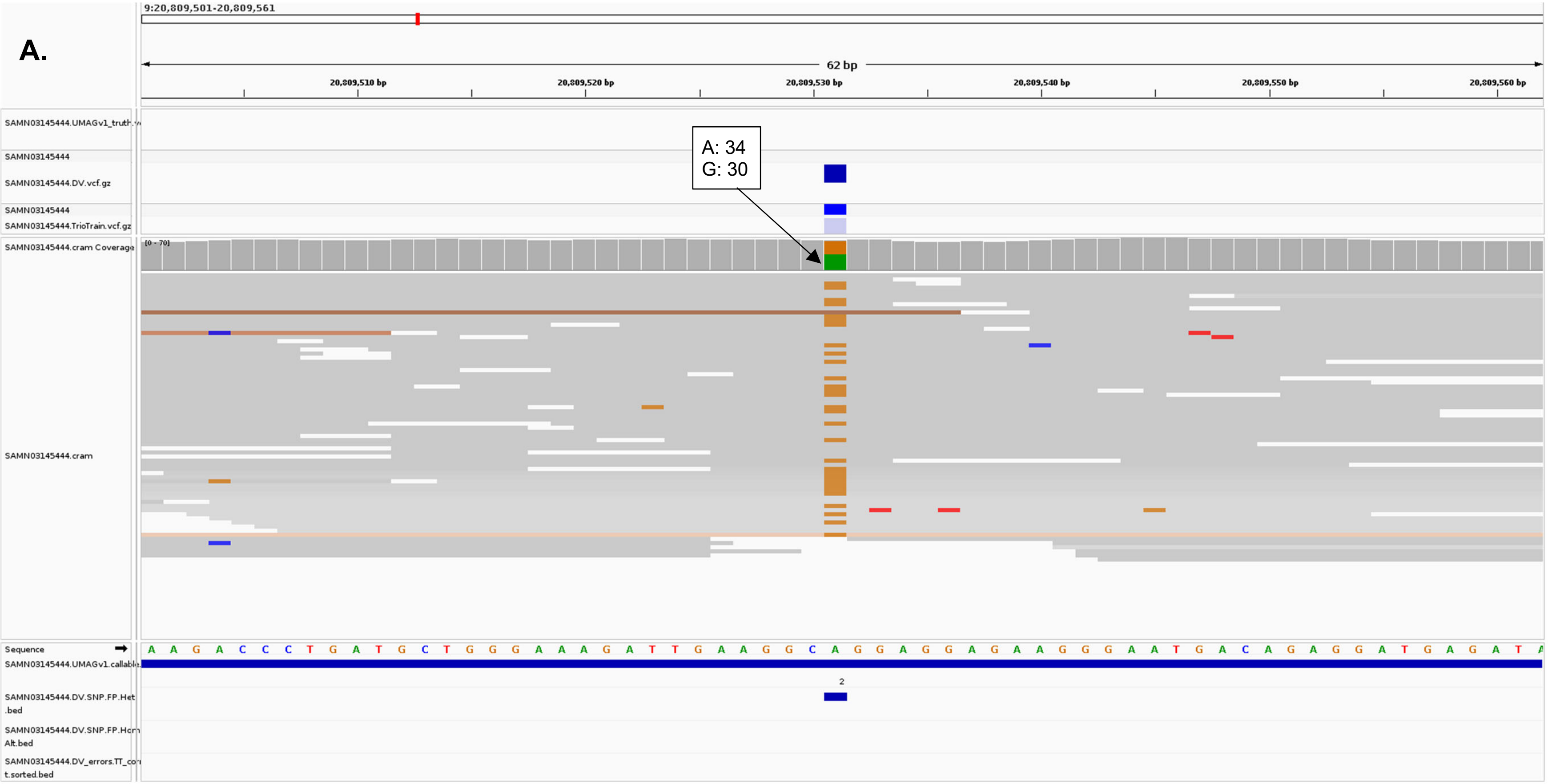
A.
9:20,809,501-20,809,561
62 bp
20,809,510 bp
20,809,520 bp
20,809,530 bp
20,809,540 bp
20,809,550 bp
20,809,560 bp
SAMN03145444
SAMN03145444.DV.vcf.gz
SAMN03145444
SAMN03145444.TrioTrain.vcf.gz
SAMN03145444.cram Coverage
[0 - 70]
A: 34
G: 30
SAMN03145444.cram
Sequence
SAMN03145444.DV.SNP.FP.Het
.bed
2

B.

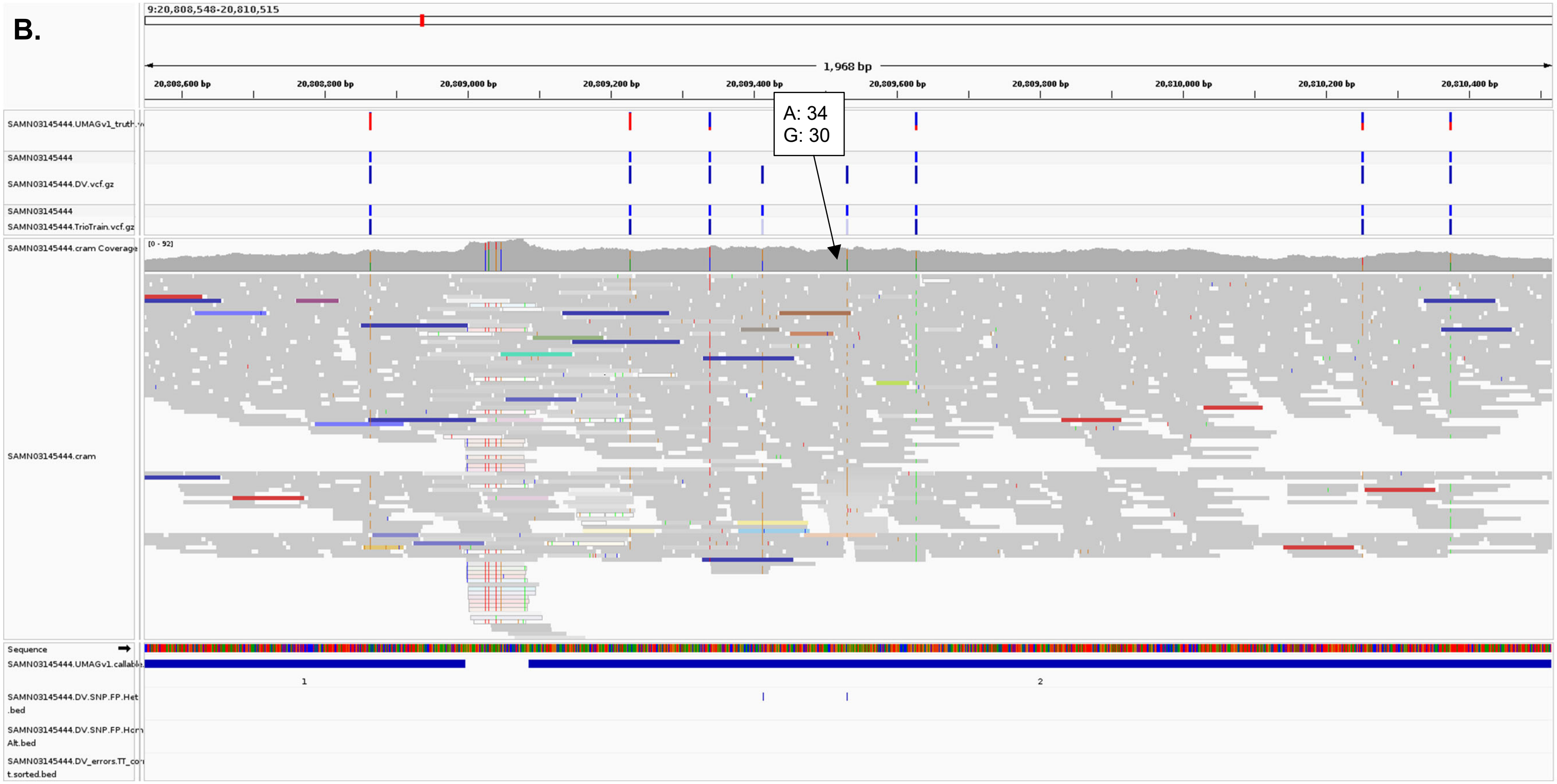

Figure S9. Visualizing a heterozygous SNV identified by the human-trained DeepVariant within a known LINE in a cattle genome.

	9:20809531
UMAGv1 Truth	./.
DV	0/1
28	./.

The above visualizations (**A**: zoomed in on previous page, **B**: a broader view) depict reads (Illumina WGS) from a bovine genome (Dominette: SAMN03145444), which is the same individual as the current cattle reference genome, ARS-UCD1.2 (Rosen et al., 2020). Allele counts for one variant are provided in the text boxes. This locus is within a known LINE (695bp 'RTE-BovB' in UCSC Genome Browser, Apr. 2018). The human-trained DeepVariant (DV) correctly identified this as a heterozygous SNV. However, since the correct genotype was missing from the GATK-based UMAGv1 truth VCF, this variant remains uncalled with the TrioTrain checkpoint (28), as expected. The higher depth of coverage relative to mean coverage (+18.5×) and the context surrounding the variant (LINE) indicate the limitations of short-read alignment within this repetitive region.

A.

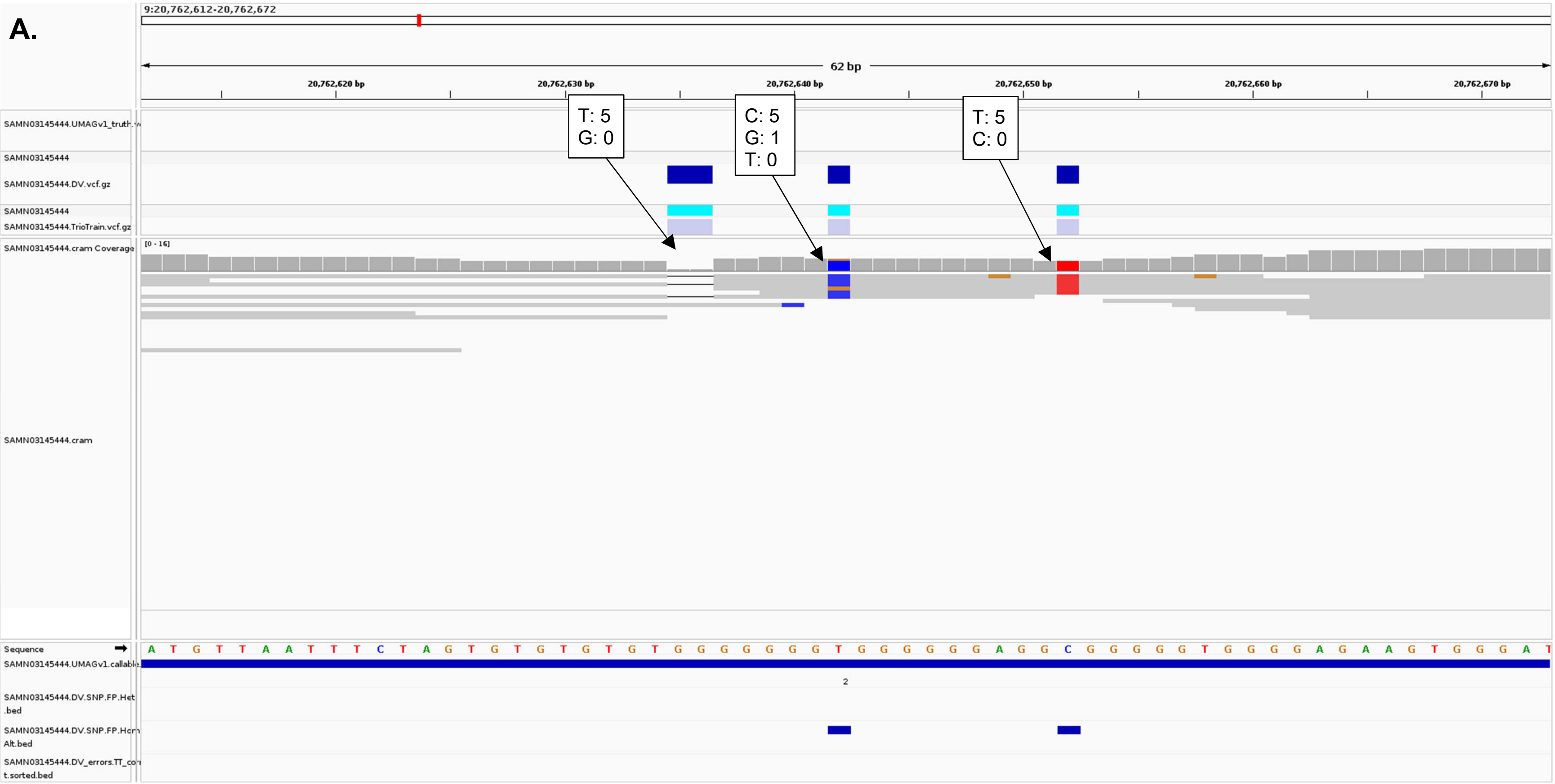
9:20,762,612-20,762,672
62 bp
20,762,620 bp
20,762,630 bp
20,762,640 bp
20,762,650 bp
20,762,660 bp
20,762,670 bp
T: 5
G: 0
C: 5
G: 1
T: 0
T: 5
C: 0
SAMN03145444.DV.vcf.gz
SAMN03145444.TrioTrain.vcf.gz
SAMN03145444.cram Coverage
[0 - 16]
SAMN03145444.cram
Sequence
SAMN03145444.DV.SNP.FP.Het .bed
SAMN03145444.DV.SNP.FP.Hom Alt.bed

B.

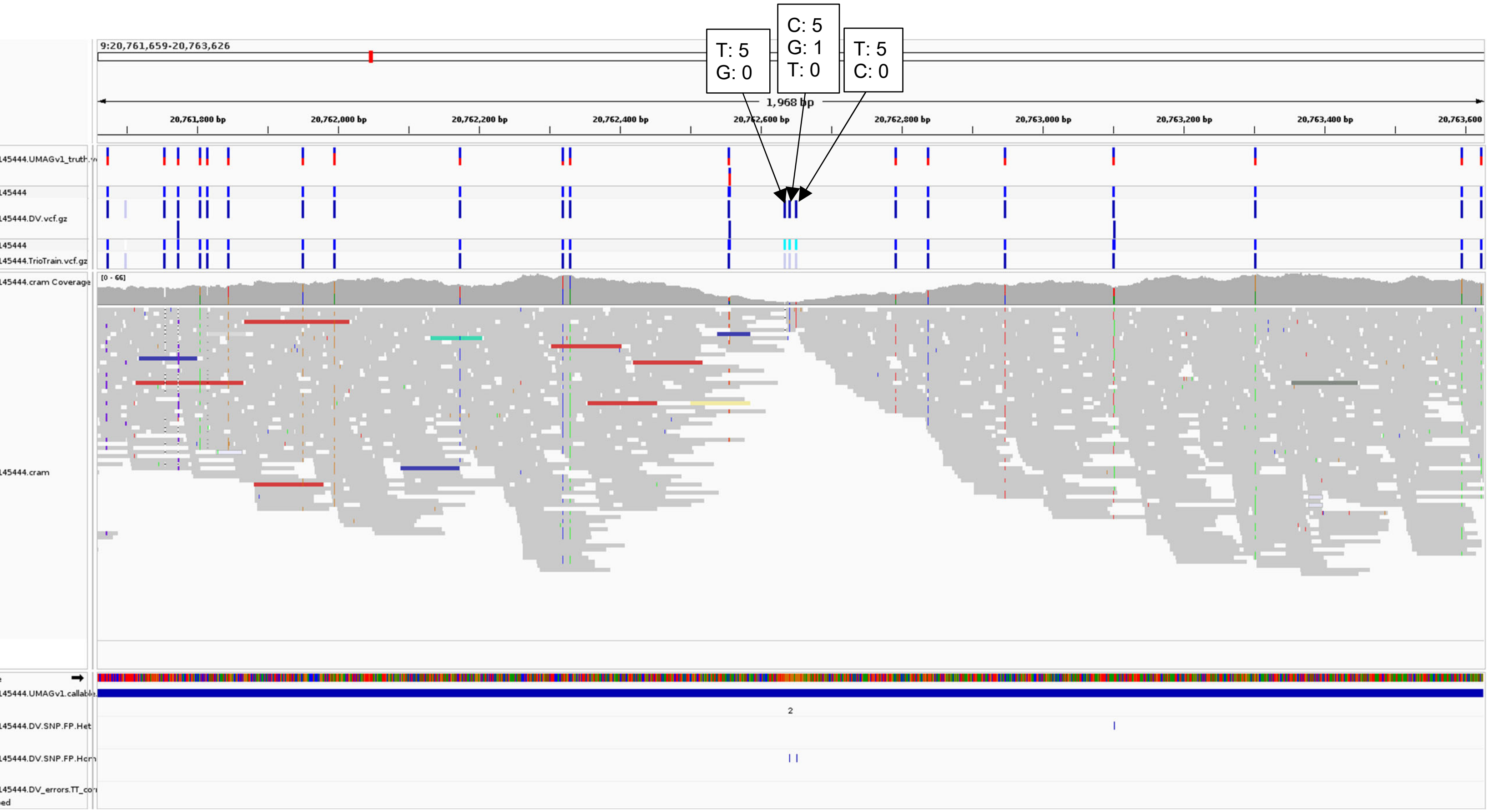

Figure S10. Visualizing an ambiguous homozygous SNV identified the human-trained DeepVariant in a cattle genome.

	9:20762634-20762636	9:20762642	9:20762652
UMAGv1 Truth	./.	./.	./.
DV	1/1	1/1	1/1
28	./.	./.	./.

The above visualizations (**A**: zoomed in on previous page, **B**: a broader view) depict reads (Illumina WGS) from a bovine genome (Dominette: SAMN03145444), which is the same individual as the current cattle reference genome, ARS-UCD1.2 (Rosen et al., 2020). Allele counts are provided in the text boxes for each variant. These three loci are within a known LTR (894bp 'ERVL-E-int' in UCSC Genome Browser, Apr. 2018). The human-trained DeepVariant (DV) identified these as three homozygous alternate SNVs. However, since all four were missing from the GATK-based UMAGv1 truth VCF, these variants remain uncalled with the TrioTrain checkpoint (28). The lower depth of coverage relative to mean coverage (~39.5×) and the context surrounding the variant (retrotransposon) demonstrate the limitations of defining truth from short-read alignment for this transposable element. Given that the reads and reference represent the same individual and all surrounding variation is heterozygous, these variants could plausibly be heterozygous and thus potentially genuine FPs introduced by the human-trained DV. Given that the default version of DeepVariant is known to make mistakes in humans with similar depth of coverage (<10×), incorporating the allele frequency channel when re-training (28) helps to correct allele sampling bias caused by low coverage.

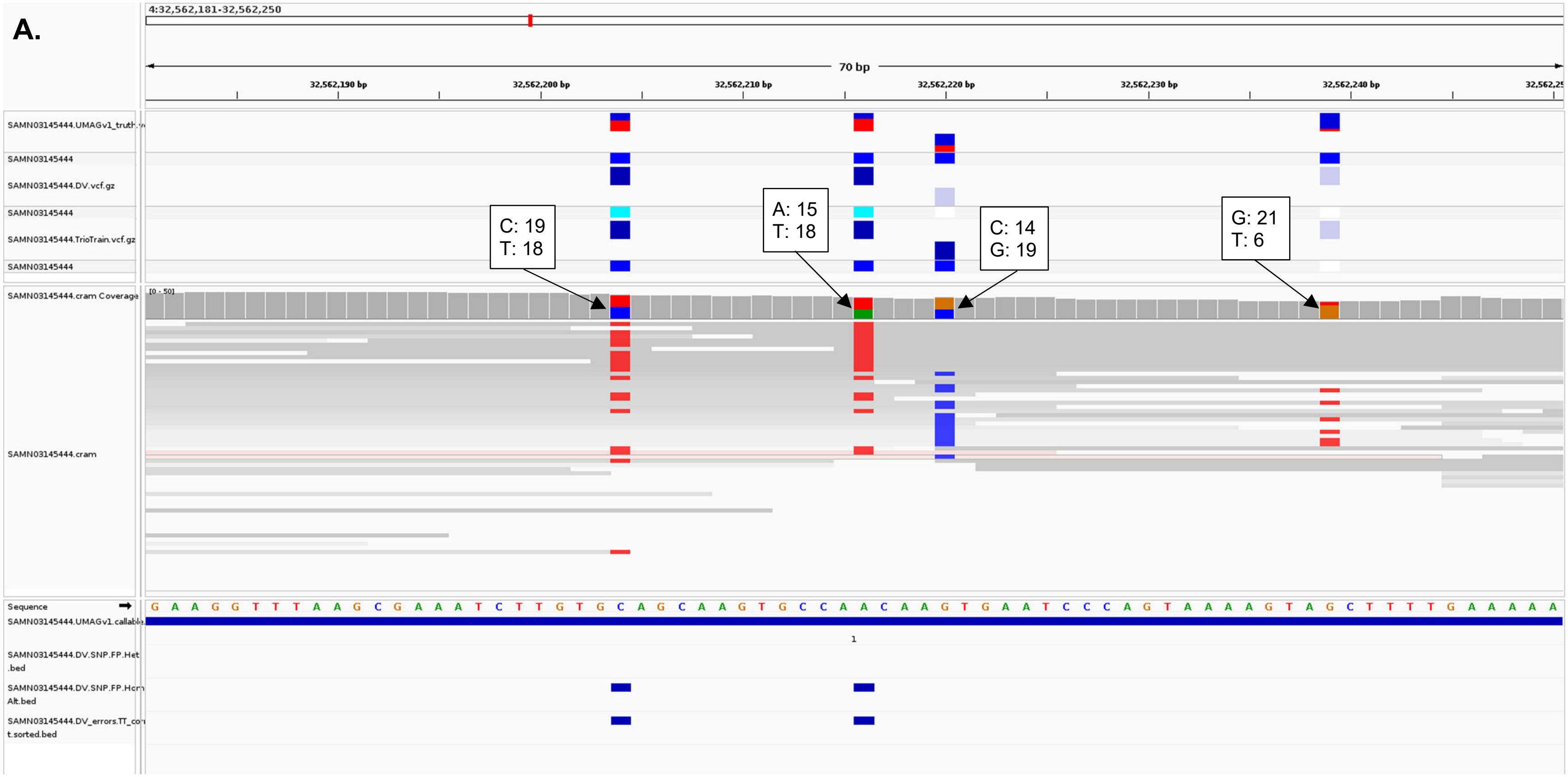

A.
4:32,562,181-32,562,250
70 bp
32,562,190 bp
32,562,200 bp
32,562,210 bp
32,562,220 bp
32,562,230 bp
32,562,240 bp
SAMN03145444
SAMN03145444.DV.vcf.gz
SAMN03145444.TrioTrain.vcf.gz
SAMN03145444.cram
Sequence
C: 19
T: 18
A: 15
T: 18
C: 14
G: 19
G: 21
T: 6

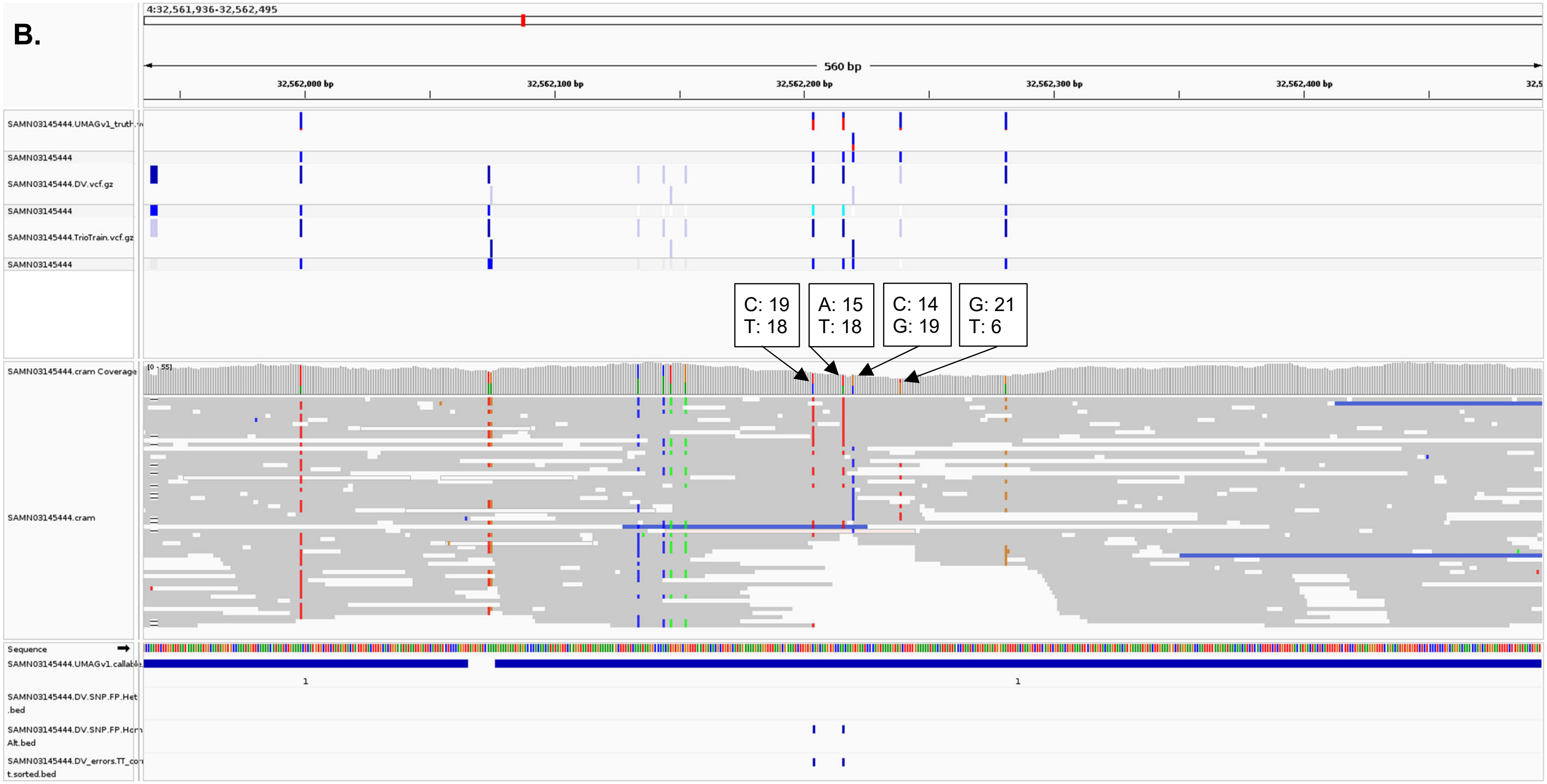

Figure S11. Visualizing genuine errors from the human-trained DeepVariant corrected after extending with TrioTrain.

	4:32562204	4:32562216	4:32562220	4:32562239
UMAGv1 Truth	0/1	0/1	0/1	0/1
DV	1/1	1/1	./.	./.
28	0/1	0/1	0/1	./.

The above visualizations (**A**: zoomed in on previous page, **B**: a broader view) depict reads (Illumina WGS) from a bovine genome (Dominette: SAMN03145444), which is the same individual as the current cattle reference genome, ARS-UCD1.2 (Rosen et al., 2020). Allele counts are provided in the text boxes for each variant. The human-trained DeepVariant (DV) incorrectly identified these four loci, with two homozygous alternate SNVs, and ignored the others. However, since all four were included in the GATK-based UMAGv1 truth VCF, three variants were correctly called with the TrioTrain checkpoint (28). Given a similar depth of coverage relative to mean coverage (-8.5×), the two genuine homozygous FP errors indicate that DeepVariant has incorporated underlying assumptions of heterozygosity based on prior experience with the human genome. We suspect that lower coverage (<10×) of the alternate allele explains why both checkpoints missed the fourth variant entirely.

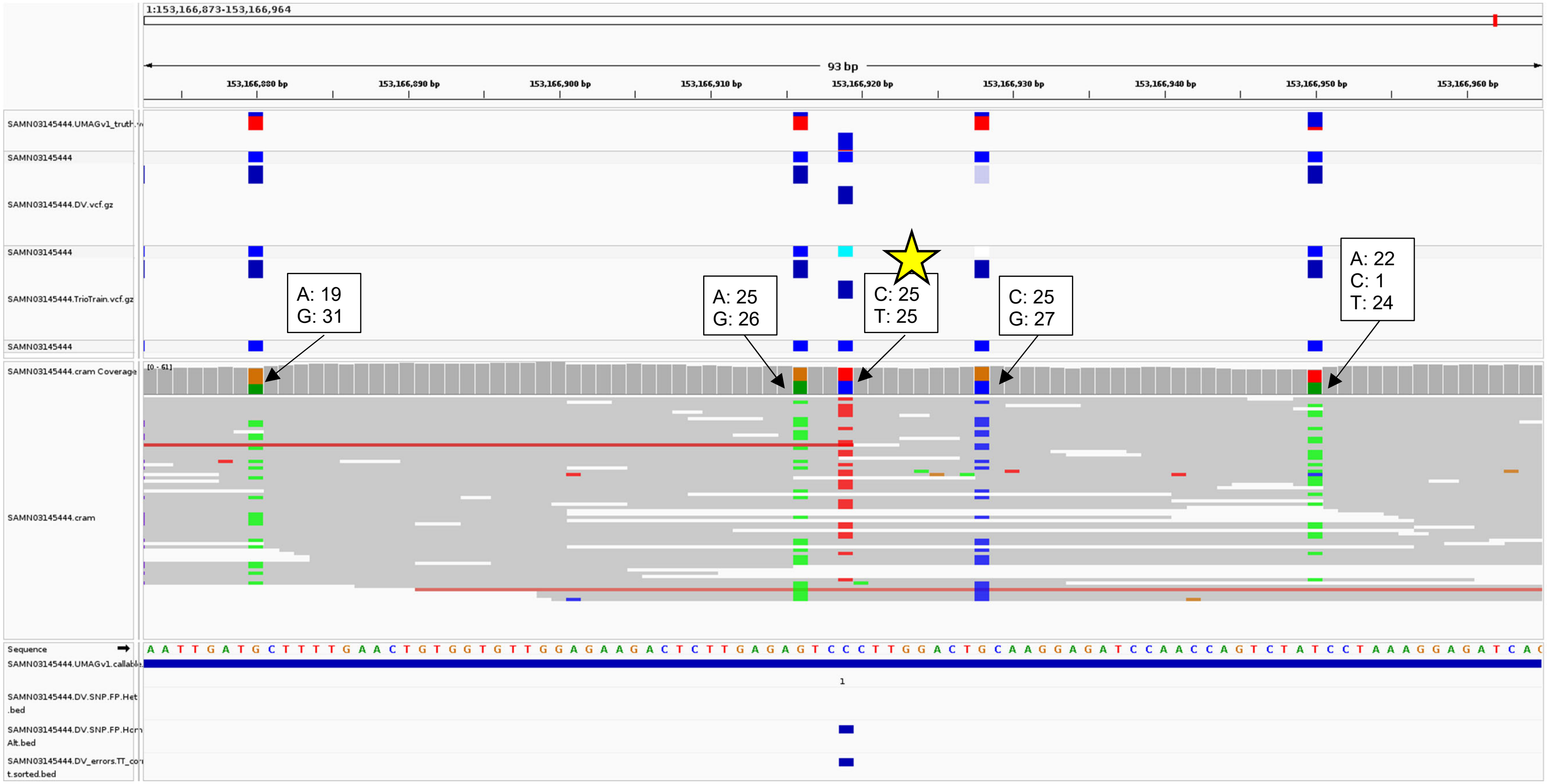

Figure S12. Visualizing genuine errors from the human-trained DeepVariant corrected after extending with TrioTrain.

	1:153166919
UMAGv1 Truth	0/1
DV	1/1
28	0/1

The above visualization depicts reads (Illumina WGS) from a bovine genome (Dominette: SAMN03145444), which is the same individual as the current cattle reference genome, ARS-UCD1.2 (Rosen et al., 2020). Allele counts are provided in the text boxes for each variant, with the error emphasized (yellow star). These loci are within a known LINE (1302bp 'RTE-BovB' in UCSC Genome Browser, Apr. 2018). The human-trained DeepVariant (DV) incorrectly identified one variant as a homozygous alternate SNV. However, since the correct genotype was included in the GATK-based UMAGv1 truth VCF, this variant is correctly called with TrioTrain checkpoint (28). Given a similar depth of coverage relative to mean coverage (+4.5×), this genuine homozygous FP error indicates that DeepVariant has incorporated underlying assumptions of copy number variation based on prior experience with the human genome.

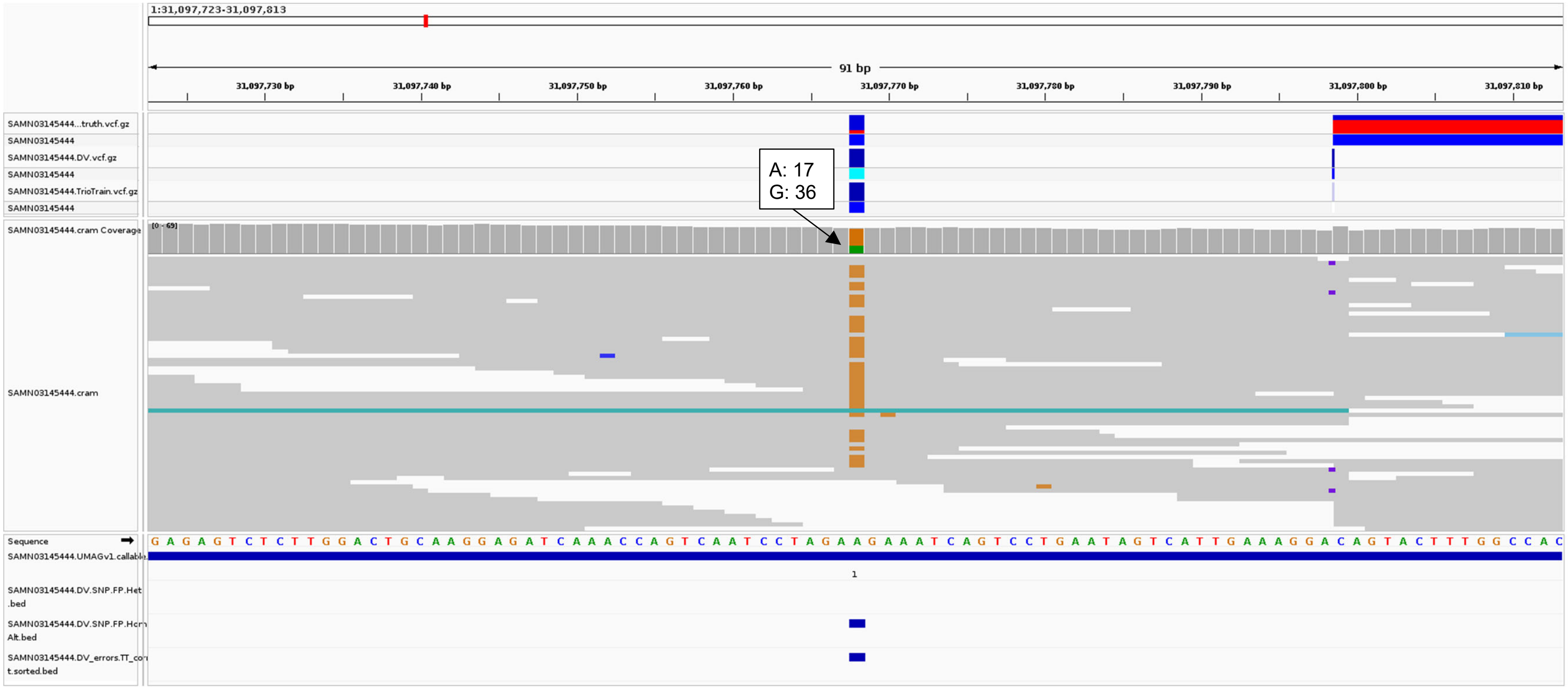

Figure S13. Visualizing genuine errors from the human-trained DeepVariant corrected after extending with TrioTrain.

	1:31097768
UMAGv1 Truth	0/1
DV	1/1
28	0/1

The above visualization depicts reads (Illumina WGS) from a bovine genome (Dominette: SAMN03145444), which is the same individual as the current cattle reference genome, ARS-UCD1.2 (Rosen et al., 2020). Allele counts are provided in the text boxes for the variant. The human-trained DeepVariant incorrectly identifies this variant as a homozygous alternate within a known LINE (302bp 'RTE-BovB' in UCSC Genome Browser, Apr. 2018). However, since the correct genotype was included in the GATK-based UMAGv1 truth VCF, this variant is correctly called with TrioTrain checkpoint (28). Given a similar depth of coverage relative to mean coverage (+7.5×), the genuine homozygous FP error indicates that DeepVariant has incorporated underlying assumptions of copy number variation based on prior experience with the human genome.

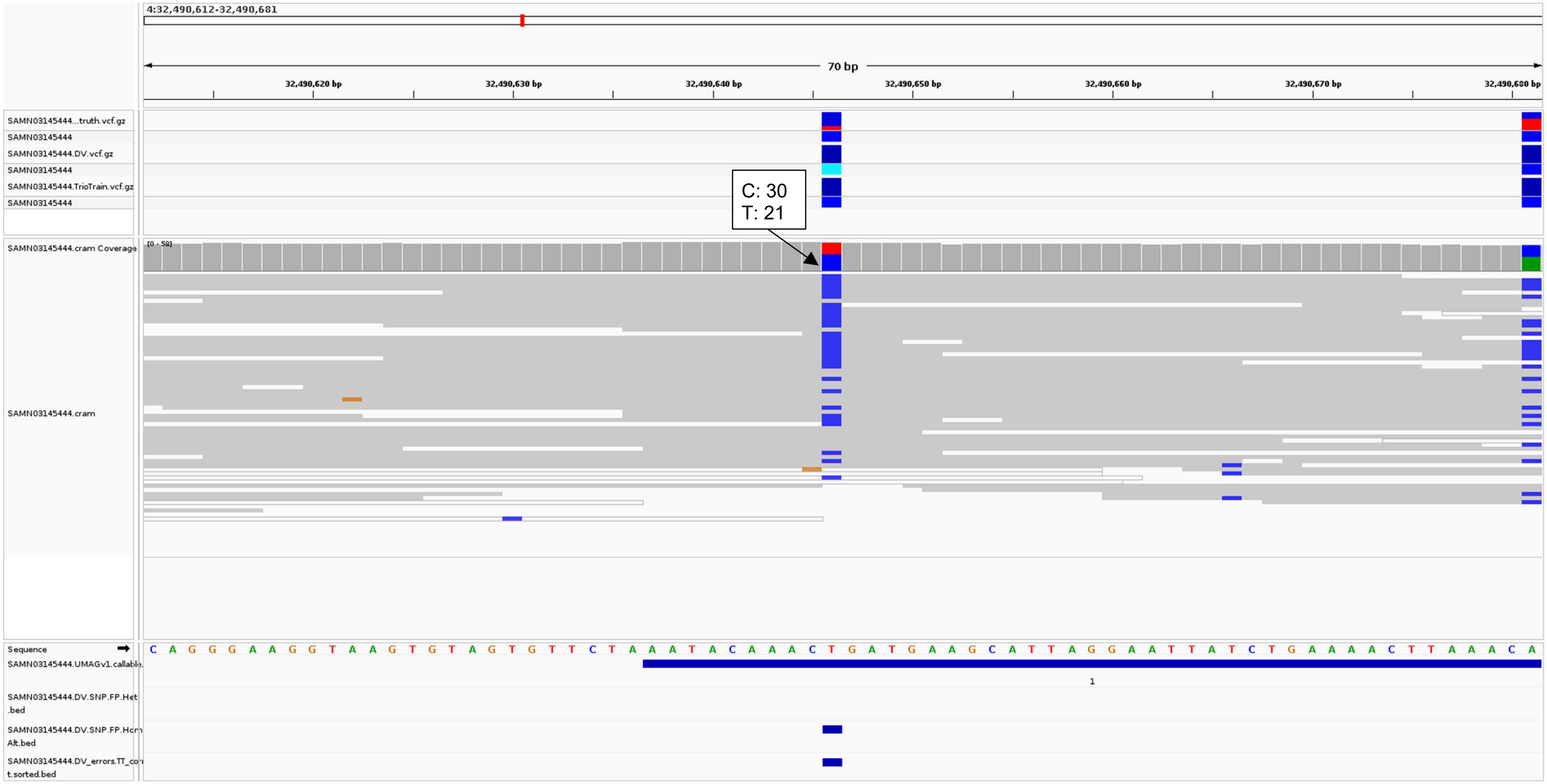

Figure S14. Visualizing genuine errors from the human-trained DeepVariant corrected after extending with TrioTrain.

	4:32490646
UMAGv1 Truth	0/1
DV	1/1
28	0/1

The above visualization depicts reads (Illumina WGS) from a bovine genome (Dominette: SAMN03145444), which is the same individual as the current cattle reference genome, ARS-UCD1.2 (Rosen et al., 2020). Allele counts are provided in the text boxes for the variant. The human-trained DeepVariant incorrectly identifies this variant as a homozygous alternate. However, since the correct genotype was included in the GATK-based UMAGv1 truth VCF, this variant is correctly called with TrioTrain checkpoint (28). Given a similar depth of coverage relative to mean coverage (+5.5×), this genuine homozygous FP error indicates that DeepVariant has incorporated underlying assumptions of heterozygosity based on prior experience with the human genome.

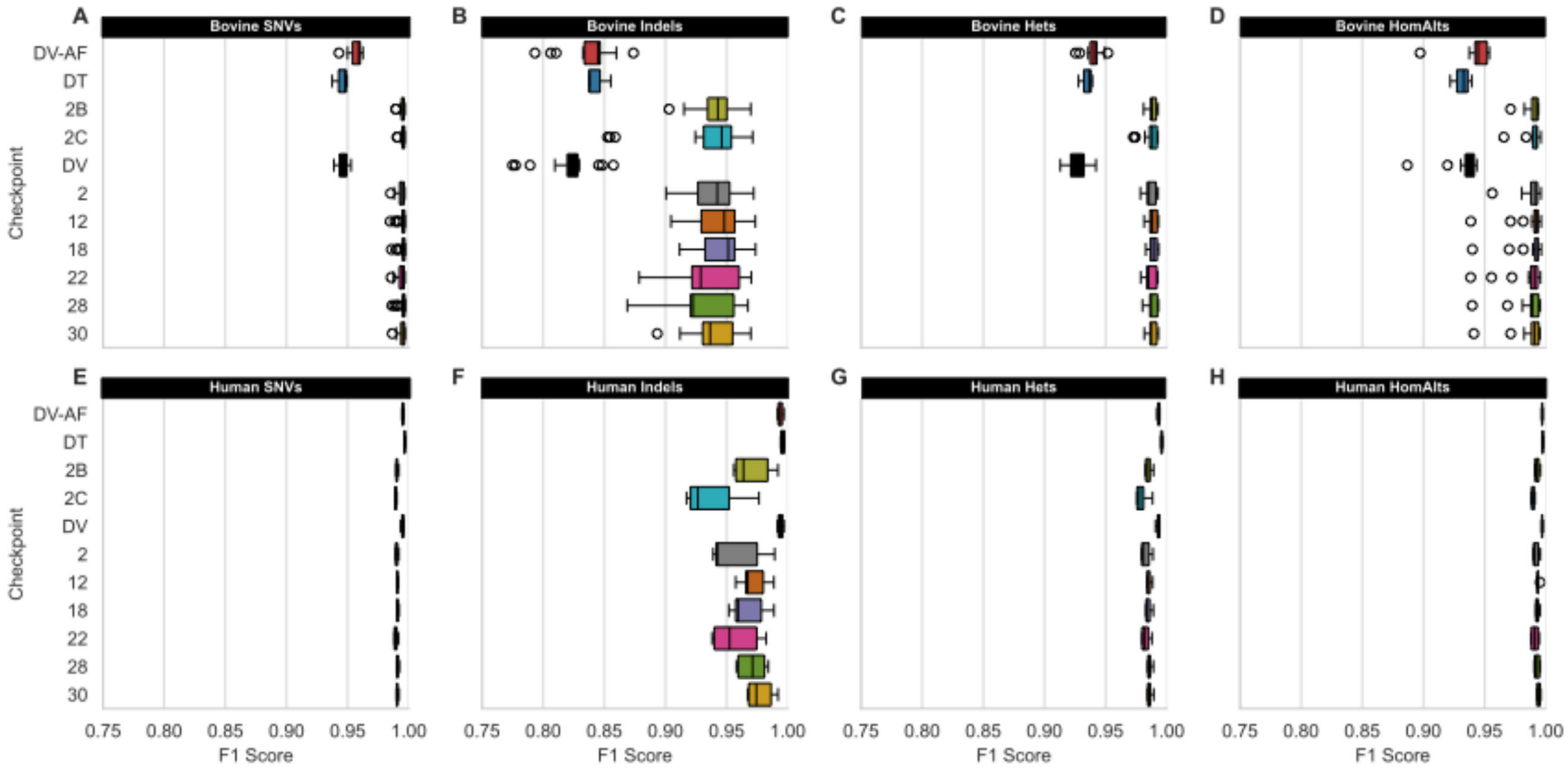

Figure S15. Comparing checkpoint generalization across species.
Three human-trained versions of DeepVariant (DV, DV-AF, DT) were evaluated against eight bovine-trained DV-AF checkpoints created with TrioTrain. Each box-and-whisker bar represents the distribution of F1 scores observed with a consistent set of either bovine genomes (top; N=19, except for DT where N=3 F1 cross offspring) or the human GIAB trios (bottom; N=6). Metrics are stratified by variant type (SNV, indel) and genotype class (Hets, HomAlts). The checkpoints created with TrioTrain underperform, specifically with indels relative to other classifications.
The human-trained versions of DeepVariant underperform in cattle relative to any bovine-trained checkpoints. In comparison, performance remains relatively similar when applying bovine-trained checkpoints to humans, except for indels.

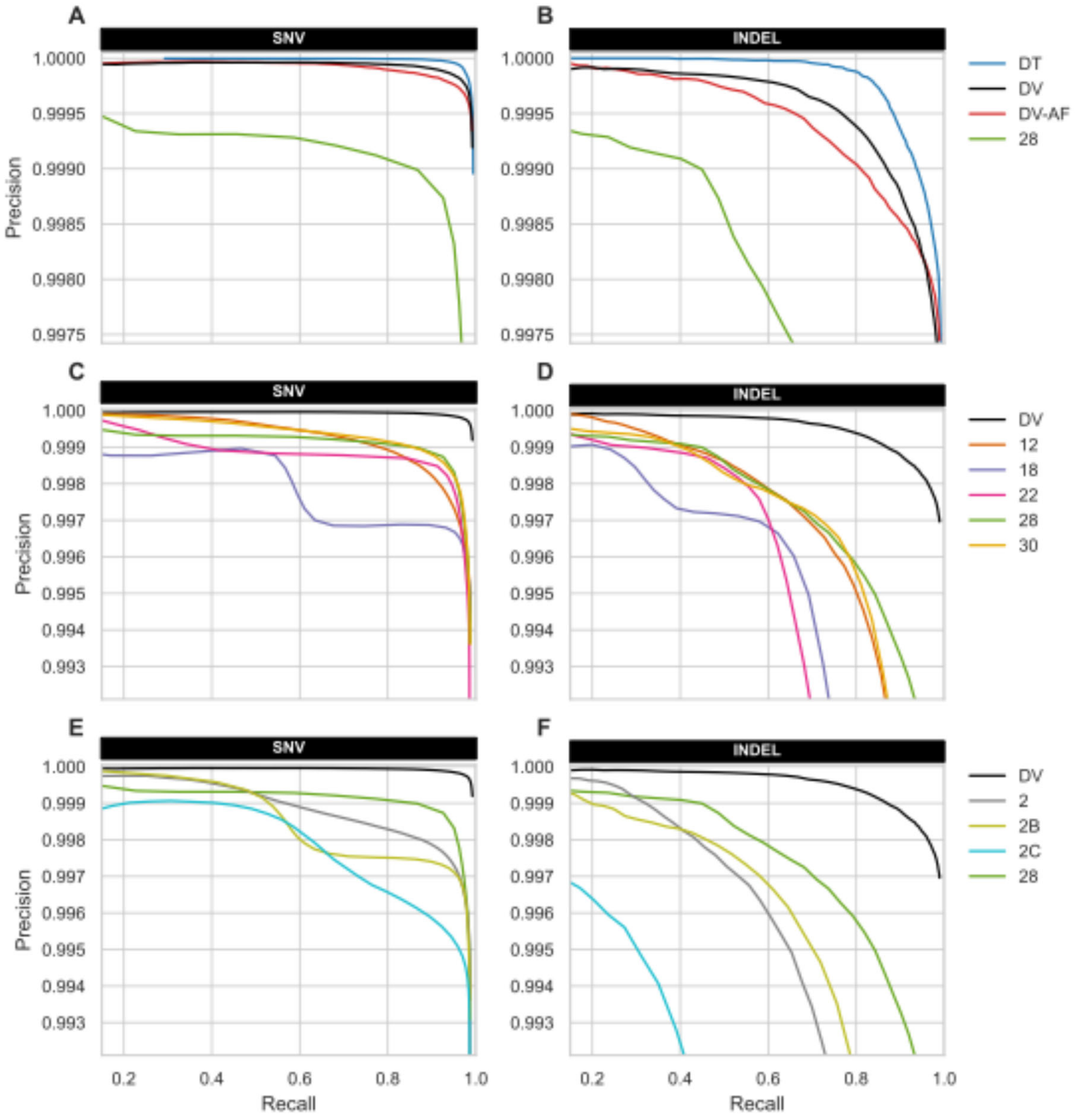

Figure S16. Comparing precision-recall curves in 10 different checkpoints.
We use a single human genome (HG002) to quantify the impact of a single-trio approach relative to the multi-trio approach. Each panel compares precision and recall curves across multiple checkpoints, further stratified by variant type (right: SNV, left: indels). The top two panels compare bovine-trained checkpoint 28 against all existing human-trained versions of DeepVariant (DV, DV-AF, DT). Although the curve for the TrioTrain checkpoint is lower than existing versions, we emphasize that the magnitude of the difference is minor (SNV: <0.001). The middle panels contrast the default version (DV) against the multi-trio checkpoints created over multiple TrioTrain phases (phase 1: 12, phase 2: 18, phase 3: 22, phase 4: 28, phase 5: 30). Out of the five new checkpoints, checkpoints 28 and 30 provide similar performance in HG002, which is expected based on Figure 3. The final two panels contrast the default version (DV) against three single-trio checkpoints (2, 2B, 2C). There is a slight difference in the curves, with the multi-trio checkpoint performing better than all three single-trio checkpoints.

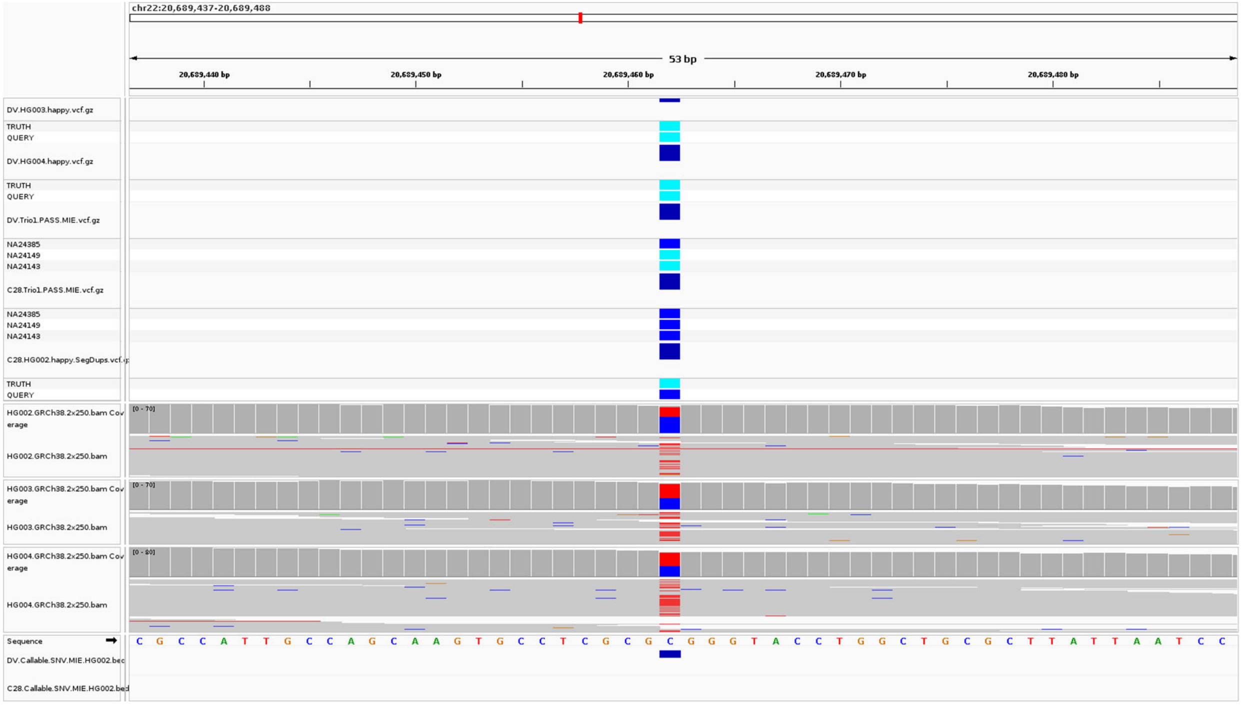

Figure S17. Discordant SNV found in a GIAB trio at Chr22:20689462

	HG002	HG003	HG004	Summary
GIAB Truth	1/1	1/1	1/1	
DV	0/1	1/1	1/1	Discordant due to FN in HG002
28	0/1	0/1	0/1	Concordant, but FN in all three

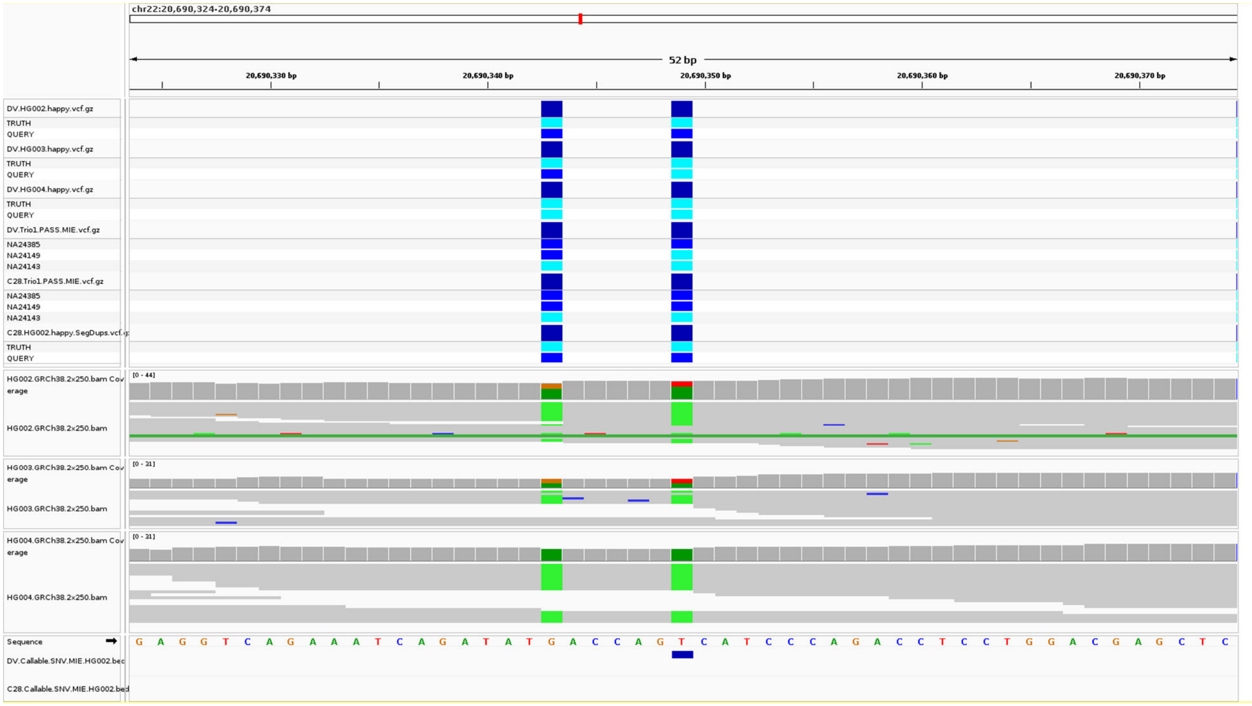

Figure S18. Discordant SNV found in a GIAB trio at Chr22:20690349

	HG002	HG003	HG004	Summary
GIAB Truth	1/1	1/1	1/1	
DV	0/1	1/1	1/1	Discordant due to FN in HG002
28	0/1	0/1	1/1	Concordant, but FN in HG002 and HG003

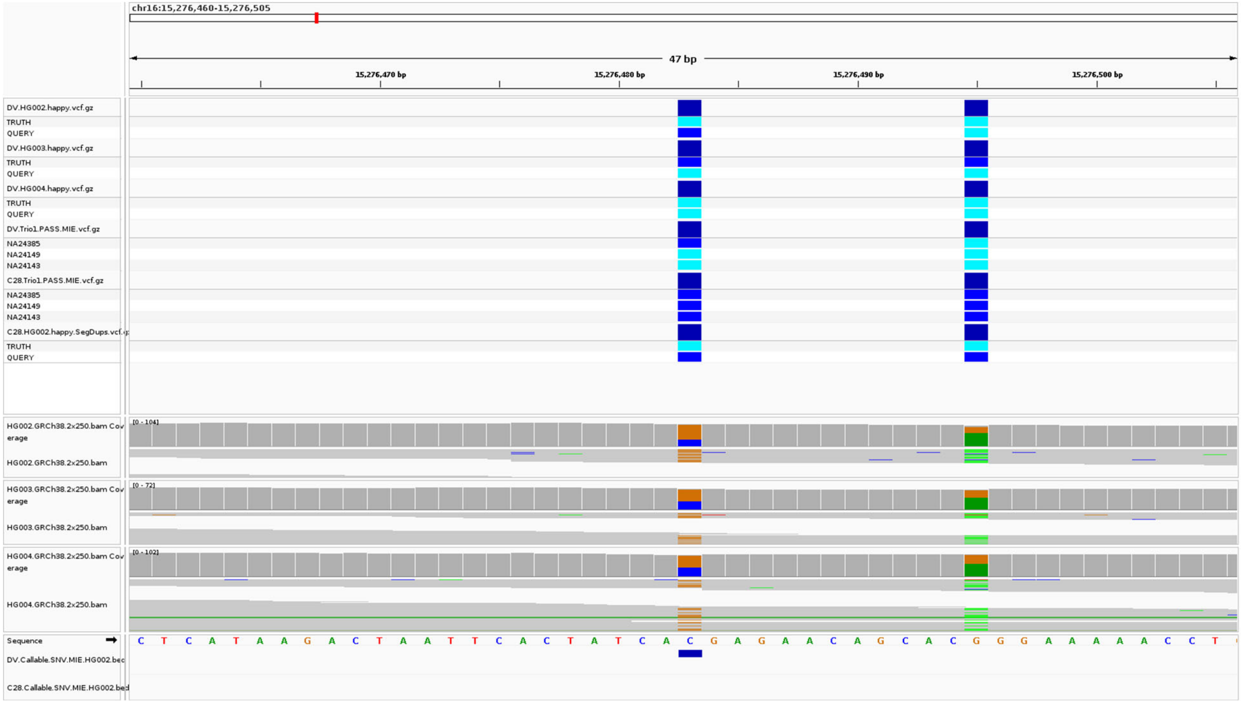

Figure S19. Discordant SNV found in a GIAB trio at Chr16:15276483

	HG002	HG003	HG004	Summary
GIAB Truth	1/1	0/1	1/1	
DV	0/1	1/1	1/1	Discordant due to FP in HG003 and FN in HG002
28	0/1	0/1	0/1	Concordant, but FN in HG002 and HG004

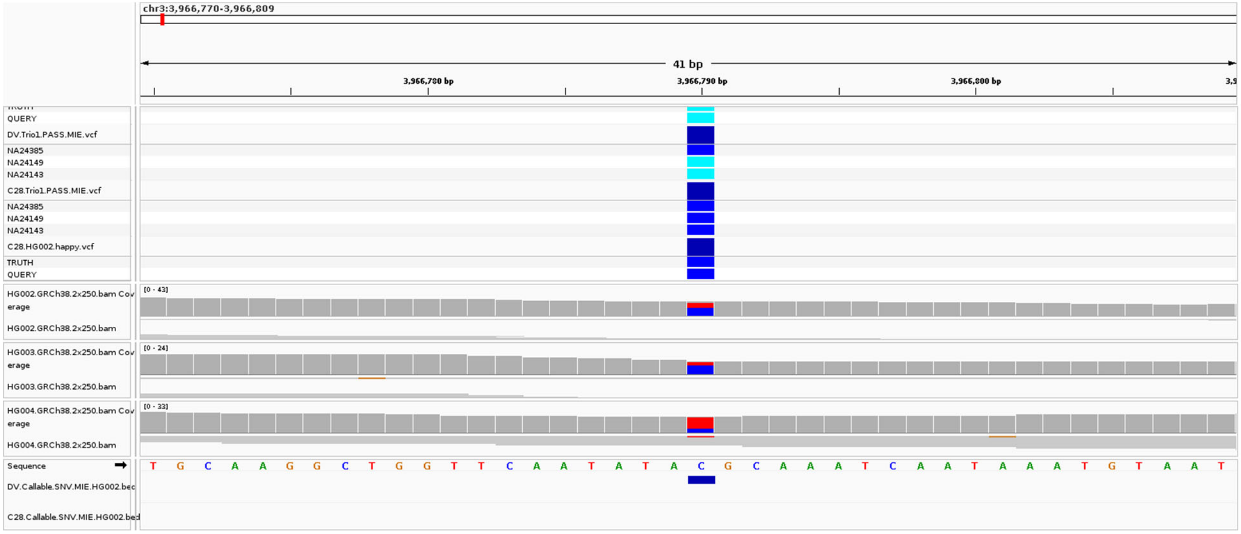

Figure S20. Discordant SNV found in a GIAB trio at Chr3:3966790

	HG002	HG003	HG004	Summary
GIAB Truth	0/1	0/1	1/1	
DV	0/1	1/1	1/1	Discordant due to FP in HG003
28	0/1	0/1	0/1	Concordant, but FN in HG004

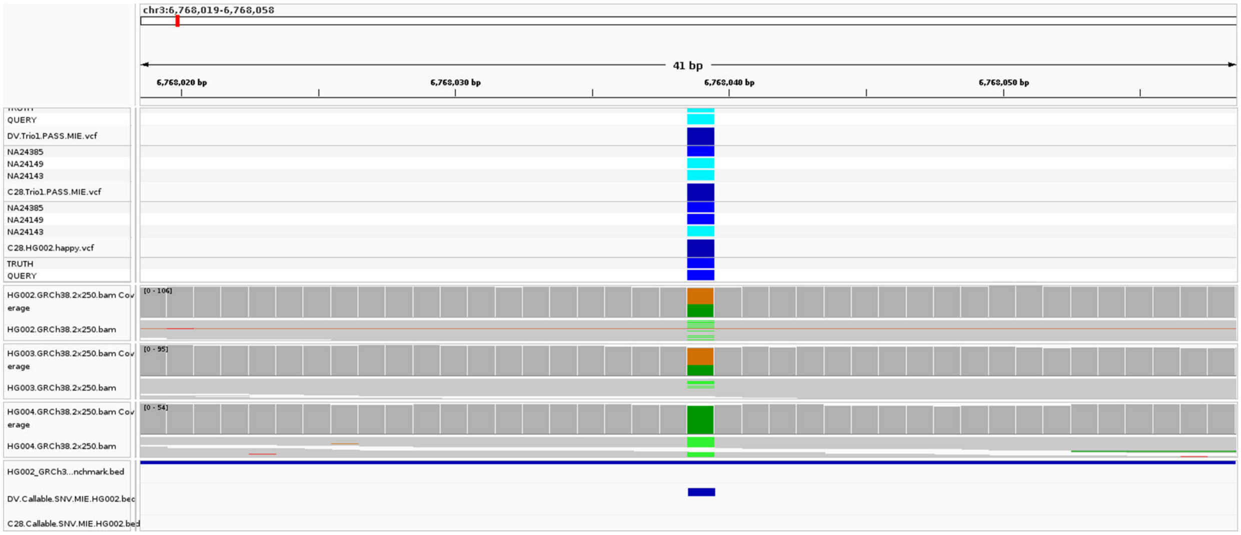

Figure S21. Discordant SNV found in a GIAB trio at Chr3:6768039

	HG002	HG003	HG004	Summary
GIAB Truth	0/1	0/1	1/1	
DV	0/1	1/1	1/1	Discordant due to FP in HG003
28	0/1	0/1	1/1	Correct

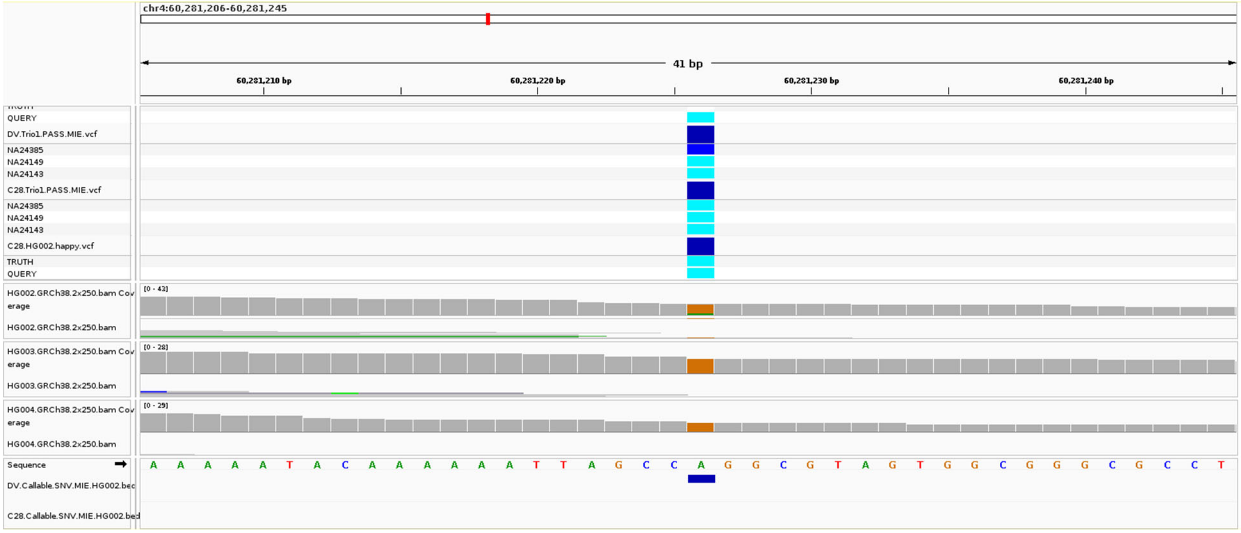

Figure S22. Discordant SNV found in a GIAB trio at Chr4:60218226

	HG002	HG003	HG004	Summary
GIAB Truth	1/1	./.	./.	
DV	0/1	1/1	1/1	Discordant due to FN in HG002
28	1/1	1/1	1/1	Correct

SUPPLEMENTAL METHODS

Note S3. Public Samples from SRA

Relevant samples are identified from NCBI SRA based on taxon ID, platform, layout, and sequencing strategy. For example:

```
https://www.ncbi.nlm.nih.gov/sra/?term=(txid9913[All
Fields])+AND+((("Illumina"[Platform]) AND "paired"[Layout]) AND
"wgs"[Strategy])
```

The RunInfo report is stored in a local database, and samples are selected by aggregating runs based on BioSampleID and selecting samples with at least 3-fold raw sequence coverage. SRA runs are downloaded using the SRA Toolkit for each group. The SRA Toolkit versions ranged from 2.9.1 to 2.11.0, as new data are downloaded periodically over time. (https://github.com/ncbi/sra-tools)

```
prefetch --progress --max-size 200G $Run
```

The prefetched data are then converted to fastq using either fastq-dump or fasterq-dump.

```
fastq-dump --split-e --outdir $CWD $Run
fasterq-dump -f -p -e $DumpThreads --split-3 --outdir $CWD $Run
```

The resulting fastq files are renamed to our UMAG nomenclature and compressed with pigz (https://zlib.net/pigz/).

Note S4. Sequence Data Processing

Data are processed in stages from FASTQ to final analysis-ready BCF following GATK Best Practices (Depristo et al. 2011; Van der Auwera and O'Conn) and implemented using various custom Perl scripts. Each stage is described below, and a representative command line is shown. As data have been continuously processed over time, starting in 2018, exact software versions have varied. However, the general workflow has been consistent, and only specific changes based on software version options have been varied.

Note S5. Sequence Quality Control

Low-quality bases and adapters are removed using trimmomatic (Bolger et al. 2014) (https://github.com/usadellab/Trimmomatic) Versions 0.36 to 0.39.

```
java -Djava.io.tmpdir=${cwd}/tmp -XX:+UseParallelGC \
-XX:ParallelGCThreads=2 -jar $Trimmomatic \
PE -phred${InputQV} \
-threads $Cpu_Trim \
-summary ${TrimOutBaseFile}.TRIM.SUMMARY \
$for_file $rev_file \
${TrimOutBaseFile}.1.P.fq ${TrimOutBaseFile}.1.U.fq \
${TrimOutBaseFile}.2.P.fq ${TrimOutBaseFile}.2.U.fq\
MINLEN:35 TOPHRED33 ILLUMINACLIP:${RefGenome}/${AdapterFile}:2:30:6:1:TRUE \
LEADING:20 TRAILING:20 SLIDINGWINDOW:3:15 AVGQUAL:20 MINLEN:35
```

Note S6. Bovine Reference Genome

The reference genome used is the same as that used by the 1000 Bull Genomes project (Hayes and Daetwyler 2018), ARS-UCD1.2_Btau5.0.1Y. Briefly, because the reference animal was female, the autosomes, X Chromosome, mitochondria, and unplaced contigs from GCA_002263795.2 (Rosen et al. 2020) were merged with the Y Chromosome from GCA_000003205.6 (Elsik et al. 2009) which is the sire of the reference animal.
Additional details are available here: https://bovinegenome.elsiklab.missouri.edu/downloads/ARS-UCD1.2

Note S7. Genome Alignment

FASTQ data passing QC were aligned to the reference genome initially with BWA MEM (https://github.com/lh3/bwa) and later with BWA MEM2 (https://github.com/bwa-mem2/bwa-mem2) after appropriate reference genome indexing. The appropriate read group tags are populated during alignment to facilitate downstream processing.

```
$bwa mem -M -t $Cpu_Align -R
'\@RG\\tID:${base_file}\\tSM:${animal_id}\\tLB:${lib}\\tPL:ILLUMINA' $BWA_ref
$for_file $rev_file >$bwa_output
```

Note S8. Sort/Merge/Mark Duplicates

The SAM files are sorted using SAMtools (https://github.com/samtools/), resulting in a list of sorted BAM files which are subsequently merged using picard (https://github.com/broadinstitute/picard) and duplicates marked using Picard. Crucially, MarkDuplicates is performed in a “read group aware” manner using the appropriate optical pixel distance for traditional flow cells (100) or patterned flow cells (2500) based on the source of the data. For samples that consisted of multiple sequencing libraries, the resulting library-specific BAM files are merged and indexed to produce a single BAM file using SAMtools.

```
$Samtools sort -m ${SortMem}G -\@ $Cpu_Samtools -o $sort_output -T
${sam_input}.TMP $sam_input

java -Djava.io.tmpdir=${cwd}/tmp -XX:+UseParallelGC \
-XX:ParallelGCThreads=2\
-Xmx20g -jar $Picard MergeSamFiles @in OUTPUT=${BAM_PREFIX}.${_}.merged.bam\
USE_THREADING=TRUE MERGE_SEQUENCE_DICTIONARIES=TRUE ASSUME_SORTED=TRUE\
VALIDATION_STRINGENCY=LENIENT TMP_DIR=${cwd}/tmp

java -Djava.io.tmpdir=${cwd}/tmp -XX:+UseParallelGC \
-XX:ParallelGCThreads=6 \
-Xmx50g -jar $Picard MarkDuplicates \
-INPUT ${BAM_PREFIX}.${_}.merged.bam -OUTPUT ${BAM_PREFIX}.${_}.bam \
-METRICS_FILE ${BAM_PREFIX}.${_}.DUP.METRICS -MAX_RECORDS_IN_RAM 5000000 \
-MAX_FILE_HANDLES_FOR_READ_ENDS_MAP 1000 -ASSUME_SORTED TRUE \
-VALIDATION_STRINGENCY LENIENT -TMP_DIR ${cwd}/tmp \
-OPTICAL_DUPLICATE_PIXEL_DISTANCE ${Pixels} -COMPRESSION_LEVEL 0

$Samtools merge -\@ $Cpu_Samtools -p -f -b ${BAM_PREFIX}_MERGE_files.txt
${BAM_PREFIX}.bam
$Samtools index -\@ 8 ${BAM_PREFIX}.bam
```

Note S9. Indel Realignment

While indel realignment was removed from the Best Practices, we have elected to retain it as part of our standard processing to facilitate other analyses not performed in the current work. We process each chromosome independently to generate indel targets, realign, and then merge all chromosomes back into a single indexed BAM file. The GATK version used remained constant (3.8-1-0-gf15c1c3ef).

```
java -Djava.io.tmpdir=${cwd}/tmp -XX:+UseParallelGC \
-XX:ParallelGCThreads=2 \
-Xmx8g -jar $GATK -nt $Cpu_RTC -T RealignerTargetCreator \
-R ${RefGenome}/${ref}.fa -L $_ -I ${BAM_PREFIX}.bam \
-o ${BAM_PREFIX}.${_}.forIndelRealigner.intervals

java -Djava.io.tmpdir=${cwd}/tmp -XX:+UseParallelGC \
-XX:ParallelGCThreads=2 \
-Xmx${JavaMem}g -jar $GATK -R ${RefGenome}/${ref}.fa -I ${BAM_PREFIX}.bam \
-T IndelRealigner \
-targetIntervals ${BAM_PREFIX}.${_}.forIndelRealigner.intervals \
-L $_ -o ${BAM_PREFIX}.${_}.${BAM_SUFFIX}.bam --maxReadsInMemory 300000

$Samtools merge -\@ $Cpu_Samtools -f -c -p \
-b ${BAM_PREFIX}_MergeRealignedFiles.list ${BAM_PREFIX}.${BAM_SUFFIX}.bam
$Samtools index -\@ 8 ${BAM_PREFIX}.${BAM_SUFFIX}.bam
```

Note S10. Base Quality Score Recalibration (BQSR)

Sequencing data were generated over many years and many platforms, thus requiring base quality recalibration. The initial known variant file used was from the 1000 Bull Project and has been subsequently modified over time to account for additional data. Briefly, the initial known variant file was constructed based on remapping variants from dbSNP build 150, containing approximately 104 million variants, onto the new UCD1.2_Btau5.0.1Y reference genome. Supplementary unique variants were added from 1000 Bull Project Runs7 and Run8 based on Taurus and Indicus genomes. Additionally, variants from closely related species such as Bison, Gaur, Banteng, and Yak (outgroups) were added. The final file contains 174.3 M SNPs and 22.9 M indels (UMAG_9913_BQSR_ARS1.2_V4.vcf.gz). Further testing revealed that base qualities for these outgroup species were over-corrected, likely due to their genetic distance from the Hereford (*Bos taurus*) reference genome. Setting the following parameters alleviated the over-correction while not adversely affecting *Bos taurus* or *Bos indicus* samples:

```
--bqsrBAQGapOpenPenalty 45
--deletions_default_quality 45
--insertions_default_quality 45
```

Optimal BQSR is obtained by building a recalibration model using the entire genome and all available information; however, this requires lengthy run times. Extensive testing was performed to evaluate using smaller intervals to build the model. Because the quality of the recalibration model depends on the amount of data provided, we reasoned that a sufficient model could be obtained using smaller genomic intervals based on the total amount of sequencing data available for a sample. Given that the Bos taurus genome is approximately 3 Gb in size and contains 29 autosomes plus the X Chromosome, we developed “target” intervals of size 5 Mb, 10 Mb, and 20 Mb for each of the 30 chromosomes starting at position 1 Mb on each chromosome. We developed “testing” intervals of the same size starting at position 20 Mb. The “target” intervals are used to build the recalibration model used for BQSR. The “testing” intervals are used to generate a new recalibration model after the data have been recalibrated and to evaluate the overall quality before and after recalibration. Because the on-disk size of a BAM file is proportional to the total amount of sequence data contained within the file, we determine the interval size to use for BQSR based on the BAM file size where files <10 GB use the entire genome, <35 GB use 20 Mb, <70 GB use 10 Mb and >70 GB use 5 Mb target/testing intervals. This results in similar amounts of data used for model generation/evaluation for each sample while significantly decreasing run time. Through extensive testing (data not shown), we determined that the reduced intervals based on total data produced results similar to those of using the entire genome. Importantly, because our target and testing intervals are distinct, a well-

calibrated testing model indicates that the model used for the entire genome was also well-calibrated. GATK BaseRecalibrator and PrintReads used GATK version 3.8-1-0-gf15c1c3ef. PrintReads is run in parallel by chromosome, then merged and indexed. GATK version 4.1.9.0 was used for AnalyzeCovariate plots.

```
java -Djava.io.tmpdir=${cwd}/tmp -XX:+UseParallelGC \
-XX:ParallelGCThreads=2 \
-Xmx20g -jar $GATK -nct $Cpu_BQSR -T BaseRecalibrator \
-R ${RefGenome}/${ref}.fa -I ${BAM_PREFIX}.${BAM_SUFFIX}.bam \
-L ${RefSNP}/${snp_dir}/BQSR_${BqsrSize}MB_target.interval_list \
-knownSites ${RefSNP}/${snp_dir}/${KnownSites} \
--bqsrBAQGapOpenPenalty $bqsrBAQGOP --deletions_default_quality $indelQUAL \
--insertions_default_quality $indelQUAL \
-o ${BAM_PREFIX}.recalibration_report.grp \
-U ALLOW_N_CIGAR_READS --quantizing_levels 24

java -Djava.io.tmpdir=${cwd}/tmp -XX:+UseParallelGC \
-XX:ParallelGCThreads=2 \
-Xmx12g -jar $GATK -nct $Cpu_PR -T PrintReads -R ${RefGenome}/${ref}.fa \
-L $_ -I ${BAM_PREFIX}.${BAM_SUFFIX}.bam \
-BQSR ${BAM_PREFIX}.recalibration_report.grp \
-o ${BAM_PREFIX}.${_}.${BAM_SUFFIX}.recalibrated.bam -U ALLOW_N_CIGAR_READS

$Samtools merge -\@ $Cpu_Samtools -l 9 -f -c -p -b \
${BAM_PREFIX}_MergeRealignedRecalibratedFiles.list \
${BAM_PREFIX}.$BAM_SUFFIX.bam

$Samtools index -\@ 4 ${BAM_PREFIX}.${BAM_SUFFIX}.bam

java -Djava.io.tmpdir=${cwd}/tmp -XX:+UseParallelGC \
-XX:ParallelGCThreads=2 \
-Xmx10g -jar $GATK4 AnalyzeCovariates \
-before ${BAM_PREFIX}.recalibration_report.grp \
-after ${BAM_PREFIX}.recalibration_report2.grp -plots ${BAM_PREFIX}.BQSR3.pdf
```

Note S11. Summary Metrics
We collect summary metrics using GATK DepthOfCoverage and Picard CollectMultipleMetrics. These outputs are reformatted and stored within our database for downstream QC and summary metrics.

DepthOfCoverage
```
java -Djava.io.tmpdir=${cwd}/tmp -XX:+UseParallelGC \
-XX:ParallelGCThreads=2 \
-Xmx10g -jar $GATK -nt $Cpu_DOC -R ${RefGenome}/${ref}.fa \
-I ${BAM_PREFIX}.${BAM_SUFFIX}.bam -T DepthOfCoverage -L $_ \
-o ${BAM_PREFIX}.${_}.${BAM_SUFFIX}.bam.coverage -omitBaseOutput \
-omitIntervals --omitLocusTable -ct 5 -ct 10 -ct 15 -ct 20 -ct 25 \
-ct 30 -ct 40 -ct 50 -ct 80 -ct 90 -ct 100 -ct 150 --minBaseQuality 15 \
--minMappingQuality 30 --start 1 --stop 1000 --nBins 999 -dt NONE \
-U ALLOW_N_CIGAR_READS
```

CollectMultipleMetrics
```
java -Djava.io.tmpdir=${cwd}/tmp -XX:+UseParallelGC \
-XX:ParallelGCThreads=2 \
-Xmx10g -jar $Picard CollectMultipleMetrics \
-INPUT ${BAM_PREFIX}.${BAM_SUFFIX}.bam -OUTPUT ${BAM_PREFIX} \
-METRIC_ACCUMULATION_LEVEL LIBRARY \
-REFERENCE_SEQUENCE ${RefGenome}/${ref}.fa\
-PROGRAM CollectAlignmentSummaryMetrics \
-PROGRAM CollectInsertSizeMetrics \
-STOP_AFTER $CollectMetricsNumReads \
-TMP_DIR ${cwd}/tmp -ASSUME_SORTED TRUE
```

Note S12. HaplotypeCaller & CallableRegions
GATK HaplotypeCaller is used to generate gVCF files for each sample and each chromosome in parallel. Based on initial estimates from the Bovine HapMap project (Gibbs et al. 2009), we set the `--heterozygosity` parameter to 0.0015. We also generate CallableRegions for each chromosome to facilitate downstream applications.

```
java -Djava.io.tmpdir=${cwd}/tmp -XX:+UseParallelGC \
-XX:ParallelGCThreads=2 \
-Xmx15g -jar $GATK -nct $Cpu_HC -ERC GVCF -T HaplotypeCaller \
-R ${RefGenome}/${ref}.fa -L $_ -I ${BAM_PREFIX}.${BAM_SUFFIX}.bam \
-o ${BAM_PREFIX}.${_}.g.vcf.gz --heterozygosity $Heterozygosity \
--pcr_indel_model NONE --useNewAFCalculator
```

```
java -Djava.io.tmpdir=${cwd}/tmp -XX:+UseParallelGC \
-XX:ParallelGCThreads=2 \
-Xmx15g -jar $GATK -T CallableLoci -R ${RefGenome}/${ref}.fa -L $_ \
-I ${BAM_PREFIX}.${BAM_SUFFIX}.bam \
-summary ${BAM_PREFIX}.CallableLoci.${_}.summary.txt \
-o ${BAM_PREFIX}.CallableLoci.${_}.bed
```

Note S13. Joint Genotype Calling
Initially, 5,612 genomes were available for joint genotype calling. The gVCF files for each chromosome were combined using groups of 100 samples, resulting in 57 gVCF files per chromosome. These combined gVCF files are then passed as a list to GenotypeGVCs for joint genotype calling. In order to reduce total run time, chromosomes are genotyped in 10 Mb chunks in parallel and then concatenated/merged back into a single vcf per chromosome and finally indexed using BCFtools (GATK: v3.8-1-0-gf15c1c3ef, BCFtools: v1.14).

```
java -Djava.io.tmpdir=$cwd/tmp -XX:ParallelGCThreads=2 -Xmx20g \
-jar $GATK \
-T CombineGVCFs -R ${RefGenome}/${ref}.fa -V ${InputFile}.${i}.${Chr}.list \
-o ${cwd}/gvcf/${Cohort}.${i}.${Chr}.g.vcf.gz

java -Djava.io.tmpdir=$tmpdir -XX:+UseParallelGC \
-XX:ParallelGCThreads=2 \
-Xmx${Mem_Node}g -jar $GATK -nt $Cpu_Node -T GenotypeGVCFs \
-R ${RefGenome}/${ref}.fa -V ${Cohort}.${Chr}.list \
-L ${Chr}:${StartPosition}-${EndPosition} \
-o $outdir/${Cohort}.${Chr}.${IntervalChunk}.vcf.gz \
--heterozygosity ${Heterozygosity} --useNewAFCalculator \
--standard_min_confidence_threshold_for_calling 5

bcftools concat --naive --file-list CAT.${c}.list -Oz9 -o ${COHORT}.$c.vcf.gz
bcftools index --tbi ${COHORT}.$c.vcf.gz
```

We create SitesOnly files for downstream use with Variant Quality Score Recalibration using GATK 4.2.6.1.

```
$GatkBinary MakeSitesOnlyVcf --COMPRESSION_LEVEL 9 \
-I vcf/${YYMMDD}.${CNAME}.$c.vcf.gz \
-O vcf/SitesOnly/${YYMMDD}.${CNAME}.$c.vcf.gz
```

Note S14. Variant Quality Score Recalibration (VQSR)
The effectiveness of VQSR is dependent on the overall quality and quantity of the variants used as truth sets, which are generally lacking for non-model organisms. Additionally, settings for appropriate priors for each truth set should be optimized in addition to evaluating the annotations used for VQSR. In order to develop these resources, we developed a method to evaluate many parameters in parallel and iterate until the final set of acceptable values are identified. The general principle is that we brute-force test all combinations of parameters and evaluate the number of variants in the resulting tranches at each iteration. We choose an “optimal” set of parameters at each iteration, recalibrate the SitesOnly files, evaluate Mendelian discordance in trios, and select PASS variants to generate a new “truth” set. Here, “optimal” is subjective and is based on examining the behavior of the number of variants in each tranche as well as the clustering of variants in the summary PDF files where more tightly clusters variants separating known/novel, filtered/retained, and positive/negative are indicative of better recalibration. Quantitatively, we count Mendelian errors by tranche in a series of trios where a lower error rate indicates better-performing models.

Tranches: To evaluate the finer-scale impact of parameter changes, we use 21 total tranches with ten tranches between 90-99 and ten tranches between 99.0 and 100 for both SNP and indels, evaluated independently. PASS variants are set as tranche 90 for evaluation purposes.

Truth Sets: The final set of variants used for VQSR after seven iterations is shown in the table below. Each represents unique variants, starting with the most confident variants. The HD_F250 are loci contained on the Illumina Bovine HD (Illumina, San Diego, CA) and Geneseek GGPF250 (Rowan et al. 2019) assays that were filtered on variant call rate and Mendelian error rate metrics from >10,000 samples genotyped on each assay. The remaining SNP truth/training sets were selected based on the criteria below:

```
UMAG1_SNP_TRUTH1U: tranche PASS, 0.05<AF<1.0, SnpGap 10, No trio errors
UMAG1_SNP_TRUTH2U: tranche PASS, 0.01<AF<0.05, SnpGap 10, No trio errors
UMAG1_SNP_TRUTH3U: tranche PASS, 0.001<AF<0.01, SnpGap 10, No trio errors
UMAG1_SNP_train1U: tranche 90.0-98.0, 0.05<AF<1.0, SnpGap 10, No trio errors
UMAG1_SNP_train2U: tranche 90.0-98.0, 0.01<AF<0.05, SnpGap 10, No trio errors
UMAG1_SNP_train3U: tranche 90.0-98.0, 0.001<AF<0.01, SnpGap 10, No trio
errors
```

The indel truth/training sets used the same criteria except set IndelGap to 10.

VQSR truth set iterations.

Resource Name	*Known*	*Training*	*Truth*	*Prior*	*NumVariants*
HD_F250	**false**	**true**	**true**	**15**	**862,012**
AffyBOS1U	false	true	true	15	316,018
UMAG1_SNP_truth1U	false	true	true	12	17,140,772
UMAG1_SNP_truth2U	false	true	true	12	12,865,155
UMAG1_SNP_truth3U	false	true	true	12	12,303,684
UMAG1_SNP_train1U	false	true	true	10	4,194,140
UMAG1_SNP_train2U	false	true	true	10	2,930,294
UMAG1_SNP_train3U	false	true	true	10	19,590,532
UMAG1_BQSRV4	true	false	false	7	174,362,380
UMAG1_INDEL_truth1U	**false**	**true**	**true**	**12**	**2,651,881**
UMAG1_INDEL_truth2U	false	true	true	12	1,517,732
UMAG1_INDEL_truth3U	false	true	true	12	1,375,626
UMAG1_INDEL_train1U	false	true	true	10	372,192
UMAG1_INDEL_train2U	false	true	true	10	319,137
UMAG1_INDEL_train3U	false	true	true	10	1,251,861
UMAG1_BQSRV4	true	false	false	2	174,362,380

Parameters Evaluated: Below is a summary of the parameters and values tested and their defaults and optimal values. Each variable was run combinatorically such that only one was varied while holding all others constant. We then evaluate the impact of each variable in isolation and comparison to the others. This results in approximately 135-150 VQSR runs per iteration. After the first couple of rounds of iterations, it was determined that `--max-gaussians` of 9 or 10 and `--max-negative-gaussians` of 5 or 6 performed poorly and were abandoned in later iterations. As a general observation, we found the `--bad-lod-score-cutoff` to have the most significant impact. As part of the pipeline, we evaluate the distribution of the number of variants in the positive and negative models versus the VQSLOD scores for each run (**Figure S23**).

Early in the testing, we noticed that the variants included in the negative model produced a long-tailed distribution. The variants in the long tail represent the loci that consistently produce very negative LOD scores, indicating poor quality. However, most of the density is contained near the bad-lod-score-cutoff. This parameter's value dramatically impacts the model's ability to differentiate “good” from “bad” variants. For example, extreme negative values for this threshold result in too few variants to model variation effectively. Conversely, the default value of -5 includes far too many variants, making it difficult to differentiate “good” from “bad” variants. These variables are interdependent and dataset-specific, making truly optimal value selection arduous (**Figures S24, S25**). The GATK documentation states, “VQSR is probably the hardest part of the Best Practices to get right.” We have performed similar analyses with dogs, pigs, and honeybees; each resulted in different optimal values.

```
max-gaussians: 4,5,6,7,8,9,10, [default 8], Optimal SNP=4, INDEL=4
max-negative-gaussians: 2,3,4,5,6 [default 2], Optimal SNP=4, INDEL=2
minimum-bad-variants: 1000 [default 1000]
maximum-training-variants: 10000000 [default 2500000]
bad-lod-score-cutoff: -5,-7,-9,-11,-13,-15,-17,-19,-21,-23,-25 [default -5] ,
Optimal SNP=-25, INDEL=-25
SNPS Annotations: '-an QD -an ReadPosRankSum -an FS -an MQ -an SOR -an DP'
INDEL Annotations: '-an QD -an ReadPosRankSum -an FS -an SOR -an DP'
```

```
java -Djava.io.tmpdir=tmp_${LABEL} -XX:ParallelGCThreads=2 -Xmx${JMEM}g \
-jar $GATKJAR \
VariantRecalibrator \
-nt $Cpu_Node \
-R $REF \
-V $SitesOnlyList \
-tranche 100.0 -tranche 99.90 -tranche 99.80 -tranche 99.70 -tranche 99.60 \
-tranche 99.50 -tranche 99.40 -tranche 99.30 -tranche 99.20 -tranche 99.10 \
-tranche 99.0 -tranche 98.0 -tranche 97.0 -tranche 96.0 -tranche 95.0 \
-tranche 94.0 -tranche 93.0 -tranche 92.0 -tranche 91.0 -tranche 90.0 \
--mode $MODE \
--seconds-between-progress-updates 300 \
--trust-all-polymorphic \
$Annotations \
-resource:\
${key},known=${Known},training=${Training},truth=${Truth},prior=${Prior}
$KVPath \
--max-gaussians $MG \
--max-negative-gaussians $MNG \
--minimum-bad-variants $NUMBAD \
--maximum-training-variants $NUMTRAIN \
--max-iterations $MaxIterations \
--max-attempts 5 \
--bad-lod-score-cutoff $BADLOD \
--output ${LABEL}.recal \
--tranches-file ${LABEL}.tranches \
--rscript-file ${LABEL}.R \
--output-model ${LABEL}.model \
--tmp-dir tmp_${LABEL}
```

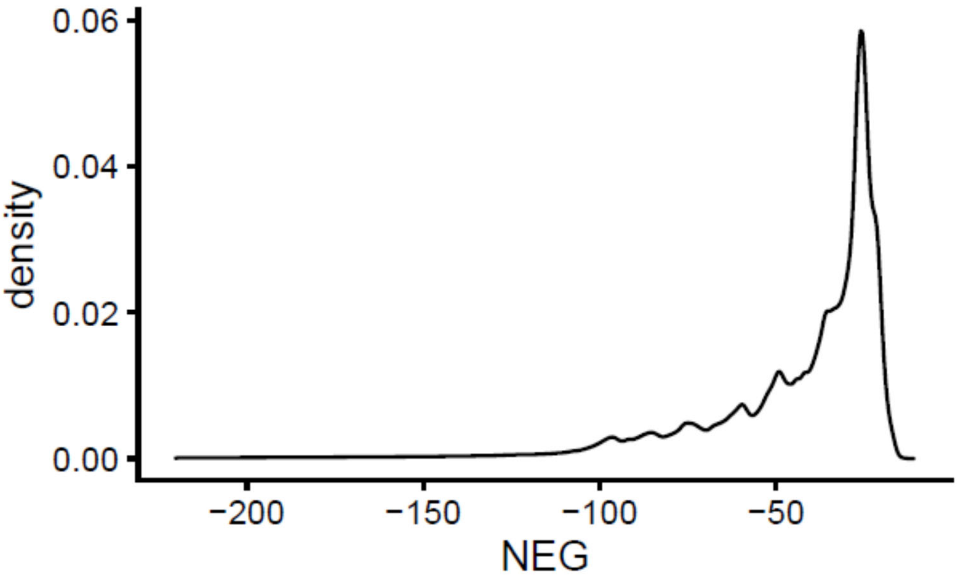

Figure S23. VQSR negative model
Example of the distribution of the number of loci in the negative model used for VQSR in the optimal SNP model with `--bad-lod-score-cutoff` = -25.

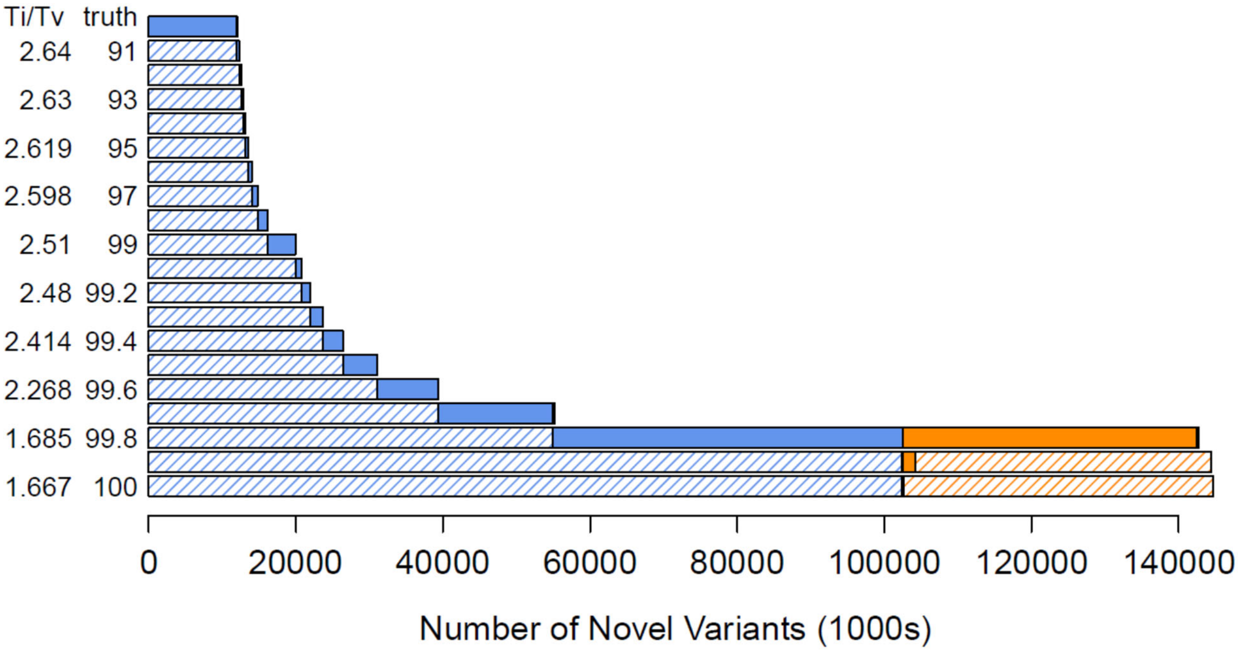

Figure S24. Tranche plot for the optimal SNP model parameters.

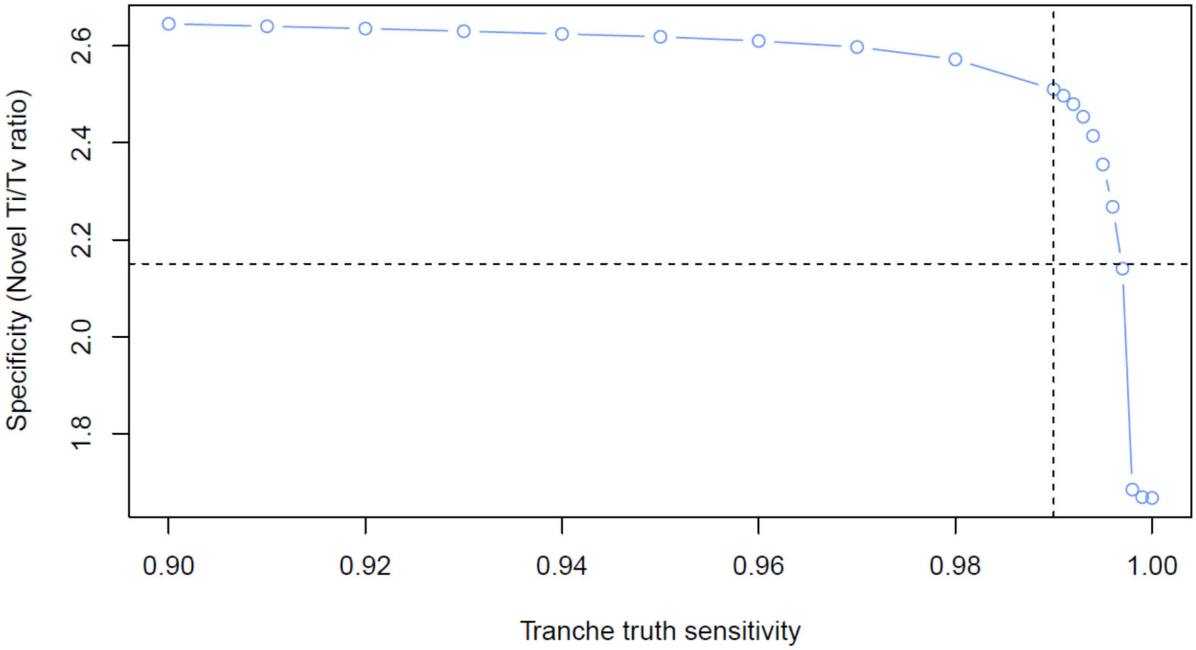

Figure S25. Tranche sensitivity plot for the optimal SNP model parameters.

Note S15. VQSR Mendelian Error Evaluation

A total of 15 trios were selected, with each trio member sequenced at a minimum of 10× coverage. We evaluated the optimal VQSR parameters and identified an appropriate tranche sensitivity value with these trios based on the Mendelian Error rate for Chromosome 25. For each of the 21 tranches, we calculate the Mendelian error rate after recalibration in the 15 trios for only the loci in the specific tranche. This approach allows us to evaluate the impact of increasingly including lower-quality variants. **Figures S26 and S27** demonstrate an increase in SNP Mendelian error rate as sensitivity increases, with the error rate increasing notably at tranche 99.7. **Figures S28 and S29** show the same trend for indels but, as expected, show a much higher baseline error rate with a noticeable increase at tranche 99.8. Based on Mendelian error profiles and other factors, a final tranche threshold was set as PASS (SNPs = 99.7, indels = 99.8). Lastly, we contrast the error rates from our optimized VQSR against the GATK defaults and the 1kBulls Run9 in **Figure S30**.

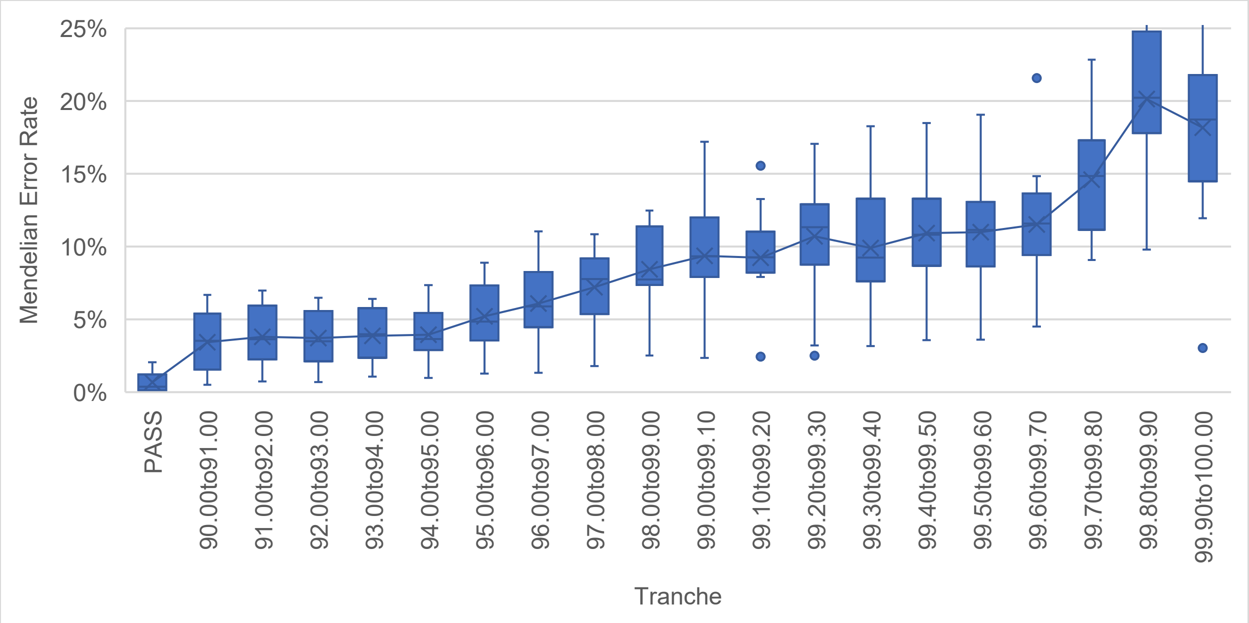

Figure S26. Tranche-specific Mendelian error rate for SNP variants.
The x-axis represents the error rate for the loci with each tranche. We observe a noticeable increase in error rate after tranche 99.7.

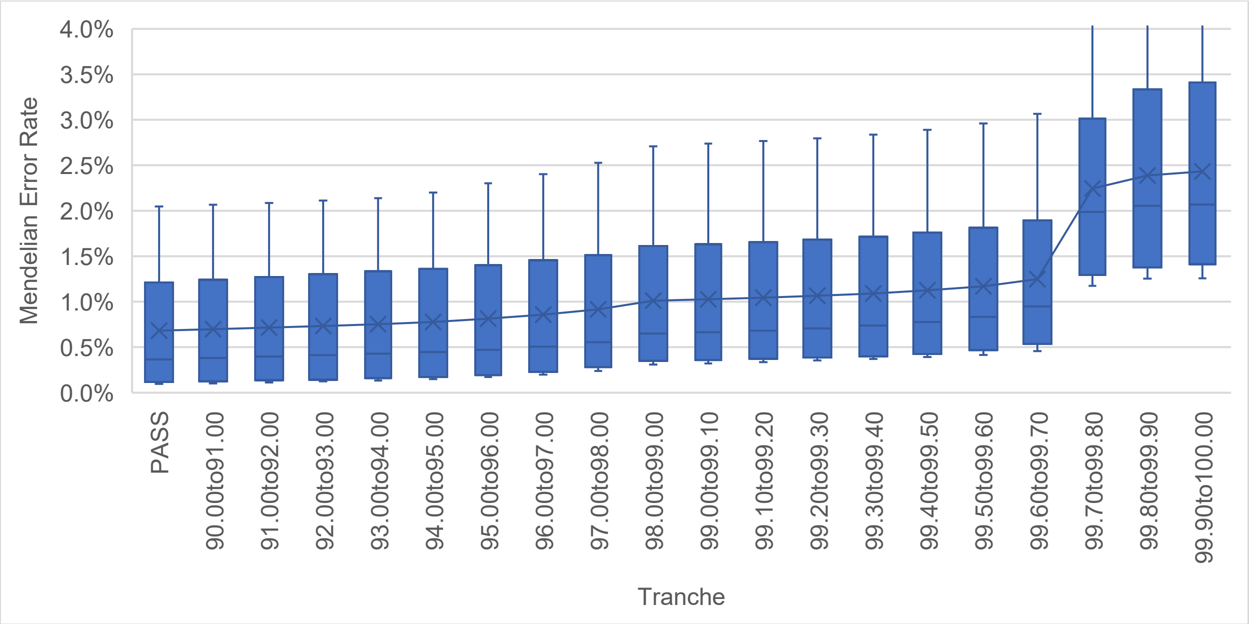

Figure S27. Cumulative Mendelian error rate for SNP variants.
The x-axis represents the accumulating error rate, where each tranche includes loci at lower sensitivities. There is a steady increase in error rate until tranche 99.7, where the remaining 0.3% of variants more than double the error rate.

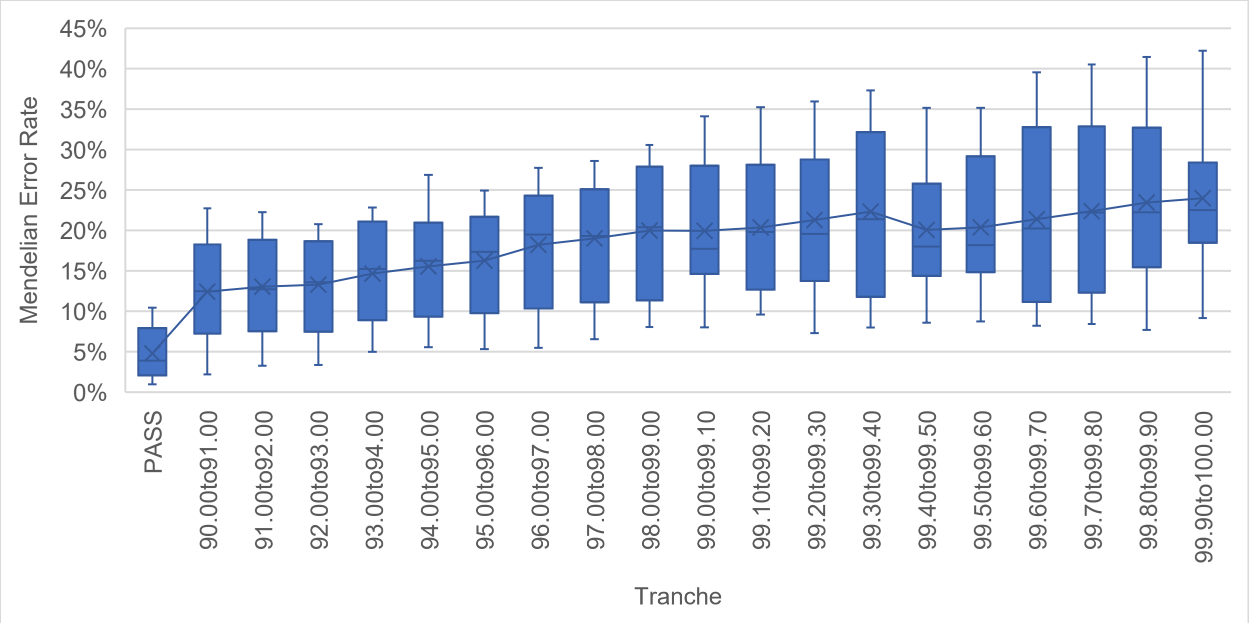

Figure S28. Tranche-specific Mendelian error rate for indel variants.
The x-axis represents the error rate for the loci within each tranche. We observe a noticeable increase in error rate at tranche 91.

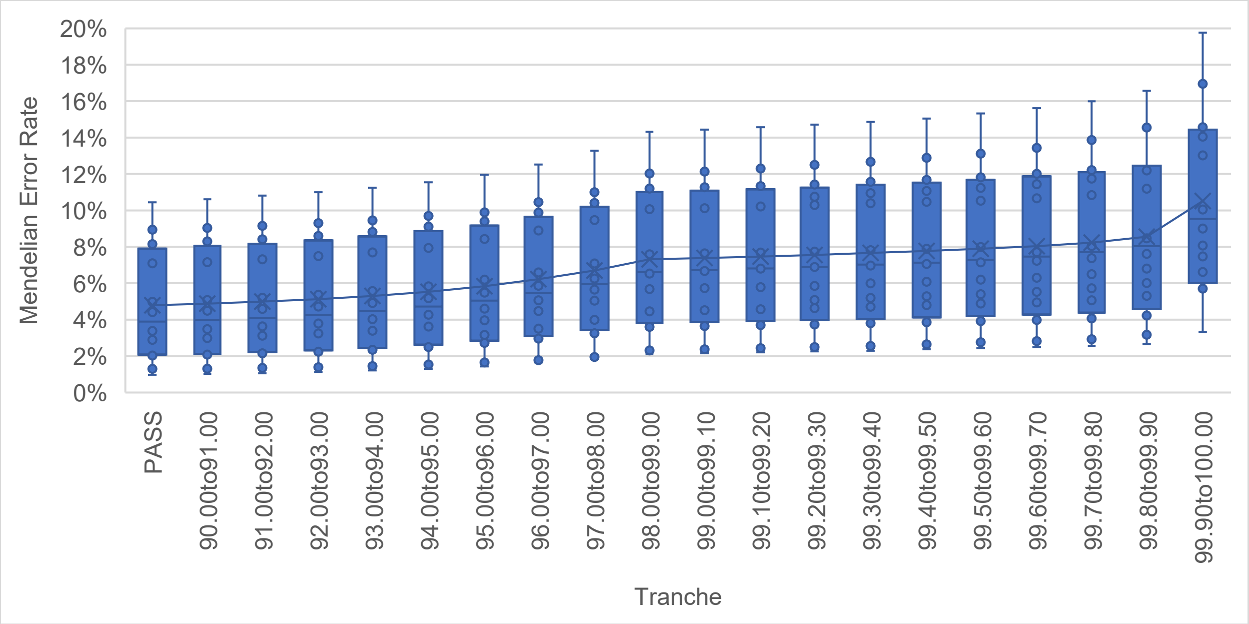

Figure S29. Cumulative Mendelian error rate for indel variants.
The x-axis represents the accumulating error rate, where each tranche includes loci at lower sensitivities. There is a steady increase in error rate until tranche 99.9.

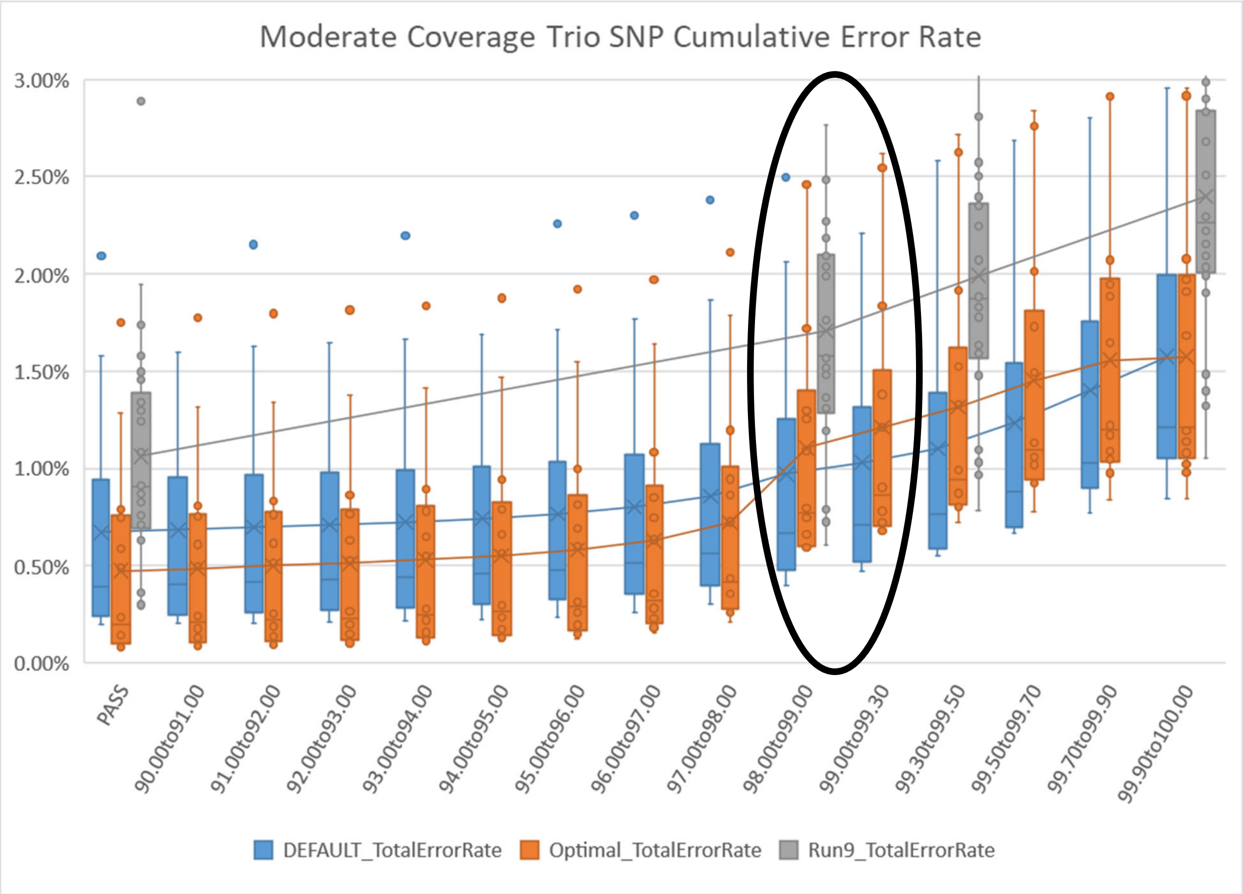

Figure S30. Comparing VQSR approaches.
Box and whisker plots showing the Mendelian discordance rate for 13 cattle trios using the default (blue), the optimal (orange), and the 1kBulls Run9 (grey) VQSR parameter values. The optimal parameter set indicated a relatively flat and low error rate up to the tranche value of 97, with an elevated error rate after tranche 99.0 (circled).

Note S16. ApplyVQSR
After VQSR parameter evaluation, a final tranche threshold was set as PASS (SNPs = 99.7, indels = 99.8) and used the respective optimal models for each chromosome in parallel with GATK (4.3.0.0).

```
java -Djava.io.tmpdir=tmp_SNP_${c} -XX:ParallelGCThreads=2 \
-Xmx${Jmem}g \
-jar $GatkJar ApplyVQSR -R $Ref \
-V ${YYMMDD}_${COHORT}/vcf/${YYMMDD}.${COHORT}.$c.vcf.gz \
-O ${COHORT}.SNP.${c}.vcf.gz \
-L $c \
--truth-sensitivity-filter-level $SnpSensitivityTranche \
--tranches-file ${OptimalSnpLabel}.tranches \
--recal-file ${OptimalSnpLabel}.recal \
--tmp-dir tmp_SNP_${c} \
--seconds-between-progress-updates 120 \
-mode SNP

java -Djava.io.tmpdir=tmp_INDEL_${c} -XX:ParallelGCThreads=2 \
-Xmx${Jmem}g \
-jar $GatkJar ApplyVQSR -R $Ref \
-V ${COHORT}.SNP.${c}.vcf.gz \
-O ${COHORT}.SNP.INDEL.${c}.vcf.gz \
-L $c \
--truth-sensitivity-filter-level $IndelSensitivityTranche \
--tranches-file ${OptimalIndelLabel}.tranches \
--recal-file ${VqsrDirIndel}/${OptimalIndelLabel}.recal \
--tmp-dir tmp_INDEL_${c} \
--seconds-between-progress-updates 120 \
-mode INDEL
```

Note S17. Initial Quality Control (QC)
We examined the initial call set using BCFtools (v1.16) smpl-stats plugin to identify low-quality samples to exclude. As expected, sample coverage was the primary determinant of the number of PASS genotypes per sample (**Figure S31**). Samples with a call rate <0.85 were flagged to exclude (N=47). Samples with a call rate between 0.85–0.90 were retained at this stage because metrics such as Ts/Tv, number of singletons, and heterozygous to homozygous ratio were all within normal ranges (**Figure S32**).

```
bcftools +smpl-stats -i 'FILTER=\"PASS\"' -o tmp.$c.PASS.tsv
${TaxonID}.UMAG${UmagVersion}.ENSEMBL${AnnotationVersion}.$c.bcf
bcftools view --threads $CPU_view -S ^${SamplesToRemove} -Ou \
$InputDir/${TaxonID}.UMAG${UmagVersion}.ENSEMBL${AnnotationVersion}.$c.bcf |\
bcftools --trim-alt-alleles -e 'ALT=\".\" -Ob -o \
${OutputDir}/${TaxonID}.UMAG${UmagVersion}.ENSEMBL${AnnotationVersion}.$c.bcf
bcftools index --threads $CPU_index \
${OutputDir}/${TaxonID}.UMAG${UmagVersion}.ENSEMBL${AnnotationVersion}.$c.bcf
```

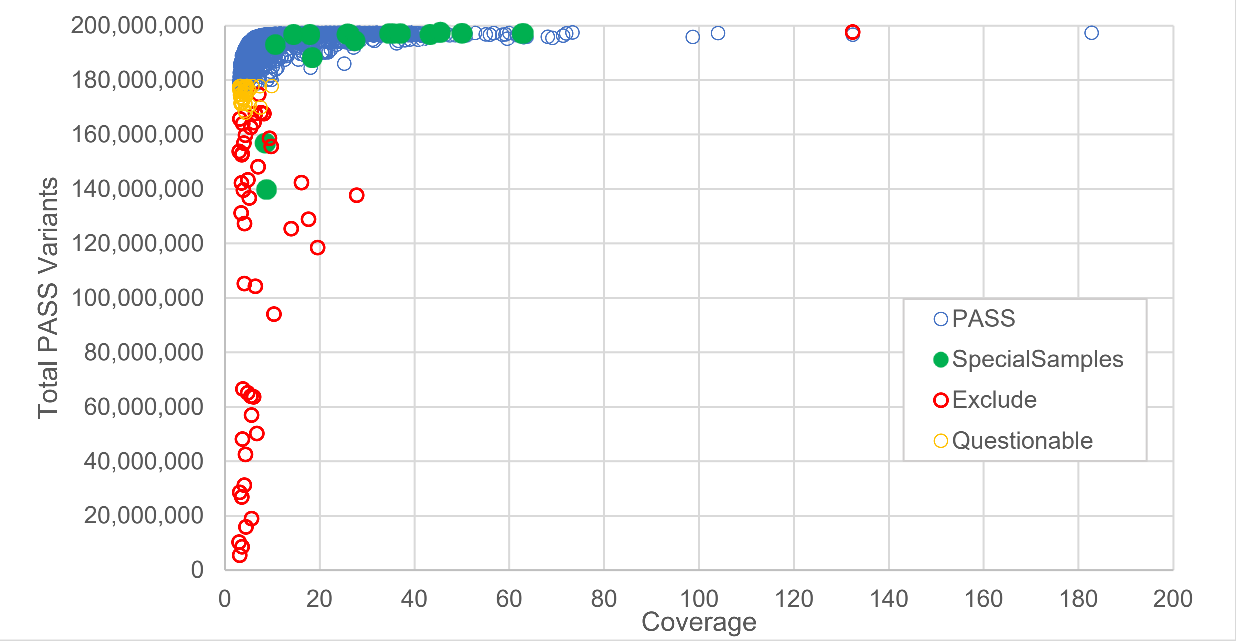

Figure S31. Initial QC of samples demonstrating the impact of genome coverage.
Samples with a total call rate <0.85 were flagged for exclusion (red). Further testing revealed an outlier (132x, call rate =99.99%) was a misidentified duplicate matching the reference genome included previously under a different UMAG lab ID. The "special samples" (green) represent samples used to generate alternate bovine assemblies and three ancient bovid samples.

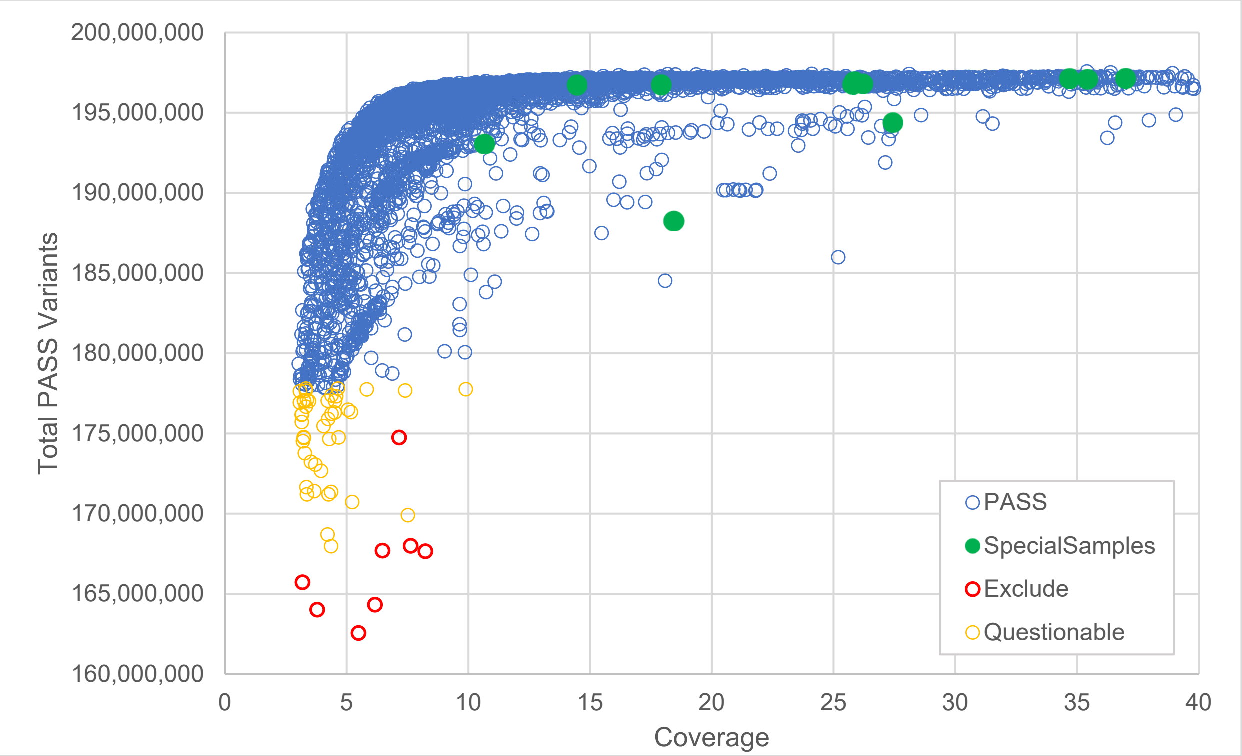

Figure S32. Initial QC of samples with axes truncated.
The samples labeled as "questionable" have a call rate between 0.85 and 0.90.

Note S18. Training Sample Criteria and Phases.
Cattle genomics data frequently includes families. While multiple offspring can occur per generation in cattle, they typically result via artificial insemination using one sire for several dams rather than multiple full siblings in mosquitos. Additionally, published cattle sequencing data includes pedigrees across generations, offering a larger sample size than the human GIAB trios. A copy of pedigree information for all bovine samples included in this study is available on Zenodo DOI: 10.5281/zenodo.15482484 (metadata.csv).

Phases 1 and 2: Consists of 18 training iterations (2 parents per trio × 9 cattle trios) across 26 unique genomes. These genomes represent six purebred trios and three crossbred trios. Five trios had increased similarity to the Hereford reference genome (ARS-UCD1.2_Btau5.0.1Y), while the remaining four trios represent more divergent breeds relative to the reference. These samples were randomized to identify any trio-specific characteristics that enable gains in training performance.

Phase 3: Consists of 4 training iterations (2 parents per trio × 2 Bison trios) across five unique genomes, as the offspring shared a sire. Bison samples were included to explore the impact of outgroup species upon training.

Phase 4: Consists of 6 training iterations (2 parents per trio × 3 real F1-hybrid trios) across nine unique genomes. The training was extended for these samples to determine if higher-quality truth labels could meaningfully impact performance.

Phase 5: Consists of 2 training iterations (2 parents per trio × 1 synthetic F1-hybrid trios) across one unique genome – a SynDip replicate of the Angus-Brahman trio from Phase 4 using the original short-read WGS for the parents, but synthetic reads for the offspring. A single synthetic trio was used to reserve the remaining two trios for model testing. This phase assessed if synthetic truth labels could be substituted for GIAB-quality truth labels.

Note S19. Testing genomes remain independent.

Phases 1 – 3: Consists of the same 19 bovine samples across all three phases. While training was performed for these phases, DeepVariant has never seen any labeled examples from the six F1-hybrid offspring, either real or synthetic. Thus, we used the 13 unrelated samples and the 6 F1-hybrid offspring to assess performance and track generalizability over the first 22 iterations with TrioTrain.

Phases 4 and 5: Consists of the 13 unrelated samples while incrementally removing one of the F1-hybrid samples once the first iteration begins. While training was performed for these phases, as soon as DeepVariant saw labels from an F1-hybrid trio, the offspring was never used for model testing again. By eliminating these samples from our testing cohort iteratively, we minimized data bleed because samples are never used for model selection and performance testing.

Note S20. Creating truth sets for DeepVariant.

After cohort QC, we generated truth sets based on the UMAG1 cohort using GATK-derived genotypes. The regions files produced by GATK (v3.8-1-0-gf15c1c3ef), followed by parsing per-sample CallableLoci to extract only PASS regions for downstream analyses; see Zenodo DOI: 10.5281/zenodo.15482484 . Using BCFtools (v1.14), we extract trio and testing samples from the full BCF (all variants); see European Nucleotide Archive (ENA) accession number PRJEB86883) and write them into 17 trio-specific files and a separate file containing the testing individuals. These VCF files are extracted per chromosome, and then individual chromosomes are concatenated and indexed to produce a single BCF file per trio.

```
bcftools +split --groups-file Group.txt -Ob -o $c
9913.UMAG1.ENSEMBL106.$c.bcf

bcftools concat --file-list Trio${t}.merge.list -Ob -o RAW.Trio${t}.bcf

bcftools index RAW.Trio${t}.bcf
```

Mendelian errors in the raw BCF file indicate poor-quality genotypes within one or more of the members of a trio. We removed Mendelian errors using BCFtools (v1.14) to produce trio-specific BCF files with no errors

```
bcftools +mendelian --rules-file $RulesFile RAW.Trio${t}.bcf \
-Ob -o CLEAN.RAW.Trio${t}.bcf -m d --trio-file Trio${t}.ID.txt

bcftools index CLEAN.RAW.Trio${t}.bcf
```

A third set of files is created for training using BCFtools (v1.14) individually. We only extract variants within the per-sample callable regions for the training samples. We exclude all sites where the individual is homozygous for the reference allele, has a missing genotype, or contains a spanning deletion (GT=*). Note that the previous removal of Mendelian errors sets the genotype at the site to missing, and these sites will be excluded at this step. During initial development, we also extracted a series of files that restricted the genotypes based on genotype quality (GQ=10,13,20,30) to evaluate the impact of removing lower-quality genotypes on training.

```
bcftools view --samples $sample --regions-file $sample.callable.bed -Ou
CLEAN.RAW.Trio${t}.bcf | \
bcftools view --exclude 'GT=\"RR\" | GT=\"mis\" | ALT=\"*\"' -Oz -o
$sample.vcf.gz

bcftools index --tbi $sample.vcf.gz
```

A population VCF was created containing all PASS variants to use the `--use_allele_frequency` option of DeepVariant. The allele frequencies are obtained from the cohort call set based on 5,512 samples, representing our best estimate of population allele frequencies. The resulting file contained 141,595,864 SNPs and 18,246,855 indels.

```
bcftools view -G -f PASS --exclude 'ALT=\"*\"' -Oz -o POP.$i.vcf.gz \
9913.UMAG1.ENSEMBL106.$i.bcf

bcftools concat --file-list POP.merge.list -Oz -o UMAG1.POP.FREQ.vcf.gz

bcftools index --tbi UMAG1.POP.FREQ.vcf.gz
```

Note S21. Creating synthetic diploid reads.

To create our synthetic reads, we used three existing phased genome assemblies created from the offspring of divergent parents. In each of these three trios, the sex of the F1 that was sequenced dictated the sex chromosome(s) that could be assembled (Rice et al. 2020; Koren et al. 2018; Low et al. 2020; Heaton et al. 2021; Oppenheimer et al. 2021). When a chromosome was missing from a haploid assembly, we added the missing chromosome from another assembly (**Table S11**), ensuring consistency with the autosomes, sex chromosomes, and mitochondrial genome. For each of the six haploid genomes, we generated synthetic reads using NEAT 3.2 (Stephens et al. 2016). The parameters used were: -R 150, -c 15, -E 0, -M 0, --pe 350 70, --rng 1234567. These simulate 15× coverage of each haplotype without error, similar to standard Illumina paired-end data. However, the per-base error rate of the assemblies was higher than in the raw SRS data, meaning our sampled synthetic reads are noisy. The reads from each chromosome are then concatenated into a single pair of files representing a diploid genome. The reads are then shuffled to remove the linear genomic order used to generate them to avoid issues during the alignment stage.

```
python3 gen_reads.py -r $RefName.$c.fa -R $ReadLen -c $COV -E $ErrorRate \
-M $MutationRate --pe $Pe --rng $RNG --bam -o OUT/${OUTPREFIX}.${COV}.$c
cat OUT/${RefName}.${COV}.*_read1.fq.gz >OUT/${RefName}.${COV}.1.fastq.gz
cat OUT/${RefName}.${COV}.*_read2.fq.gz >OUT/${RefName}.${COV}.2.fastq.gz
File1=OUT/${RefName}.${COV}.1.fastq.gz
File2=OUT/${RefName}.${COV}.2.fastq.gz
Rand1="OUT/RAND/${RefName}.${COV}.1.fastq"
Rand2="OUT/RAND/${RefName}.${COV}.2.fastq"
paste <(zcat $File1) <(zcat $File2) | paste - - - - | shuf | awk -F'\t' \
-v r1=$Rand1 -v r2=$Rand2 '{OFS="\n"; print $1,$3,$5,$7 > r1; \
print $2,$4,$6,$8 > r2}'
```

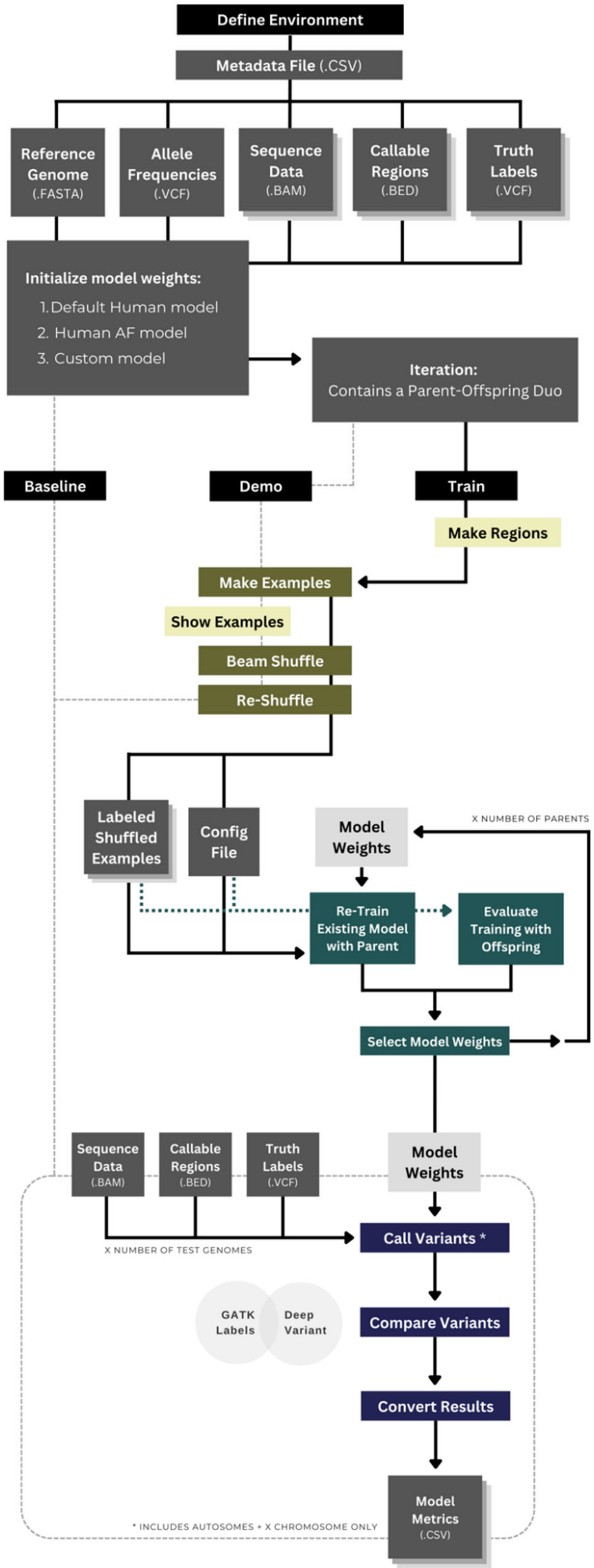
Define Environment
Metadata File (.CSV)
Reference Genome (.FASTA)
Allele Frequencies (.VCF)
Sequence Data (.BAM)
Callable Regions (.BED)
Truth Labels (.VCF)
Initialize model weights:
1. Default Human model
2. Human AF model
3. Custom model
Iteration:
Contains a Parent-Offspring Duo
Baseline
Demo
Train
Make Regions
Make Examples
Show Examples
Beam Shuffle
Re-Shuffle
Labeled Shuffled Examples
Config File
Model Weights
X NUMBER OF PARENTS
Re-Train Existing Model with Parent
Evaluate Training with Offspring
Select Model Weights
Sequence Data (.BAM)
Callable Regions (.BED)
Truth Labels (.VCF)
Model Weights
Call Variants *
X NUMBER OF TEST GENOMES
GATK Labels
Deep Variant
Compare Variants
Convert Results
Model Metrics (.CSV)
* INCLUDES AUTOSOMES + X CHROMOSOME ONLY

Figure S33. Workflow diagram for the TrioTrain pipeline.
(previous page) Two iterations are performed for each trio: one for each parent. Both iterations use replicates of the same offspring genome for selecting model weights that maximize the F1 score. A training iteration consists of three stages:

(1) *Data Preparation: (yellow) Enables genome-wide shuffling with SLURM*.
    Make Regions: Each genome is divided into a set of regions. By default, a region should produce 200,000 or fewer examples so that shuffling fits within memory more effortlessly.
    Make Examples: For each region, genome-wide labeled examples are created.
    Beam Shuffle: For each region, genome-wide shuffling occurs in parallel.
    Re-Shuffle: The per-CPU shards are merged into per-region tfrecord files; the per-region file order is randomized, and then the paths are written to a new config file.

(2) Iterative Re-Training: (green) Starts with an existing model checkpoint to sequentially re-train DeepVariant across all trios.
    Train: Shuffled, labeled, merged, and randomized tfrecord files from the parent genome are incrementally provided to the model in groups set by batch size (32) while adjusting model weights using a learning rate (0.005).
    Evaluate: As new model weights are produced, genetic variants are called using shuffled, labeled, merged, and randomized examples produced from the offspring's genome. Tune the model to accurate genotype inherited variants.
    Select: Each checkpoint's performance metrics are stratified by variant type and class. Optimal performance is based on evaluation with the F1 score to select one checkpoint per iteration.

(3) *Testing A Model: (blue) Assess how well the model performs in genomes that the model has not previously seen.*
    Call Variants: Use each selected checkpoint for variant calling in genomes outside the trios.
    Compare Variants: Use hap.py to assess the new model's variants against a Truth VCF.
    Convert Results: Process pattern combinations from hap.py into raw metric counts.

Note S21. Required Inputs for TrioTrain

Once a starting point is selected at the warm-starting weights, the TrioTrain pipeline automatically prepares each genome's labeled, shuffled examples in a trio based on a pipeline configuration input file. This metadata file (.csv) contains descriptive information such as mean coverage and the absolute paths to all required input files. Training order is dictated by the row order within the metadata file, while the first parent is set to either "Mother" or "Father" via the `--first-parent` flag using TrioTrain.

The metadata file is provided as a command line flag and contains the pedigree and the local file structure for all the input files necessary for re-training DeepVariant. An example of the metadata file can be viewed at the following link:
https://docs.google.com/spreadsheets/d/1hSWD0a5PrWXTQKW4-Q6MTqm-kuI8Bs8cx3kB8u0GEOs/edit?usp=sharing

When using TrioTrain, the following assumptions are made:
(1) Each row corresponds to one complete family trio to use in two TrioTrain iterations.
(2) Row order determines the sequential order of how data is used during training.
(3) The number corresponding to a specific test genome (e.g., Test3) does not correspond to the testing order, as testing is performed in parallel.

At a minimum, the pipeline expects the metadata file to have two rows and 24 columns. The minimum rows include a header row of column names, where the second row represents a single trio for re-training. Additional rows correspond to other trios. The first two columns define the order in which data will be given to the model and provide a unique name for each iteration. The following seven columns describe the trio used by providing the sample ID and, optionally, a separate lab ID for the child, father, and mother, as well as the offspring's sex. The following three columns provide the reference genome, the optional population allele frequency VCF file, and the optional regions BED file. The last nine columns provide the input files for the child, father, and mother by providing the absolute paths to three files per genome (BAM/TruthVCF/BED). The last three required columns contain the input files required for each test genome, with three files per test genome (BAM/TruthVCF/BED). Further tests can be achieved by adding three columns for each additional genome. Complete details about the required data and their formats are described further in the TrioTrain user guide: https://jkalleberg.github.io/DV-TrioTrain/user-guide/usage_guide/#assumptions

Note S22. Implementing training on a SLURM-based cluster*.*

The DeepVariant software has two components: one for genotyping samples and the other for producing new models (**Figure S33**). While the variant caller is packaged as a single command executed from a Docker container, the training components are not. Additionally, some training steps are incompatible with SLURM-based HPC clusters. Considering these hurdles, we developed TrioTrain for species-agnostic model extension with new data. We automated repeated training rounds from a single command on research computing hardware by re-designing a previously intractable step, the shuffling approach for randomizing genomic order (**Figure S34**). Each candidate variant results in a multi-channel tensor record file called an example. Channels represent features commonly found in pileup alignment images. The primary difference for training is the addition of an extra channel containing the label (truth genotype). Examples are created in linear genomic order and stored on disk. The total number of examples produced depends on genome divergence from the reference. For example, a typical bovine genome can produce 8–10 million examples, while Bison genomes produce around 30 million (**Table S8**). Shuffling is required because training assumes the current example is independent of prior examples. However, achieving randomization of several million images is not trivial.

Our biggest hurdle was ensuring the labeled examples represented a complete genome yet fit within memory. TrioTrain implements a SLURM-compatible approach that distributes a genome into buckets called regions to handle millions of images created per genome. Before generating examples, we calculate a genome-specific number of regions based on the total truth variants. Each region is defined in a 0-based BED file, where sequential regions overlap by 1b. While the number of genome-wide examples can vary significantly, a region samples the chromosomes proportionally yet produces approximately 200,000

examples. These BED files are used when generating examples, resulting in distributed, independent SLURM jobs for each region.

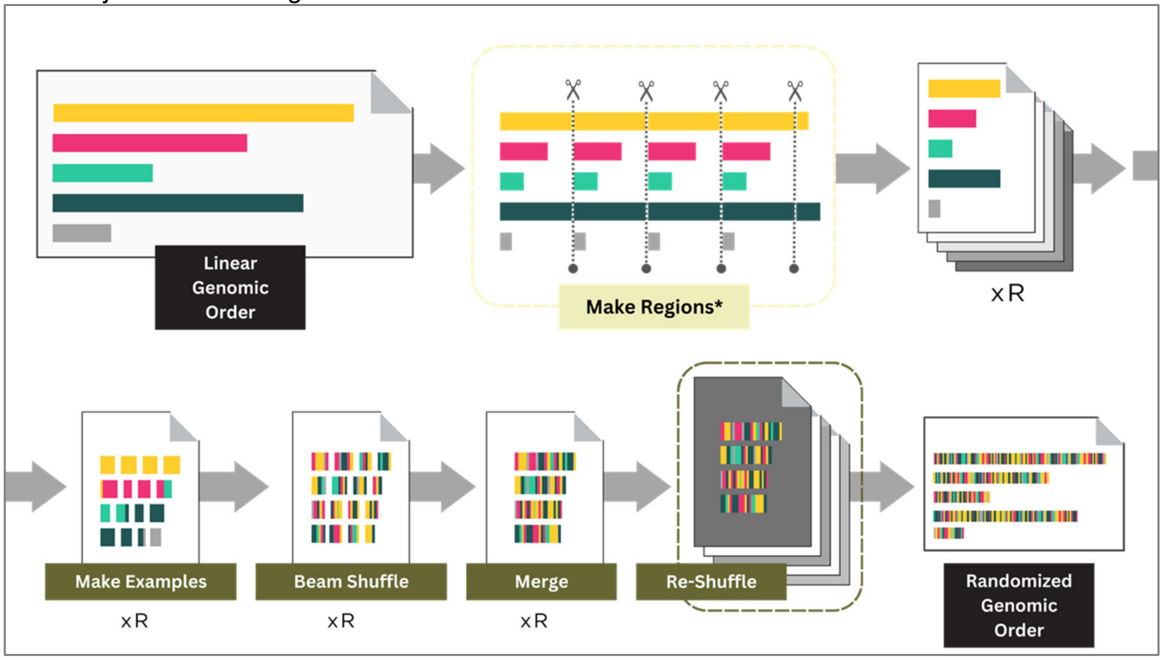

Figure S34. Region *shuffling* *a*pproach.
TrioTrain automatically determines how many regions (R) are required to split a complete genome. This calculation is based on a user-defined maximum number of examples per region (default = 200,000), the expected number of examples created per variant, which varies between species (default=1.5), and the number of variants in the corresponding truth VCF. TrioTrain first creates R overlapping BED files containing a proportional sample from the autosomes and the X Chromosome. Next, within-region labeled examples are made in parallel. To minimize linear genomic order, each genome-wide set of files undergoes shuffling with Apache Beam. The shuffled within-region labels are then concatenated. Finally, the region order is randomized, reducing the chances of providing the model regions sequentially. The respective config file for the re-shuffled examples is given to the model for either training or evaluation, depending on the genome.

Note S23. Defining regions for shuffling.

We calculate the number of regions per genome, RG, as follows. First, TrioTrain requires two user-provided constant values, N and E, where N is the approximate number of examples in each region (default = 200,000). E is the estimated number of examples generated per variant (default = 1.5, estimated using --demo mode based on the number of examples produced from a single chromosome, rounded up to the nearest 0.5). TrioTrain will also internally calculate three additional parameters: T_g, C, and B. T_g is the total number of variants within the genome-specific truth VCF C is a set of n values, where n is the total number of chromosomes. For C, the values represent the total length (bp) for each chromosome (c) as a proportion of the total genome length (bp). These values are obtained from the Picard reference dictionary file generated automatically by TrioTrain. The series is calculated as $C = \{c_i \ldots c_n\} / \sum_{i=1}^{n} C$. Next, we calculate the number of variants per base pair, as $B = \frac{T_g}{\sum_{i=1}^{n} C}$.

We assume that variants are equally distributed across the genome. TrioTrain then creates three series of n values, where n is the number of chromosomes. First, we calculate the number of variants per chromosome as $V = \{v \ldots v_n\} = T_g * \{c_i \ldots c_n\}$. Next, we calculate the length (bp) to sample from each chromosome so that each region BED file will contain the same amount of content per chromosome as the complete genome as $L = \{l \ldots l_n\} = \frac{\{v \ldots v_n\}}{B}$. Lastly, we obtain the number of regions, or B.E.D. files to create as $R_g = \frac{\{c \ldots c_n\}}{\{l \ldots l_n\}} + 1$. We add one additional region for any division remaining across chromosomes. TrioTrain then creates R_g BED files with chromosome number, start, and stop values based on $\{l \ldots l_n\}$ where the start of the subsequent region overlaps the prior region by one base pair. Each BED file is then passed to DeepVariant and executed within R_g SLURM jobs for the make_examples module.

Note S24. Checkpoints used for warm-starting training

Modeler builders should consider how the final model will be used before selecting truth data and a training starting point. For example, we prioritized including the Allele Frequency (AF) channel to use our bovine-trained model with legacy, low-coverage samples commonly found in animal genomics. However, the warm-starting checkpoint with TrioTrain is user-defined, ensuring species without population-scale resources to avoid including this channel.

The default model checkpoint for DeepVariant v1.4.0, which was used to initialize weights for re-training, was obtained from:
https://console.cloud.google.com/storage/browser/deepvariant/models/DeepVariant/1.4.0/DeepVariant-inception_v3-1.4.0+data-wgs_standard.

Note that the `--make_examples_extra_args` flag is specific to the WGS Allele Frequency (WGS.AF) model and the bovine model checkpoints and, thus, not used with the default WGS model or DeepTrio.

The population allele frequency model for DeepVariant v1.4.0, which was used to compare against our new model, was obtained from: https://console.cloud.google.com/storage/browser/brain-genomics-public/research/allele_frequency/pretrained_model_WGS/1.4.0;tab=objects?pageState=(%22StorageObjectListTable%22:(%22f%22:%22%255B%255D%22))&prefix=&forceOnObjectsSortingFiltering=false

Note S25. Supplemental Data and Code

Reference Genome

The ARS-UCD1.2_Btau5.0.1Y bovine reference genome has been deposited in Zenodo as DOI: 10.5281/zenodo.15482484. Companion files necessary for re-training using bovine genomes include:

- For constraining training to the autosomes and X Chromosome (autosomes_withX.bed)
- For region-shuffling within TrioTrain, a copy of the reference dictionary file (.dict) created with picard (https://github.com/broadinstitute/picard),

1000 Bull Genomes (1kBulls) Data Access
Run8 variation callset:

- Analysis Accession: ERZ1738264
- Link: https://www.ebi.ac.uk/ena/browser/view/ERZ1738264

Run9 variation callset:

- Analysis Accession: ERZ14211345
- Link https://www.ebi.ac.uk/ena/browser/view/ERZ14211345

Selected TrioTrain Checkpoint
The checkpoint produced with the TrioTrain pipeline (checkpoint 28, modle.ckpt-282383) has been deposited in Zenodo as DOI: 10.5281/zenodo.15482484. This file is compatible with DeepVariant (v1.4) for short-read, whole-genome-sequencing (WGS) data. To use this checkpoint to perform single-sample variant calling, the following command template is provided; note: **bolded flags** are required to deviate from the default human-trained checkpoint:

```
BIN_VERSION_DV="1.4.0"
apptainer run
 -B /usr/lib/locale/:/usr/lib/locale/,
    ${YOUR_REF_DIR}:/ref_dir/,
    ${YOUR_BAM_DIR}:/bam_dir/,
    ${YOUR_OUTPUT_DIR}:/out_dir/,
    ${YOUR_CUSTOM_MODEL_DIR}:/start_dir/,
    ${YOUR_POPVCF_DIR}:/pop_dir/
Deepvariant_${BIN_VERSION_DV}.sif
    /opt/deepvariant/bin/run_deepvariant
    --model_type=WGS
    --ref=/ref_dir/ARS-UCD1.2_Btau5.0Y.fa
    --reads=/bam_dir/${YOUR_BAM}
    --output_vcf=/out_dir/${YOUR_OUTPUT_VCF}
    --num_shards=$(nproc)
    --customized_model=/start_dir/model.ckpt-282383
    --make_examples_extra_args
"use_allele_frequency=true,population_vcfs=/pop_dir/UMAG1.POP.FREQ.vcf.gz"
```

Genome-in-a-Bottle (GIAB) Benchmarking Data
To ensure reproducibility, the exact steps used to obtain raw data and source code listed below are contained within two bash scripts available on GitHub:

1. https://github.com/jkalleberg/DV-TrioTrain/blob/cce8f5d62558141032faa2eaa8c4f4c012eaf97e/scripts/setup/download_GIAB.sh
2. https://github.com/jkalleberg/DV-TrioTrain/blob/d8079bb1710d66ed6d616866dda3c0d87101d05b/scripts/setup/download_GIAB_stratifications.sh

The GRCh38 human reference genome (GRCh38_no_alt_analysis_set.fasta) was obtained from: https://ftp.ncbi.nlm.nih.gov/genomes/all/GCA/000/001/405/GCA_000001405.15_GRCh38/seqs_for_alignment_pipelines.ucsc_ids/

The GIAB benchmarks (v4.2.1) are specific to the GRCh38 reference genome. The parent directory for files for both trios is available here: https://ftp-trace.ncbi.nlm.nih.gov/ReferenceSamples/giab/release/. For example, individual benchmark variant callsets (VCFs) and corresponding 'ConfidentRegions' (BED) files are available for HG002 here: https://ftp-trace.ncbi.nlm.nih.gov/ReferenceSamples/giab/release/AshkenazimTrio/HG002_NA24385_son/NISTv4.2.1/GRCh38/

Short-read Illumina WGS for all six GIAB individuals was previously aligned to the GRCh38 reference genome. The processed files (BAM) were obtained from the US NIST FTP site; complete paths and MD5 checksums for these files are described here:
https://github.com/genome-in-a-bottle/giab_data_indexes/blob/c4d3b95c2ebf14c175151e4723f82e8980722e90/AshkenazimTrio/alignment.index.AJtrio_Illumina_2x250bps_novoalign_GRCh37_GRCh38_NHGRI_06062016

Stratification files for HG002 (v3.5) given to hap.py are specific to the GRCh38 reference genome. The parent directory for these files is available here: https://ftp-trace.ncbi.nlm.nih.gov/ReferenceSamples/giab/release/genome-stratifications/v3.5/ Additional description of these files is described here: https://github.com/genome-in-a-bottle/genome-stratifications Note that the version number described on GitHub (v3.1) differs from the version of the files on the NIST FTP site (v3.5), which were used for stratification in HG002.

The population VCF for human genomes was produced by the 1000 Genomes Project (1KGP). However, to replicate the process described in the DeepVariant documentation, we obtained a copy of these data from the DeepVariant Google Cloud Storage (GCP) bucket. Note that the per-chromosome VCFs obtained from GCP were subsequently merged into a single VCF. Access to these data will require an active account with Google (i.e., Gmail): https://console.cloud.google.com/storage/browser/brain-genomics-public/research/cohort/1KGP/cohort_dv_glnexus_opt/v3_missing2ref

Source Code Availability
The approach for shuffling labeled examples is specific to the version of DeepVariant (v1.4). This process relies on an Apache Beam python script obtained directly from the original developers here:
https://raw.githubusercontent.com/google/deepvariant/r1.4/tools/shuffle_tfrecords_beam.py

Note S26. Evaluating performance in bovine genomes.

After a training iteration, the selected best checkpoint is immediately used with the one-step variant caller as a custom checkpoint. Although we generate truth labels for these samples, these are not given to the model to call variants but used to assess how the new model changed the resulting callset. DeepVariant is run using:

```
/opt/deepvariant/bin/run_deepvariant \
      --model_type WGS \
      --ref /ref_dir/ARS-UCD1.2_Btau5.0.1Y \
      --reads /bam_dir/<test_genome>.cram \
      --customized_model /start_dir/<new_checkpoint>.ckpt \
      --make_examples_extra_args=
      "use_allele_frequency=true,population_vcfs=/popVCF_dir/<allele_frequenc
      ies>.vcf \
      --output_vcf /out_dir/<prefix>.vcf.gz
      --intermediate_results_dir /out_dir/tmp/<prefix> \
      --num_shards (nproc - 1) \
      --exclude_regions Y
```

The resulting compressed VCF is then passed to hap.py (v0.3.12), the tool recommended by the Global Alliance for Genomics and Health (GA4GH) Benchmarking Team (Krusche et al. 2019) and used the vcfeval engine for comparison (Cleary et al. 2015). Although we used software designed for benchmarking VCFs, we could only calculate performance metrics relative to the GATK-derived genotypes we used as truth labels. As such, a True Positive (**TP**) is an identical variant between GATK and DV, a False Positive (**FP**) is a variant missing from our GATK truth labels but detected by DV, and a False Negative (**FN**) is a variant present in our GATK truth labels that DeepVariant missed. We note that hap.py uses an obsolete version of Python (v2.7), so we obtained a working copy within an existing container from Docker (docker://jmcdani20/hap.py:v0.3.12).

For all 30 iterations, we run hap.py in parallel as separate SLURM jobs for each testing genome, with the number of testing samples varying by testing phase. We execute hap.py using:

```
/opt/hap.py/bin/hap.py \
      /truth/<truthVCF> \
      /query/<custom_model_DVoutputVCF> \
      -r /ref/ARS-UCD1.2_Btau5.0.1Y.fa \
      -f /callable/<truthBED> \
      -o /output/<prefix> \
      --write-counts \
      --keep-scratch \
      --scratch-prefix /output/scratch \
      --engine vcfeval \
      --threads $(nproc - 1) \
      --location <autosomes_withX.file>
```

We then used custom Python scripts to process the output VCF from hap.py into CSV files with summary metrics, including precision, recall, and F1 score, and stratified by variant class (SNV vs. indels). We calculated precision as $\mathrm{TP} / (\mathrm{TP} + \mathrm{FP})$ and recall as $\mathrm{TP} / (\mathrm{TP} + \mathrm{FN})$. From these, we calculated F1 score as $2 * [(\mathrm{precision} * \mathrm{recall}) / (\mathrm{precision} + \mathrm{recall})]$. After all tests and comparisons with hap.py are complete, the per-sample CSV files with these metrics are concatenated with those from the entire phase-specific testing group. The group's performance metrics are then used to assess model performance within bovine genomes. The F1 score for all tests from the bovine training iterations was plotted using Python (v3.12); see source code for figure generation scripts (https://github.com/jkalleberg/DV-TrioTrain/blob/main/triotrain/visualize/final_manuscript_figures.py).

Note S27. Calculating Mendelian Inheritance Errors

First, we created a reference format SDF file required by rtg-tools for the GRCh38 reference and the ARS-UCD1.2_Btau5.0.1Y reference (Rosen et al. 2020). For example, for the human reference, we used:

```
rtg format \
      -o ./triotrain/variant_calling/data/GIAB/reference/rtg_tools/ \
      ./triotrain/variant_calling/data/GIAB/reference/GRCh38_no_alt_analysis_
      set.fasta
```

A pedigree file required for rtg-tools Mendelian was created for each trio using a custom Python script using trio metadata (UMAGv1 cohort for bovine, NIST for human). Next, we converted the single-sample VCFs from the various DeepVariant models to BCFs using BCFtools convert (v1.14), with an index created using BCFtools index (v1.14). We then created a trio VCF constrained to the autosomes and X Chromosome only using BCFtools merge (v1.14) to combine the per-sample BCFs, where sample order was defined as offspring, father, and mother. The trio BCF was also indexed using BCFtools (v1.14). These files were then passed to rtg-tools using:

```
conda run --no-capture-output ./miniconda_evns/beam_v2.30 \
      rtg mendelian \
      --input <trioVCF> \
      --output <mieVCF> \
      --template <referenceSDF> \
      --pedigree <ped.txt>
```

We note that this results in a VCF containing only the PASS variants by default. Additionally, the variants detected in the offspring with missing genotypes in either parent result in an “uncertain” designation and, thus, are excluded from calculations. The reported MIE rate is then extracted from the log file for each trio, with the results plotted with Python (v3.12); see source code for figure generation scripts (https://github.com/jkalleberg/DV-TrioTrain/blob/main/triotrain/visualize/final_manuscript_figures.py).

REFERENCES

Bolger AM, Lohse M, Usadel B. 2014. Trimmomatic: A flexible trimmer for Illumina sequence data. *Bioinformatics* 30: 2114–2120.

Cleary JG, Braithwaite R, Gaastra K, Hilbush BS, Inglis S, Irvine SA, Jackson A, Littin R, Rathod M, Ware D, et al. 2015. Comparing Variant Call Files for Performance Benchmarking of Next-Generation Sequencing Variant Calling Pipelines. *bioRxiv* Aug. 3: 023754. http://biorxiv.org/content/early/2015/08/03/023754.abstract.

Elsik CG, Tellam RL, Worley KC, Gibbs RA, Muzny DM, Weinstock GM, Adelson DL, Eichler EE, Elnitski L, Guigó R, et al. 2009. The genome sequence of taurine cattle: A window to ruminant biology and evolution. *Science (1979)* 324.

Hayes BJ, Daetwyler HD. 2018. 1000 Bull Genomes Project to Map Simple and Complex Genetic Traits in Cattle: Applications and Outcomes. *Annu Rev Anim Biosci* 7: 89–102.

Heaton MP, Smith TPL, Bickhart DM, Vander Ley BL, Kuehn LA, Oppenheimer J, Shafer WR, Schuetze FT, Stroud B, Mcclure JC, et al. 2021. A Reference Genome Assembly of Simmental Cattle, Bos taurus taurus. *Journal of Heredity* 112: 184–191.

Koren S, Rhie A, Walenz BP, Dilthey AT, Bickhart DM, Kingan SB, Hiendleder S, Williams JL, Smith TPL, Phillippy AM. 2018. De novo assembly of haplotype-resolved genomes with trio binning. *Nat Biotechnol* 36: 1174–1182.

Krusche P, Trigg L, Boutros PC, Mason CE, De La Vega FM, Moore BL, Gonzalez-Porta M, Eberle MA, Tezak Z, Lababidi S, et al. 2019. Best practices for benchmarking germline small-variant calls in human genomes. *Nat Biotechnol* 37: 555–560. http://dx.doi.org/10.1038/s41587-019-0054-x.

Low WY, Tearle R, Liu R, Koren S, Rhie A, Bickhart DM, Rosen BD, Kronenberg ZN, Kingan SB, Tseng E, et al. 2020. Haplotype-resolved genomes provide insights into structural variation and gene content in Angus and Brahman cattle. *Nat Commun* 11.

Oppenheimer J, Rosen BD, Heaton MP, Vander Ley BL, Shafer WR, Schuetze FT, Stroud B, Kuehn LA, McClure JC, Barfield JP, et al. 2021. A Reference Genome Assembly of American Bison, Bison bison bison. *J Hered* 112: 174–183.

Rainio O, Teuho J, Klén R. 2024. Evaluation metrics and statistical tests for machine learning. *Sci Rep* 14.

Rice ES, Koren S, Rhie A, Heaton MP, Kalbfleisch TS, Hardy T, Hackett PH, Bickhart DM, Rosen BD, Ley B Vander, et al. 2020. Continuous chromosome-scale haplotypes assembled from a single interspecies F1 hybrid of yak and cattle. *Gigascience* 9: 1–9.

Rosen BD, Bickhart DM, Schnabel RD, Koren S, Elsik CG, Tseng E, Rowan TN, Low WY, Zimin A, Couldrey C, et al. 2020a. De novo assembly of the cattle reference genome with single-molecule sequencing. *Gigascience* 9: 1–9.

Rosen BD, Bickhart DM, Schnabel RD, Koren S, Elsik CG, Tseng E, Rowan TN, Low WY, Zimin A, Couldrey C, et al. 2020b. De novo assembly of the cattle reference genome with single-molecule sequencing. *Gigascience* 9.

Rowan TN, Hoff JL, Crum TE, Taylor JF, Schnabel RD, Decker JE. 2019. A multi-breed reference panel and additional rare variants maximize imputation accuracy in cattle. *Genetics Selection Evolution* 51: 1–16.

Stephens ZD, Hudson ME, Mainzer LS, Taschuk M, Weber MR, Iyer RK. 2016. Simulating next-generation sequencing datasets from empirical mutation and sequencing models. *PLoS One* 11: 1–18.
